# Quantitative phosphoproteomics reveals the role of the AMPK plant ortholog SnRK1 as a metabolic master regulator under energy deprivation

**DOI:** 10.1038/srep31697

**Published:** 2016-08-22

**Authors:** Ella Nukarinen, Thomas Nägele, Lorenzo Pedrotti, Bernhard Wurzinger, Andrea Mair, Ramona Landgraf, Frederik Börnke, Johannes Hanson, Markus Teige, Elena Baena-Gonzalez, Wolfgang Dröge-Laser, Wolfram Weckwerth

**Affiliations:** 1 Department of Ecogenomics and Systems Biology, University of Vienna, Vienna, Austria; 2 Vienna Metabolomics Center (VIME), University of Vienna, Vienna, Austria; 3 Julius-von-Sachs-Institut, Julius-Maximilians-Universität Würzburg, Würzburg, Germany; 4 Plant Health, Plant Metabolism Group, Leibniz-Institute of Vegetable and Ornamental Crops, Großbeeren, Germany; 5 Institute of Biochemistry and Biology, University of Potsdam, Potsdam, Germany; 6 Department of Plant Physiology, Umeå University, Umeå, Sweden; 7 Instituto Gulbenkian de Ciência, Oeiras, Portugal

## Abstract

Since years, research on SnRK1, the major cellular energy sensor in plants, has tried to define its role in energy signalling. However, these attempts were notoriously hampered by the lethality of a complete knockout of SnRK1. Therefore, we generated an inducible amiRNA::SnRK1α2 in a *snrk1α1* knock out background (*snrk1α1*/*α2*) to abolish SnRK1 activity to understand major systemic functions of SnRK1 signalling under energy deprivation triggered by extended night treatment. We analysed the *in vivo* phosphoproteome, proteome and metabolome and found that activation of SnRK1 is essential for repression of high energy demanding cell processes such as protein synthesis. The most abundant effect was the constitutively high phosphorylation of ribosomal protein S6 (RPS6) in the *snrk1α1*/*α2* mutant. RPS6 is a major target of TOR signalling and its phosphorylation correlates with translation. Further evidence for an antagonistic SnRK1 and TOR crosstalk comparable to the animal system was demonstrated by the *in vivo* interaction of SnRK1α1 and RAPTOR1B in the cytosol and by phosphorylation of RAPTOR1B by SnRK1α1 in kinase assays. Moreover, changed levels of phosphorylation states of several chloroplastic proteins in the *snrk1α1*/*α2* mutant indicated an unexpected link to regulation of photosynthesis, the main energy source in plants.

As sessile organisms, plants have to cope with ever changing environmental conditions. Hence, they have developed strategies to sense and to acclimate to unfavourable circumstances. Such stress situations are often linked to the availability of energy. When energy is not limited, plants produce energy-rich compounds and direct resources to the synthesis of storage compounds and growth. In contrast, under stressful conditions nutrient remobilization and growth arrest, as part of a vast metabolic reprogramming, are the dominating processes. Sucrose non-fermenting related kinase 1 (SnRK1) and its orthologs, the AMP-dependent protein kinase (AMPK) and sucrose non-fermenting 1 (SNF1) kinase in mammals and yeast, respectively, are conserved signalling components that significantly contribute to the maintenance of cellular energy homeostasis^1^,^2^. In Arabidopsis two genes, *SnRK1α1 (AKIN10*, AT3G01090) and *SnRK1α2 (AKIN11*, AT3G29160), encode the catalytic α-subunit of the SnRK1 complex. In the mammalian systems, AMPK was shown to sense the cellular energy status via the AMP/ATP ratio^3^. AMP can affect AMPK activity in two ways. First, it can allosterically modify AMPK and influence its catalytic activity. Second, AMP reduces the dephosphorylation rate and inactivation of AMPK by protein phosphatase 2C (PP2C) whereas ATP promotes this event. Xiao and colleagues also showed that ADP bound to AMPK prevents the kinase from dephosphorylation although it is not inducing allosteric activation^4^. In plants, AMP has not been shown to regulate SnRK1 allosterically but it is rather affecting its rate of dephosphorylation^2^. As indicators of the plants’ energy status, carbohydrates were shown to play a central role^5^. For example, high concentration of sugar phosphates, e.g. glucose-6-phosphate (G6P)^6^,^7^ and trehalose-6-phosphate (T6P)^7^,^8^,^9^,^10^, were discussed to indicate energy availability. Recent studies have shown that these phosphorylated sugars inhibit SnRK1 activity which directly connects the carbohydrate homeostasis with the SnRK1 signalling network^11^,^12^. Another interaction of SnRK1 with adenosine kinase was recently revealed proposing a complementary mechanism of regulation^13^.

A pivotal role of SnRK1 in linking stress, development and sugar signalling has been described on the level of gene expression, indicating a crucial regulatory influence on global plant metabolism, energy balance and growth^14^. In response to various stresses and energy limitation, SnRK1 affects transcriptional processes leading to a metabolic reprogramming^14^,^15^,^16^. SnRK1 has been shown to regulate several biosynthetic enzymes via post-translational modification, i.e. phosphorylation. Examples for such targets are the HMG-CoA reductase (HMG), sucrose phosphate synthase (SPS), nitrate reductase (NR)^17^, 6-phosphofructo-2-kinase/2,6-fructose-bis-phosphatase (F2KP)^18^ and trehalose-6-phosphate synthase (TPS)^19^. SnRK1 phosphorylates these enzymes at conserved SnRK1 phosphorylation consensus sequences, which consist minimally of a phosphorylated Ser or Thr residue, a hydrophobic residue at −5 and +4 position and a basic residue at position −3 or −4^20^,^21^. While many of the SnRK1 phosphorylation target studies have been done *in vitro*, nowadays, mass spectrometry (MS) based large-scale phosphoproteomics experiments enable the detailed and comprehensive analysis of *in vivo* phosphorylation of hundreds of putative targets in a single measurement.

Like SnRK1, target of rapamycin (TOR) is a central regulator of energy metabolism in eukaryotic organisms^22^,^23^,^24^,^25^. TOR signalling has been shown to play an essential role in central processes like embryogenesis, growth regulation, flowering and senescence, promoting anabolic functions, ribosome biogenesis, protein synthesis, and growth^22^,^23^,^26^,^27^,^28^,^29^. From mammalian systems it is known that the TOR resultant growth effects are mediated through the TOR-dependent phosphorylation of ribosomal S6 kinase (S6K) and eukaryotic translation initiation factor 4E-binding protein (4E-BP)^30^. Rapamycin was shown to inhibit the phosphorylation of Thr^449^ of S6K1 demonstrating further the link between S6K and TOR^31^. Both for mammalian systems and plants it was shown that S6K is targeting the ribosomal protein S6 (RPS6)^30^,^32^. The Arabidopsis *rps6* mutant displayed reduced cell size and delayed growth and flowering^26^. Further, the cell size of callus tissue from transgenic plants overexpressing S6K1 were found to be bigger^33^ whereas a hemizygous *s6k1s6k2*/++ mutant had a higher proportion of smaller cells compared to the wild type while the cell number remained unaltered^34^.

Based on studies in mammalian cells, it is known that the TOR pathway is inhibited by energy depriving conditions and by AMPK-driven phosphorylation of the regulatory-associated protein of TOR (RAPTOR)^35^. However, it is still unclear if this connection exists in plants, but there is evidence that TOR and SnRK1 pathways have antagonistic roles depending on the energy availability (for review, see refs 23,36,37).

In summary, AMPK-/SNF1-/SnRK1- and TOR-related signalling networks affect a multitude of central processes involved in embryogenesis, growth and development of various eukaryotes. Hence, it is not surprising that the metabolic output as well as the identification of involved signalling compounds remains a complex task, which demands a combination of comprehensive experimental and theoretical methods for its conclusive analysis. The aim of the present study was to depict a comprehensive picture of SnRK1-induced dynamics in the *Arabidopsis thaliana* phosphoproteome. For this purpose, we measured the phosphoproteome in *snrk1*α*1* single knockout (ko), overexpressor (OE) and *snrk1α1*/*α2* double knockdown (kd) lines and identified and quantified more than 1000 phosphoproteins and 2000 phosphorylation sites. An intriguing observation was the upregulation of RPS6 *in vivo* phosphorylation in *snrk1α* mutants. The molecular link between SnRK1 and TOR pathways in plants was so far only speculative. Based on the observation of high RPS6 phosphorylation in *snrk1α* mutants and the reports from mammalian systems^35^, our finding indicates the antagonistic crosstalk of SnRK1- and TOR-signalling also in plants. Moreover, we found that SnRK1 had an impact on eukaryotic translation initiation factor eIF5A phosphorylation which also indicates the importance of SnRK1 in regulating energy demanding protein translation. Besides, a novel link between SnRK1 signalling and chloroplast metabolism emerged from our data as we found several well-known chloroplast phosphoproteins significantly downregulated in the *snrk1α1*/*α2* mutant during the low energy syndrome (LES). Metabolomics analysis of the *snrk1α1*/*α2* mutant revealed a strong effect on mitochondrial metabolism. Altogether the data suggests an early and late SnRK1 control on a highly complex network of pathways in starvation conditions.

## Results

### SnRK1α mutants

**Figure 1:**
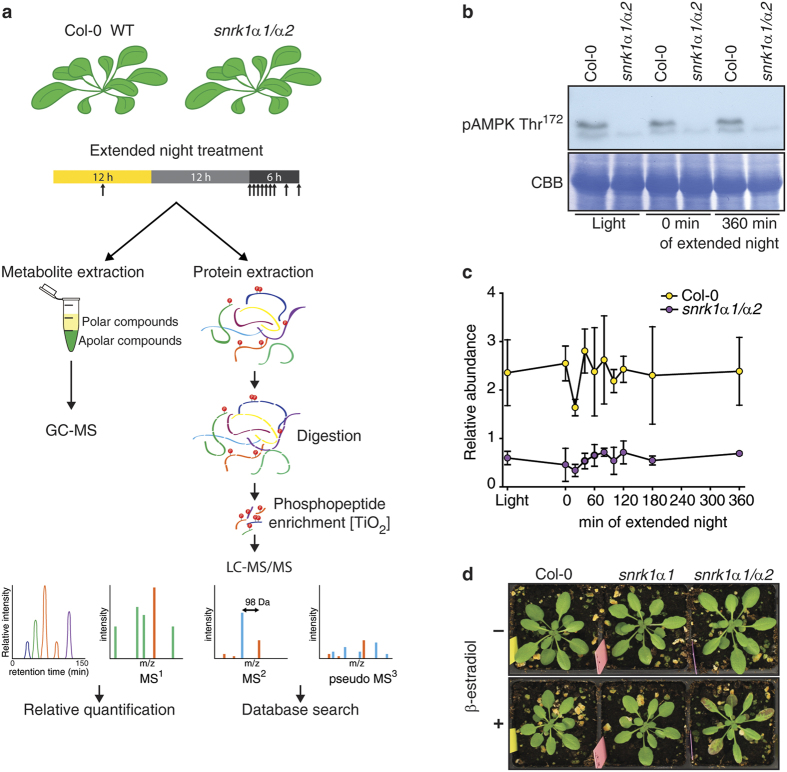
Experimental setup and knocking down the SnRK1α2. (**a**) Overview of the experimental setup for the analysis of the phosphoproteome of SnRK1 mutants. A dense time course (0, 20, 40, 60, 80, 100, 120, 180, and 360 min of extended night plus sample in the middle of the day) was sampled of the *snrk1α1*/*α2* mutant and the wild type during extended night. Metabolites were extracted with methanol:chloroform:water extraction solution, derivatized and measured with GC-MS. Proteins were isolated, digested and desalted by a combined C18/graphite carbon method. Subsequently, phosphopeptides were enriched by TiO_2_ MOAC and analysed by LC-MS/MS. (**b**) The expression of SnRK1α1 and SnRK1α2 in Col-0 and *snrk1α1*/*α2* in the time series experiment for 3 time points (Light, 0 min and 360 min of extended night) is shown with pAMPK Thr^172^ antibody, which recognizes both SnRK1α1 (upper band 61.2 kDa) and SnRK1α2 (lower band 58.7 kDa). (**c**) The abundance of T-loop phosphorylation is also shown by the level of DGHFLK[T^175/176^]SCGSPNYAAPEVISGK peptide in Col-0 and *snrk1α1*/*α2*. (**d**) Phenotype of Col-0 and SnRK1 mutant lines in 12 h light/12 h dark conditions. Knocking down of SnRK1α2 was started 22 days after germination (DAG) by spraying plants daily with 10 μM β-estradiol or mock solution without β-estradiol. Because SnRK1α2 protein is relatively stable its knocking down takes 5–6 days (Pedrotti *et al*. in preparation). This picture was taken 11 days (32 days after germination (DAG)) after the start of induction (phenotype development time course in Supplementary Fig. S1).

To characterize SnRK1-dependent signalling events we subjected plants that are lacking either SnRK1α1 or both SnRK1α1 and SnRK1α2 and plants that are overexpressing SnRK1α1 to extended night treatments that induce LES and consequently SnRK1 signalling. To this end, we used two different approaches. First, we treated soil-grown Arabidopsis plants overexpressing SnRK1α1 (lines OE1 and OE2), a *snrk1α1* T-DNA knock-out mutant (*snrk1α1-3*)^38^, and the corresponding Ler and Col-0 wild type plants, respectively, with 120 min of extended night. Here, phenotypic effects were rather weak most probably because of the functional compensatory effects of SnRK1α1 and SnRK1α2 subunits. Therefore, we decided, in a second approach, to employ a *snrk1α1-3* mutant expressing a β-estradiol inducible artificial micro (ami) RNA for *SnRK1α2* (hereafter referred as *snrk1α1*/*α2* mutant) as a way to deplete SnRK1 activity. With this approach we focused on the dynamics of SnRK1-dependent metabolic reprogramming, by comparing the phosphoproteome, the proteome and the metabolome of Col-0 and *snrk1α1*/*α2* plants at early and late time points during an extended night of increasing length (0, 20 40, 60, 80, 100, 12, 180, and 360 min) (Fig. 1a). The expression of SnRK1α1 in wild type and mutant plants was determined using Western blotting. SnRK1α1 is not present in the *snrk1α1* mutant and its expression is increased in OE lines (Supplementary Fig. S1). In the time series experiment we confirmed that SnRK1 is globally downregulated in the *snrk1α1*/*α2* double mutant. First, by utilizing the anti-AKIN10 antibody we show the absence of SnRK1α1 in the *snrk1α1*/*α2* mutant (Supplementary Fig. S1) and second, the downregulation of SnRK1α2 can be seen by exploiting phospho-AMPK Thr^172^ antibody that recognizes both SnRK1α1 (upper band 61.2 kDa) and SnRK1α2 (lower band 58.7 kDa) (Fig. 1b). Furthermore, quantifying the abundance of phosphorylated T-loop peptide DGHFLK[T^175^]SCGSPNYAAPEVISGK, which contains the Thr^175^ and Thr^176^ in SnRK1α1 and SnRK1α2, respectively, by LC-MS/MS based phosphoproteomics approach showed that the *snrk1α1*/*α2* mutant contained less than 25% of the phosphorylated T-loop peptide of wild type plants (Fig. 1c). Given that T-loop phosphorylation is essential for SnRK1 function^14^,^39^, the marked reduction of the T-loop phosphopeptide in *snrk1α1*/*α2* plants supports the overall decrease of SnRK1 in this mutant. While the single knock out of *snrk1α1* did not cause any visible phenotype (*snrk1α1* and non-induced *snrk1α1*/*α2* plants) *snrk1α1*/*α2* plants were accumulating anthocyanins and their growth was significantly reduced after knock down of SnRK1α2 (Fig. 1d and Supplementary Fig. S1). Similar effects were seen in previous studies when virus-induced gene silencing (VIGS) was applied to knock down both SnRK1α1 and SnRK1α2^14^,^38^.

### Comprehensive analysis of the *in vivo* phosphoproteome of the wild type and SnRK1-mutants revealed the phosphorylation of metabolic enzymes and transcription factors

**Figure 2:**
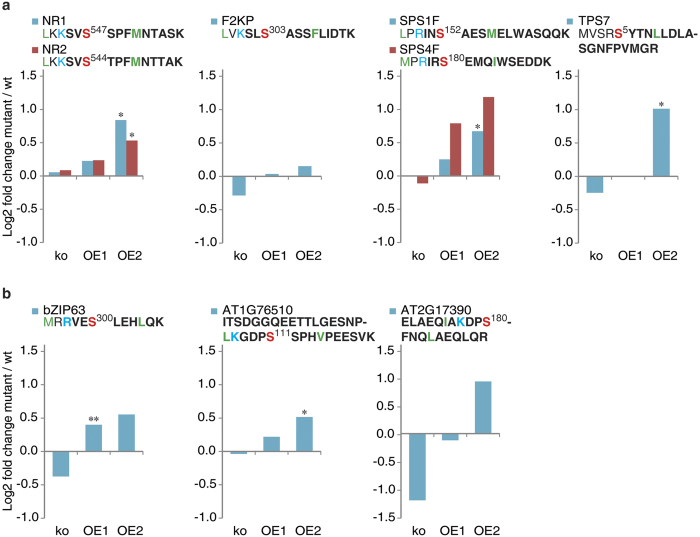
Changes in the phosphorylation levels of known and new SnRK1 targets. (**a**) Classical targets are NR, F2KP, SPS and TPS. New targets are bZIP63 and two uncharacterized transcription factors. Relative phosphopeptide abundances were normalized to the corresponding wild type (ko to Col-0 and OEs to Ler). Asteriks indicate significant differences in ttest between mutant and the wild type (*p < 0.05, **p < 0.01). The quantified SnRK1 consensus motif containing peptides are shown above the charts. The phosphorylated residue is shown in red, basic residue at the −3 or −4 position are indicated in blue and in green are shown the hydrophobic residues at the −5 and +4 positions. Tryptic peptides measured by LC-MS are in bold.

To characterize SnRK1-dependent phosphorylation events we subjected all *Arabidopsis thaliana* wild type and mutant plants to an extended night treatment that induces SnRK1 signalling. Metal-oxide affinity chromatography (MOAC) enrichment and liquid chromatography coupled to tandem mass spectrometry (LC-MS/MS) analysis of phosphopeptides according to established protocols in the lab were applied to leaf extracts of the treated plants^40^,^41^,^42^,^43^,^44^. For a deeper coverage of phosphoproteins we used a combination of C18/graphite desalting step before LC-MS/MS analysis^45^,^46^. In the first step, the phosphoprotein digest was desalted applying a C18 solid phase extraction (SPE). The C18 flow-through and wash fractions were collected and subjected to a second desalting step in which the graphite carbon desalting was applied. While the C18 technique captured 440 non-redundant phosphopeptides, 1255 phosphopeptides could be identified from the C18 flow-through desalted with graphite carbon. With these methods we identified and quantified altogether 2175 phosphopeptides in 1150 proteins from all samples (Supplementary Table S1). As shown earlier with *in vitro* experiments, HMG, NRs, SPSs, F2KP, and TPSs are potential targets for SnRK1α^17^,^18^,^19^. While we, or any previous study listed in the PhosPhAt^47^ and P3DB^48^, could not detect HMG N-terminal phosphorylation, we observed, that the phosphorylation status of NR2, NR1, SPS1F, SPS4F, and TPS7 was higher in the plants overexpressing SnRK1α1, especially in line OE2 than in the wild type (Fig. 2). On the contrary, the phosphorylation of these proteins was reduced in *snrk1α1* and in *snrk1α1*/*α2* mutants. At most of the extended night time points the phosphorylation status of these proteins was higher in wild type than in mutant lines, and the highest difference is about 2-fold. The typical target motif for SnRK1 phosphorylation is [MLVFI]x[RKH]xx[S]xxx[LFIMV], while a less preferred motif is [MLVFI][RKH]xxx[S]xxx[LFIMV]. This motif is nevertheless very similar to the phosphorylation motif of calcium dependent protein kinases (CDPKs) and has to be interpreted with care. When the early and late phases of extended night were examined, NR2 and F2KP were phosphorylated immediately after the onset of the treatment but have decreased phosphorylation level after 80 min of extended darkness in the wild type. In *snrk1α1*/*α2* mutant F2KP phosphorylation was decreased in the early phase (see Discussion). TPS5 and TPS7 were the most affected proteins. The phosphorylation of these proteins was about 2-fold higher in the early time points but also later at 120 min after extended night. Phosphopeptides corresponding to several transcription factors were also identified and differentially regulated in the OE1 and OE2 mutant compared to the wild type (Fig. 2). One example is bZIP63 which displayed higher phosphorylation levels at Ser^300^ in the SnRK1 overexpressor lines (Fig. 2). Recently, Mair *et al.*^38^ showed that Ser^300^ indeed is one of the target phosphorylation sites of SnRK1α1.

We also compared the SnRK1 *in vivo* phosphoproteome to the recently published phosphoproteome of a related protein kinase family SnRK2^49^. Here, the overlap of changed phosphoproteins ranged from 3–9 proteins depending on the experimental conditions, indicating that there is a major difference in these two signalling pathways (Supplementary Fig. S2).

### Motif analysis and putative phosphorylation targets of SnRK1

**Figure 3:**
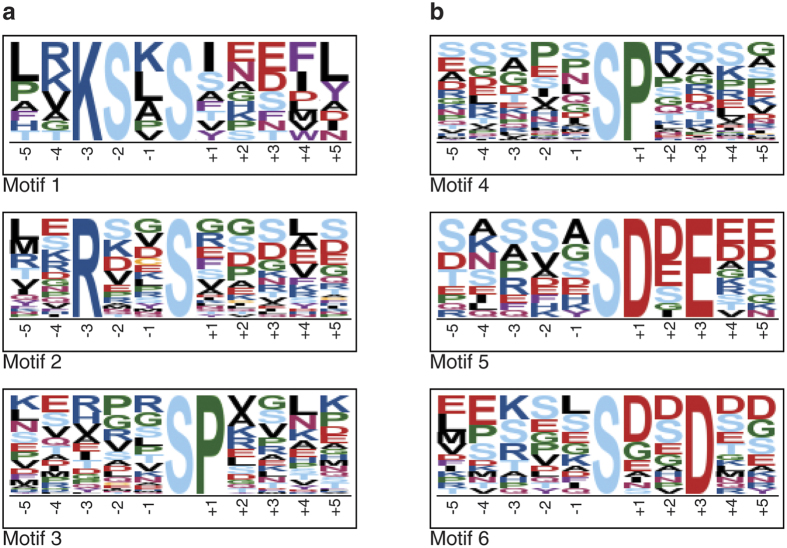
Identification of potential *in vivo* SnRK1 substrate candidates. Motif-x^50^ analysis was done for phosphopeptides that were at least 50% more abundant or significantly upregulated in the wild type (**a**) and in *snrk1α1*/*α2* mutant (**b**). p-value threshold was set to 0.000001 and at least 10 occurences were required. IPI Arabidopsis proteome was used as background proteome. For further details see Results and Discussion.

To identify putative phosphorylation targets of SnRK1 which should be downregulated in the *snrk1α1*/*α2* mutant, we searched for overrepresented sequence motifs in the identified phosphopeptides by performing a motif analysis with motif-x (http://motif-x.med.harvard.edu/motif-x.html)^50^. The analysis was done separately to the peptides that were more phosphorylated (at least 50% more abundant and all significantly changed phosphopeptides) in the wild type. There were 3 motifs overrepresented in the wild type samples (Fig. 3a). The highest fold increase was 29.54 for Motif 1 that has a phosphorylated Ser at position 0 as well as at position −2, a positively charged Lys at the −3 position, positively charged Arg or Lys at the −4 position and a hydrophobic amino acid residue, most frequently Leu at −5 and Phe at the +4 position. Also other amino acids known to be at these sites (Leu, Ile, Val, Met, Val, and Phe) are enriched. This motif resembles highly the phosphorylation consensus motif of SnRK1. Motif 2 with the second highest fold increase (6.35) has a Ser at position 0 and a basic Arg at the −3 position. This motif has also enrichment of hydrophobic amino acids at positions −5 and +4. Motif 3 that was overrepresented (fold increase 5.73) is phosphorylated at Ser followed by Pro, which is a low-stringency phosphorylation motif of MAPK^42^ (Supplementary Table S2). When the motif-x search was done for phosphopeptides quantified to be more abundant in the mutant line (Fig. 3b), the low-stringency phosphorylation MAPK motif was also identified with a fold increase of 7.30. The motifs resembling the SnRK1 motif were not identified. Instead, two motifs, Motif 5 (fold increase 29.41) and Motif 6 (fold increase 4.07), having negative charge in the side chain (Asp and Glu) were enriched among peptides that are more phosphorylated in *snrk1α1*/*α2*. Altogether 33 proteins were identified with high stringency SnRK1 motif and 40 proteins with lower stringency (Supplementary Table S2).

### SnRK1-induced systemic shift in the *in vivo* phosphoproteome

**Figure 4:**
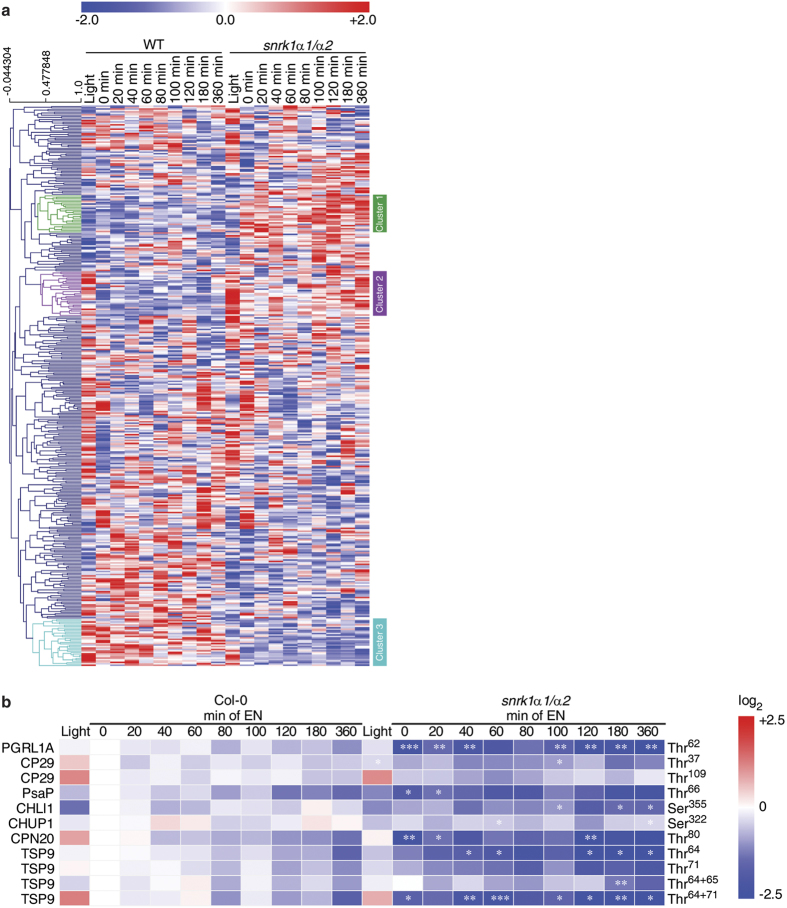
Hierarchical clustering of phosphoproteins. (**a**) Phosphopeptides were standardized to zero-mean-unit-variance (z-scores). In the cluster analysis several groups are altered in their phosphorylation state in the *snrk1α1*/*α2* mutant (e.g. cluster 1, 2 and 3) and they are further discussed in the text. (**b**) Phosphopeptide abundance of chloroplastic proteins. Phosphopeptide abundance of 3 replicates were averaged and normalized to the abundance of the wild type value at the time point 0 min of extended night, the ratios were Log2 transformed. Asterisks indicate significant differences between *snrk1α1*/*α2* and wild type at each time point (*p < 0.05, **p < 0.01, ***p < 0.001).

The phosphopeptides that were significantly different between *snrk1α1*/*α2* and wild type at least at one time point were standardized to zero-mean-unit-variance (referred to as z-scores), to allow the comparison of relative changes of different phosphopeptides, and subjected to hierarchical cluster analysis (HCA) based on Spearman correlation (Fig. 4a). Three clusters clearly differing in the extended night treatment were examined closer. Cluster 1 consisted of phosphopeptides whose phosphorylation status in extended night was higher in the mutant than in the wild type. Many proteins in this cluster do not have known gene ontology annotation or are related to RNA transcription according to the MapMan categorization^51^. Cluster 1 also contained a phosphopeptide from phosphoenolpyruvate carboxylase 1 (PEPC1, AT1G53310) that catalyses irreversible β-carboxylation of phosphoenolpyruvate (PEP) and HCO_3_^−^ to oxaloacetate and Pi, but it also has a role in anaplerotic replenishment of TCA cycle intermediates used for biosynthesis and nitrogen assimilation. While PEPC1 phosphorylation at regulatory Ser^11^ was found to be downregulated in the darkness in wild type, it was constantly high in the mutant and the phosphorylation status even increased in the course of extended night. Like in cluster 1, also cluster 2 contained peptides that were more phosphorylated in the mutant than in the wild type and many of them have no known gene ontology annotation. Again several RNA transcriptional regulators were grouped in this cluster, but also proteins that have a role in protein synthesis. Most significant changes were observed for ribosomal S6 proteins, which are discussed in detail in the following paragraph.

In contrast to clusters 1 and 2, cluster 3 clusters phosphopeptides whose phosphorylation status was upregulated in the wild type and downregulated in the *snrk1α1*/*α2* mutant during darkness (Fig. 4a). Importantly, regulation of these proteins was at the post-translational level, because the protein abundance was similar between wild type and the mutant (Supplementary Fig. S3). In the light, most of the phosphopeptides belonging to this cluster were also found to be regulated differently between mutant and wild type. The cluster 3 grouped several proteins located in the chloroplasts and which have a role in the light reactions: LHCII antenna protein CP29 (also named LHCB4.2, AT3G08940), PGR5-like protein 1A (PGRL1A, AT4G22890), and photosystem I P subunit (PsaP, AT2G46820) (Fig. 4b). CP29 is a phosphorylation target of STN7, a serine/threonine protein kinase which induces state transitions by phosphorylating the light-harvesting complex II outer antennae (LCHII)^52^. Further, CP29 was shown to be phosphorylated on multiple Thr residues^53^. In the present study, Thr^37^ and Thr^109^ were found to be more phosphorylated in the light than in the dark and the abundance was higher in the wild type compared to *snrk1α1*/*α2* mutant independent from the light conditions. Another darkness-induced effect was observed for the phosphorylation of PGRL1A, a phosphorylation target of STN8, which is a ferredoxin-plastoquinone reductase^54^ playing a central role in antimycin A (AA) sensitive cyclic electron transfer (CET). In the light, phosphorylation of PGRL1A Thr^62^ was found to be at the same level in *snrk1α1*/*α2* and the wild type, while in darkness the phosphorylation level decreased considerably in *snrk1α1*/*α2*. Another plastidial protein which was differently phosphorylated in wild type and *snrk1α1*/*α2* was PsaP, a subunit of PSI^55^, which was also described to play a role in thylakoid grana architecture^56^. While PsaP can be hyperphosphorylated, the regulation of phosphorylation does not appear to be light dependent but mediated by the kinases STN7 or STN8^57^. Our data implied that the phosphorylation of PsaP Thr^65/66^ depends on SnRK1 activity, but it remains to be explained whether and how SnRK1 mediated processes are transferred into the chloroplast or play another role in protein translocation or degradation. The presented experimental data showed that phosphorylation of these plastidial proteins was not differing between wild type and *snrk1α1*/*α2* in the light time point, but in the extended night lack of SnRK1 downregulates the phosphorylation. Another dominant effect which was due to both the genotype and the condition was observed for the chloroplastic 20 kDa chaperonin CPN20 (AT5G20720), which was found to be phosphorylated much stronger at Thr^80^ in the wild type. This phosphorylation pattern could already be observed in the light, but in darkness the relative phosphorylation status of CPN20 at Thr^80^ was found to be significantly lower in the mutant. In addition to these chloroplastic proteins, also phosphorylation of CHLOROPLAST UNUSUAL POSITIONING 1 (CHUP1, AT3G25690) and thylakoid soluble phosphoprotein of 9 kDa (TSP9; AT3G47070) were affected by SnRK1 (Fig. 4b). CHUP1 is a protein that has a role in chloroplast movement^58^,^59^. It is essential for preventing photo damage under high light conditions but also under weak light CHUP1 is needed for optimal positioning of chloroplasts for light harvesting and photosynthesis^59^. TSP9 is target of STN7^60^ and it is involved in regulation of light harvesting by interacting with LHCII and peripheries of both PSII and PSI^61^.

### Phosphorylation of ribosomal S6 proteins and eukaryotic translation factors eIF5A2 and eIF5A3 is upregulated in *snrk1α* mutants

**Figure 5:**
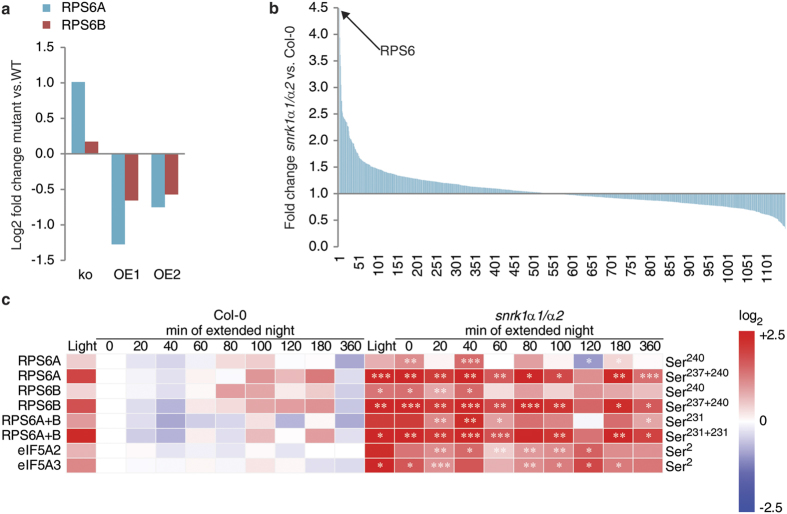
Regulation of RPS6 phosphorylation. (**a**) RPS6 subunit phosphorylation at position Ser^240^ was downregulated in SnRK1α1 overexpressor lines (OE1 and OE2) and upregulated in *snrk1*α*1* mutant (ko). Relative phosphopeptide abundances were normalized to the corresponding wild type (ko to Col-0 and OEs to Ler). (**b**) Fold changes of all identified and quantified phosphopeptides. The highest fold change in the *snrk1α1*/*α2* mutant of ~4.5 fold change was found for the TOR-signalling target RPS6 as indicated by the arrow. For calculation of the fold change all values from all time points in each genotype were summed up and then the ratio was build. (**c**) A detailed analysis of dynamic phosphorylation of RPS6 and translation initiation factors eIf5A2 and eIf5A3 showed an upregulation in the *snrk1α1*/*α2* mutant line in extended night. Phosphopeptide abundances of 3 replicates were averaged and normalized to the abundance of the wild type value at the time point 0 min of extended night, the ratios were Log2 transformed. Asterisks indicate significant differences between *snrk1α1*/*α2* and wild type at each time point (*p < 0.05, **p < 0.01, ***p < 0.001).

RPS6 proteins are the substrates for the S6K protein kinase and represent typical TOR signalling targets in animal and plant cells^22^,^23^,^32^,^62^,^63^. In Arabidopsis, TOR and RPS6 are essential for the same cellular processes and have overlapping functions, like necessity for embryo viability, growth and life span behaviour^26^. However, the direct link to SnRK1 signalling was so far only speculative. We found that the phosphorylation of RPS6 proteins was changed in plants with altered SnRK1 activity. In Arabidopsis, there are two RPS6 isoforms, RPS6A (AT4G31700) and RPS6B (AT5G10360). RPS6A was antagonistically phosphorylated at Ser^240^ in *snrk1α1* and overexpressor lines compared to the wild type with increased phosphorylation in the absence of SnRK1 and decreased signal in overexpressing lines (Fig. 5a). In the time course experiment, where we utilized a *snrk1α1*/*α2* double mutant, we found that from the 1150 quantified phosphoproteins the phosphorylation of RPS6 protein showed the strongest total fold change in extended night (Fig. 5b). The phosphorylation of Ser^240^ in RPS6A and RPS6B was observed to be constitutively higher across the extended night treatment (Fig. 5c). Further, other C-terminal Ser residues were detected, Ser^237^, Ser^231^, and Ser^229^, and they also were more phosphorylated in *snrk1α1*/*α2* plants than in the wild type. In the light conditions, phosphorylation of RPS6A Ser^240^ did not differ between genotypes, whereas Ser^240^ in RPS6B and Ser^229^ in both RPS6 isoforms were found to be twofold higher in the mutant than in the wild type. At the end of the night, the phosphorylation status of all the mentioned RPS6-related peptides was increased >2-fold in the *snrk1α1*/*α2* compared to wild type and peaked after 40 min of extended night with a >3-fold difference. Subsequently, this ratio decreased again during the extended night time course, finally resulting in an about 2-fold higher phosphorylation level in the mutant.

Comparing the phosphorylation abundance of doubly phosphorylated C-terminal peptides in RPS6A and RPS6B, the difference between the wild type and *snrk1α1*/*α2* mutant was found to be even higher than it was revealed by the comparison of singly phosphorylated peptides (Fig. 5c). Already in the light conditions, the ratio of *snrk1α1*/*α2* versus wild type was almost 4-fold and peaking at 40 min of extended night with a ratio >7. In agreement with the observation for singly phosphorylated peptides, also the ratio of doubly phosphorylated peptides decreased during extended night reaching a level of 3-fold in case of RPS6A and 4-fold in case of RPS6B after 360 min of extended night. Importantly, the total protein level of RPS6 was not higher in the mutant than in the wild type, on the contrary the level of RPS6B (RPS6A could not be quantified) was observed to be even lower in the mutant than in the wild type (Supplementary Fig. S4a and Table S3). Moreover, the abundance was similar in the light and in the end of the night so the regulation of RPS6 seems to happen at the post-translational level and not by adjusting the protein amount.

The observed differences in RPS6 phosphorylation indicated an effect of the *snrk1α1*/*α2* mutation on the regulation of protein synthesis (Fig. 5c). Another highly conserved and essential protein involved in translation is eukaryotic translation initiation factor 5A (eIF5A). Its role in translation has been studied in many organisms and its expression is induced in proliferating cells, while it is repressed under carbohydrate starvation^64^,^65^. Although eIF5A is conserved across eukaryotes, the N-terminus of the protein was shown to differ when comparing plants and mammals. Plants and yeast contain a Ser residue at position 2. In the present study, this Ser^2^ was found to be more phosphorylated in eIF5A2 (AT1G26630) and eIF5A3 (AT1G69410) of *snrk1α1*/*α2* when compared to the wild type (Fig. 5c). Further, the phosphorylation of Ser^2^ in eIF5A2 and eIF5A3 was observed to be higher in the light conditions, to decrease in the dark and to stay low during the whole extended night time course. This light-dependent regulation could also be observed in *snrk1α1*/*α2*, but the phosphorylation level was constantly higher than in the wild type. The total abundance of eIF5A2 is unchanged during the course of extended night and also between wild type and mutant (Supplementary Fig. S4b and Table S3), while the total amount of eIF5A3 was not affected by light conditions and energy depletion in extended night but its level was constantly higher in the *snrk1α1*/*α2* mutant than in wild type (Supplementary Fig. S4c and Table S3).

### SnRK1α1 and RAPTOR1B co-localize and interact in the cytosol and SnRK1 phosphorylates RAPTOR in an *in vitro* kinase assay

**Figure 6:**
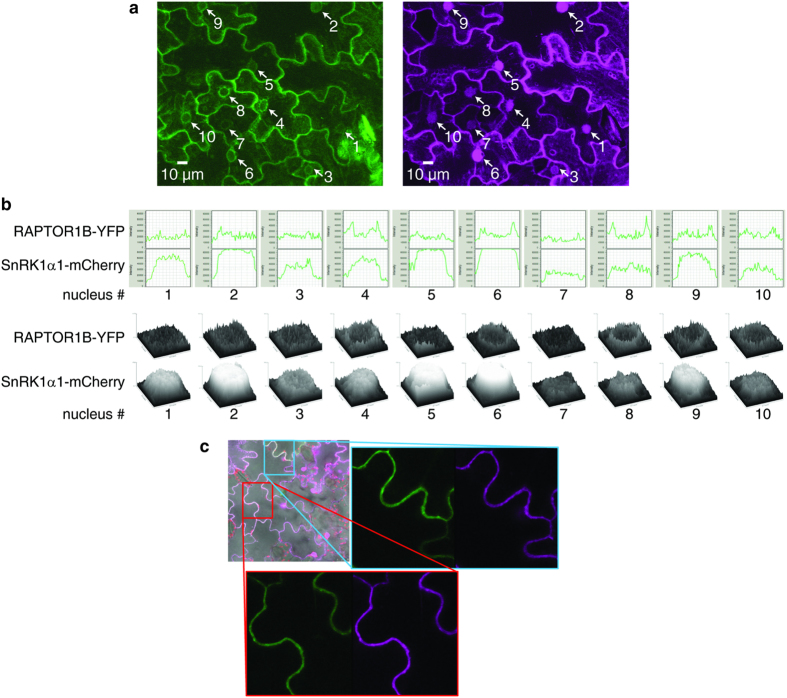
Subcellular localization of RAPTOR1B and SnRK1α1. (**a**) Cytoplasmic localization of RAPTOR1B; z-stack maximum intensity projections of the signals originating from RAPTOR1B-YFP (green) and SnRK1α1-mCherry (magenta) transiently expressed in tobacco epidermal leaves. Stack dimensions x: 246.03 μm, y: 246.03 μm, z: 23.12 μm (40 frames). Nuclei are marked by arrows. (**b**) Diameter intensity blots over the nuclear area of maximum intensity projections from (**a**). Each blot is numbered and refers to the nuclei in (**a**). Concave curve shapes indicate localization of SnRK1α1-mCherry in the nucleus whereas convex curve shapes around the nuclear area indicate only cytoplasmic localization of RAPTOR1B-YFP. (**c**) Surface intensity blots of nuclear areas indicated in (**a**). (**d**) Co-localization of RAPTOR1B and SnRK1α1 in the cytoplasm. Overlay image of bright-field and signals from plastids (autofluorescence, red), SnRK1α1-mCherry (magenta) and RAPTOR1B-YFP (green) both transiently expressed in tobacco leaf epidermal cells. Rectangles indicate magnified areas of the image. Each magnified area contains separated RAPTOR1B-YFP and SnRK1α1-mCherry signals from the same image position.

In animal cells AMPK is known to regulate mTORC1 (mammalian target of rapamycin complex 1) in energy stress by phosphorylating one of the complex members, namely RAPTOR^35^. Interaction and crosstalk between SnRK1 and TOR mediated pathways have been speculated to exist also in plants. Our phosphoproteomics experiments, examining the role of SnRK1 in conditions where energy is scarce, revealed this potential antagonistic crosstalk of SnRK1 and TOR pathways. Therefore, we transiently transformed *Nicotiana tabacum* leaves with SnRK1α1 and RAPTOR1B tagged with mCherry and YFP, respectively, to study whether the SnRK1α1 and RAPTOR have the same subcellular localization. While SnRK1α1 was localized both in the cytosol and in the nucleus, RAPTOR1B is localized only in the cytosol (Fig. 6). Also mammalian RAPTOR localizes exclusively in the cytosol^66^.

**Figure 7:**
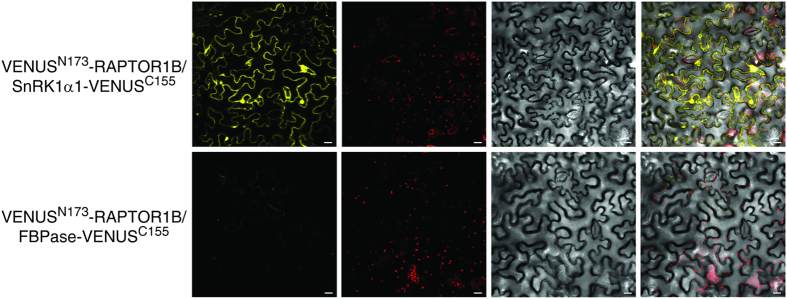
SnRK1α1/RAPTOR1B *in vivo* interaction in planta by the BiFC assay. Leaves of *N. benthamiana* were transiently transformed with mixtures of protein pairs using Agrobacterium-infiltration. Bait and prey proteins were fused to the N- and C-terminal part of Venus, respectively. Fluorescence and localization were observed by confocal laser scanning microscopy 48 h after infiltration. Bar represents 20 μm. The cytosolic FBPase fused to the C terminus of Venus expressed in combination with RAPTOR1b-VenusN^173^ induces no fluorescence signal and serves as a negative control (lower panel). All pictures were recorded with the same microscope settings. The experiment was repeated three times with similar results.

**Figure 8:**
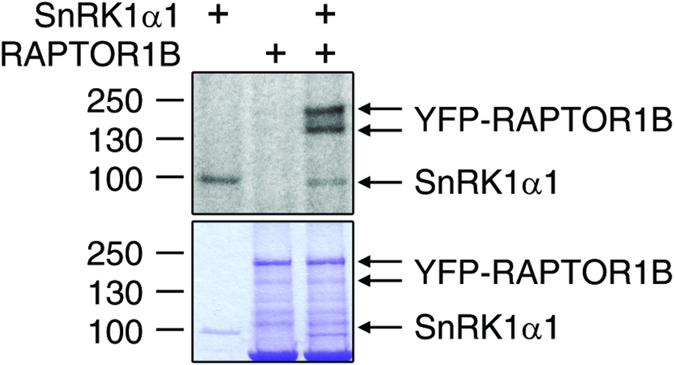
SnRK1 phosphorylates RAPTOR1B *in vitro*. The recombinant SnRK1α1 kinase phosphorylated RAPTOR1B in *in vitro* kinase assay. Autoradiography, which shows the incorporation of γ-^32^P into the RAPTOR1B, on top and Coomassie brilliant blue staining of proteins on bottom.

We also performed bimolecular fluorescence complementation (BiFC) assays to investigate whether SnRK1α1 and RAPTOR1B are interacting *in vivo*. To this end, leaves of *N. benthamiana* were transiently co-transformed with plasmids containing RAPTOR1B plus the N-terminal part of VENUS (VENUS^N173^) and SnRK1α1 plus the C-terminal part of VENUS (VENUS^C155^). As negative control we used the cytosolic fructose-1,6-bisphosphatase (FBPase) fused to the VENUS^C155^ in combination with VENUS^N173^-RAPTOR1B. As shown in Fig. 7 SnRK1α1-Venus^C155^ and Venus^N173^-RAPTOR1B induced a fluorescence signal indicative for an interaction of both proteins in the cytosol while the control combination yielded no signal. To test weather SnRK1α1 can phosphorylate RAPTOR1B, we performed an *in vitro* kinase assay. The phosphorylation of RAPTOR1B by SnRK1α1 is demonstrated in Fig. 8. In summary, the co-localisation, *in vivo* interaction and phosphorylation of RAPTOR1B by SnRK1α1 suggest a mechanism of SnRK1 control over TOR signalling by phosphorylation of RAPTOR.

### Changes in metabolite levels reveal a significant impact of SnRK1 on the coordination of primary metabolism

**Figure 9:**
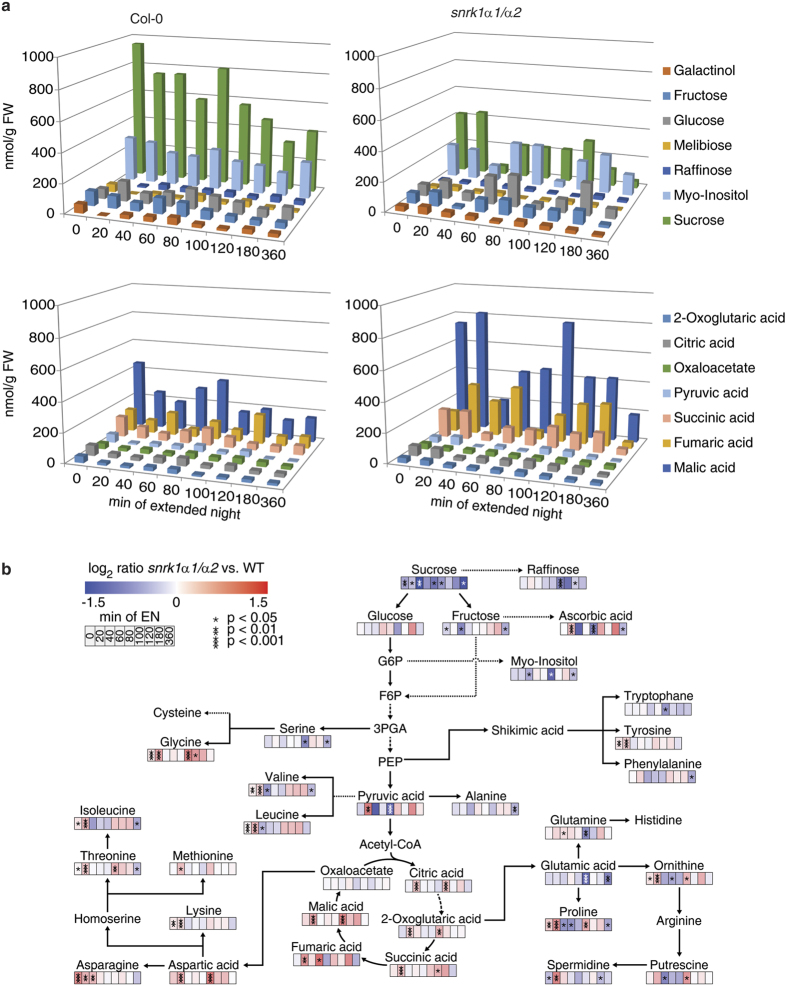
Changes in metabolite concentrations in wild type and *snrk1α1*/*α2* mutant plants under extended night conditions. (**a**) Sugars and TCA cycle intermediates show an inverse behaviour in the wild type and *snrk1α1*/*α2* mutant (n = 5). Sugars are lower and TCA cycle intermediates are higher concentrated in the mutant compared to the wild type. This indicates an impaired crosstalk of cytosolic and mitochondrial metabolism in the mutants. (**b**) Ratios of significantly changed metabolite levels during an extended night time course in the *snrk1α1*/*α2* mutant versus wild type. According to the colour bar, colours indicate the log2 value of the ratios which were built from absolute mean levels determined for *snrk1α1*/*α2* and wild type (n = 5). Ratios were built according to the formula *snrk1α1α2*/wild type. Asterisks indicate significant differences between metabolite levels (*p < 0.05, **p < 0.01, ***p < 0.001). A detailed overview of absolute metabolite levels and standard deviations is provided in the Supplementary Table S4.

The analysis of the primary metabolome during an exposure to extended night revealed significant differences between wild type and *snrk1α1*/*α2* plants in the accumulation of sugars, sugar-alcohols, organic acids, amino acids and polyamines (Fig. 9 and Supplementary Table S4). Significant changes between both genotypes (t-test, p < 0.05) in the accumulation of these compounds could be observed throughout the whole extended night period, although the differences were particularly high at 20 min and 100 min of the extended night treatment. Whereas sucrose was found to be constantly lower during the whole time course in *snrk1α1*/*α2*, almost all TCA intermediates were significantly increased after 20 min and, in part, also after 100 min (Fig. 9a). Finally, after 360 min of extended night, the levels of sucrose, myo-inositol and fructose were significantly lower in the mutant while there was no significant effect visible in TCA intermediates. At this time point, about half of the analysed amino acids (8 out of 18) were significantly lower in the mutant. The levels of polyamines were also higher in the mutant (Fig. 9b).

### Covariance-based predictions of metabolic regulation reveal an early and a late phase of metabolic reprogramming during the extended night time course

**Figure 10:**
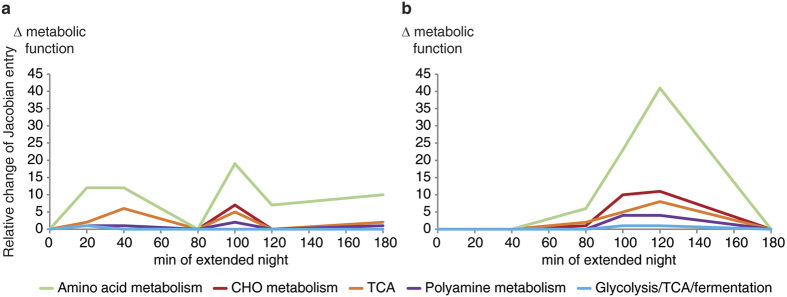
Changes in metabolic functions of wild type and *snrk1α1*/*α2* during the time course measurement in extended night. The metabolic functions for wild type (**a**) and *snrk1α1*/*α2* (**b**) were derived by the inverse calculation of the biochemical Jacobian according to Nägele *et al.*^92^. Individual entries of the Jacobian represent changes in reaction rates in response to metabolite concentration changes. Individual Jacobian entries were summed up into metabolic functions and plotted over the time course. A significant difference is the missing early response in the *snrk1α1*/*α2* mutant in the beginning of the extended night phase. A detailed overview of characteristic components of the metabolic functions and their related biochemical pathways is provided in the Supplementary Table S5.

While mean values of metabolite levels provide information about a metabolic steady state of a genotype at a certain time point, the linkage of the metabolite covariance information with biochemical pathways can provide helpful information about metabolic regulation (for more information see refs 67,68). Plotting the changes of the metabolic functions derived by the inverse calculation of the biochemical Jacobian from metabolomics covariance data (for more information see ref. 67) revealed a significantly different pattern of wild type and the *snrk1α1*/*α2* mutant (Fig. 10). While in wild type the most dominant effect was found to be the separation of early (20–40 min) and late (100–180 min) time points (Fig. 9a), this was not observed in the mutant (Fig. 10b). Here, separation between late time points of extended night (80–120 min) was the strongest effect while the separation of early time points was not present.

Analysis of the cluster components revealed that these early events, i.e. between 20 and 40 min of extended night, were biochemically related to the reprogramming of amino acid metabolism, sugar metabolism, the TCA cycle as well as glycolysis (Supplementary Table S5). In the mutant, this reprogramming was found to take place about 60 min later, i.e. after 100–120 min of extended night exposure. Furthermore, at these late time points, both the wild type and the mutant showed a strong reprogramming of amino acid and polyamine metabolism (Supplementary Table S5).

### Dynamics in the proteome revealed an unbalanced C/N metabolism in *snrk1α1*/*α2*

Relative protein abundances revealed a significant perturbation of the C/N-metabolism in *snrk1α1*/*α2* (Supplementary Table S6). This included proteins which were grouped in the functional categories of cytosolic glycolysis, mitochondrial electron transport, amino acid synthesis and degradation, TCA cycle and redox-related components according to the MapMan ontology^51^. Strongest effects in *snrk1α1*/*α2* when compared to wild type were observed during the very early phase of the extended night, i.e. after 20 min. Here, particularly glycolysis and redox metabolism were strongly deregulated in the mutant. Glycolysis connects sugar metabolism with the pyruvic acid pool and the TCA cycle intermediates and, hence, the finding of a perturbed glycolytic pathway after 20 min of extended night might explain why levels of sugars, organic and amino acids in *snrk1α1*/*α2* were found to be strongly deviating from the wild type levels. In addition, also protein components of the TCA cycle and the mitochondrial electron transport chain (mETC) were found to be significantly affected in the *snrk1α1*/*α2* mutant. In particular, during the first 180 min of extended night a linear increase of proteins related to the mETC could be observed for the wild type (R^2^ = 0.94) while the response in *snrk1α1*/*α2* was found to be less linear (R^2^ = 0.49).

In agreement with the observation that protein components of cellular redox metabolism behaved differently in the very early phase of extended night in wild type and *snrk1α1*/*α2*, also abundances of related phosphorylated peptides indicated a contrasting reprogramming in both genotypes. In particular, phosphopeptide abundance of thioredoxin H-type 9 (ATH9, AT3G08710) was slightly elevated in *snrk1α1*/*α2* during extended night, indicating an interaction of SnRK1 signalling with REDOX regulation.

## Discussion

**Figure 11:**
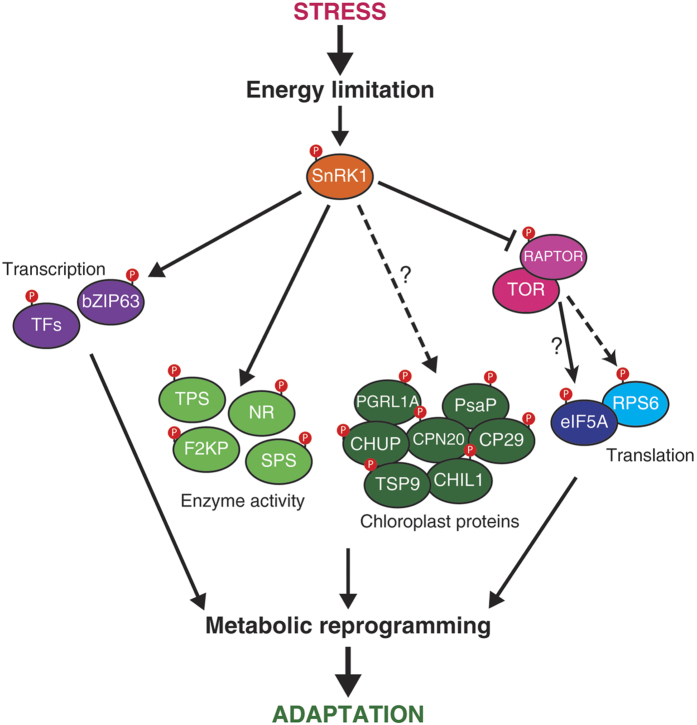
Overview of the processes controlled by SnRK1 in plants revealed by dynamic phosphoproteomics in the *snrk1α1*/*α2* mutant versus the wild type plants in light and extended night conditions. The most abundant effect in the *snrk1α1*/*α2* mutant in a day-night time course inducing the LES was a hyperphosphorylation of RPS6 which indicated an activated TOR signalling pathway. Typically this pathway is shut down during LES as demonstrated in the wild type phosphoproteome during extended night. A proposed model for SnRK1 regulation of TOR signalling is via interaction and phosphorylation of RAPTOR. Further unexpected effects were observed for chloroplastic phosphoproteins and the crosstalk of cytosol and mitochondria. For detailed discussion see Results and Discussion.

Protein phosphorylation is an important post-translational modification that controls e.g. protein activity, interaction with other proteins, protein turnover, and their subcellular localization. Phosphoproteomics technology allows the examination of thousands of phosphorylation events *in vivo*. Heterotrimeric SnRK1 kinase is activated in scarce energy conditions such as in extended darkness. This is the first phosphoproteomics report of a SnRK1 alpha subunit double knockdown Arabidopsis mutant (snrk1α1/α2 also known as AKIN10/11) in extended darkness. An overview of the changes in the *in vivo* phosphoproteome is shown in Fig. 11.

The processes of ribosome biogenesis, protein translation, and growth significantly affect cellular energy homeostasis, and, therefore, adjusting the translational activity to energy availability is crucial for all organisms. RPS6 proteins are one of the central targets of the TOR signalling pathway via S6K in animal and plant cells^22^,^23^,^32^,^62^,^63^ and it is commonly accepted that phosphorylation of RPS6 regulates translational capacity. In Arabidopsis, TOR and RPS6 are essential for the same cellular processes, like necessity for embryo viability, growth and life span behaviour^26^. The normal response to energy deprivation during extended night is an inactivation of TOR and reduction of protein synthesis and growth. We found that the phosphorylation of RPS6 proteins was changed in plants with altered SnRK1 activity. In the *snrk1α1*/*α2* mutant this inactivation was lost and it led to constitutively high phosphorylation of RPS6 (Fig. 5). At the same time, SnRK1 overexpressor lines showed reduced phosphorylation of the RPS6 subunits (Fig. 5a). Thus, we suggest that SnRK1 activity is required for regulation of TOR and protein synthesis under energy deprivation.

Based on studies in mammalian cells, it is known that mTORC1 (mammalian target of rapamycin complex 1) is inhibited by energy depriving conditions and by AMPK-driven phosphorylation of one of the complex members, namely RAPTOR^35^. Interaction and crosstalk between SnRK1- and TOR-mediated pathways have been speculated to exist also in plants. In human cells, RAPTOR Ser^792^ and Ser^722^ are known AMPK phosphorylation sites. Gwinn *et al*. predicted AMPK/SnRK1 sites also in Arabidopsis RAPTOR proteins, Ser^786^ and Ser^768^ in RAPTOR1B and RAPTOR1A, respectively^35^. However, only the Ser^792^ in human RAPTOR is conserved also in Arabidopsis whereas Ser^722^ is not^35^. The Arabidopsis Ser^786^ is not in an ideal tryptic peptide because the peptide is 43 amino acids long, and most probably therefore it was not detected in this experiment and also not in any other phosphoproteomics experiments included in the PhosPhAt database^47^. For this reason, we transiently expressed SnRK1α1 and RAPTOR1B in tobacco leaves to study whether they are co-localizing and interacting in the cells. While RAPTOR1B was located only in the cytosol, SnRK1α1 localized also in the nucleus (Fig. 6). Similar cytosolic localization of RAPTOR was also shown in mammalian cells^66^. Furthermore, the BiFC assay with VENUS indicated interaction between SnRK1α1 and RAPTOR1B *in vivo* (Fig. 7). To test wether Arabidopsis SnRK1α1 can phosphorylate RAPTOR1B, as AMPK phosphorylates RAPTOR in mammals, we did an *in vitro* kinase assay. This assay demonstrated that SnRK1α1 is able to phosphorylate RAPTOR1B at least *in vitro* (Fig. 8). Because an interaction of RAPTOR and TOR was already demonstrated in Arabidopsis ^32^we provide evidence that RAPTOR phosphorylation by SnRK1 is a potential mechanism of SnRK1 control of TOR signalling in plants. In future studies, we will target this potential mechanism for the control of SnRK1 on TOR signalling.

The eukaryotic translation factor eIF5A (eIF5A1, eIF5A2, and eIF5A3 in Arabidopsis), appears to be highly conserved in all eukaryotes^69^. Interestingly, the most divergent part between plants and mammals is the N-terminus, which is conserved between plants and parasites^70^,^71^. The N-terminus of plant eIF5As contains a Ser residue at position 2 which we found to be more phosphorylated in *snrk1α1*/*α2* mutant than in Col-0 wild type (Fig. 5). Additionally, our data indicated that the phosphorylation of eIF5A2 and 3 is light regulated. In agreement with our data, Boex-Fontvieille *et al*. showed that phosphorylation of Ser^2^ residues in eIF5A2 and eIF5A3 is induced by light but does not depend on photosynthetic activity^72^. Further evidence that eIF5A is involved in energy deprivation comes from other organisms. The transcript levels of rice (*Oryza sativa*) OseIF5A1 and OseIF5A1 in suspension cell culture are strongly affected by carbohydrate starvation and the amount of mRNA is reduced when sugars are not available^65^. In exponentially growing protozoan parasite *Trypanosoma cruzi* TceIF5A is highly phosphorylated at the same N-terminal Ser^2^ as in Arabidopsis^64^, indicating that the phosphorylation has a significant role regulating protein synthesis and growth. Moreover, depletion of eIF5A in yeast (*Saccharomyces cerevisiae*) rapidly affects the protein translation rate impairing it by 2–3-fold^73^. Further functions of phosphorylation of eIF5A might comprise transport processes of mRNA from the nucleus to the ribosome, thus initiating programmed cell death (PCD)^74^,^75^.

The central role of SnRK1 in regulating and coordinating the energy homeostasis of plants has been shown in several recent studies. While it can be assumed that SnRK1 activity affects multiple cellular processes in an indirect manner by activating or inhibiting signalling cascades, already the number of direct interactions with plant primary metabolism suggests its role as a central regulator. Prominent examples of SnRK1 substrates in primary metabolism are NR, TPS, and SPS, which all are inactivated by phosphorylation^76^. In previous studies these proteins were shown to be phosphorylated by SnRK1 *in vitro* and here we showed that they represent also *in vivo* targets of SnRK1 (Fig. 2). Moreover, the metabolic interactions, i.e. metabolic inhibition, of primary metabolism indicate a complex interplay between SnRK1 activity, metabolite abundances and biochemical pathway regulation^11^,^12^. Our results provide evidence for a sequence of steps of metabolic reprogramming, thereby adjusting metabolism to LES. Based on the changes in metabolite levels and covariance information, the presented data indicate, that plants have short-term and long-term responses in primary metabolism when exposed to energy depleting conditions for 360 min. Both on the level of the metabolome and the proteome the presented findings point to a fast reprogramming of glycolysis, TCA cycle, and mitochondrial respiration in the first 20 min. A metabolic consequence in the *snrk1α1*/*α2* mutant became visible after 20 and 100 min of extended night period when levels of TCA cycle intermediates and amino acids increased significantly compared to the wild type while sucrose level decreased (Fig. 9). In addition, results of the covariance-based modelling approach indicated that an early step of metabolic reprogramming, which only occurred in wild type, affected sucrose and TCA metabolism (Fig. 10 and Supplementary Table S5). This observation leads to the hypothesis that SnRK1-activity reprograms the flux of carbon equivalents in direction of the TCA cycle as an immediate reaction towards energy depletion. A possible regulatory mechanism which might be involved in this fast response is the SnRK1-driven phosphorylation and inactivation of F2KP. In wild type, this might lead to a stronger accumulation of fructose-2,6-bisphosphate which inhibits the cytosolic fructose-1,6-bisphosphatase (FBPase) and by this affects sucrose synthesis^77^. Evidence for this hypothesis is provided by the observation that the relative level of a phosphopeptide belonging to F2KP was found to be higher in wild type compared to *snrk1α1*/*α2*. This effect was found to be most pronounced during the early phase of extended night exposure (Supplementary Fig. S5).

In addition to the observation that SnRK1 seems to be involved in a fast reprogramming of carbon partitioning between sucrose synthesis, glycolysis and respiration, the reprogramming to a later time point, i.e. after 120–360 min of extended night, also showed the expected pattern on the level of glycolytic protein abundance, providing evidence for a SnRK1-dependent activation of catabolic pathways^78^,^79^. This picture of a short-term and long-term response emphasizes once more the multifaceted impact of SnRK1 activity on energy metabolism, comprising various levels of molecular organization^1^,^16^,^21^.

## Materials and Methods

### Plant material and growth conditions

For the first experiment, a SnRK1α1 T-DNA knockout mutant *snrk1*α*1 (snrk1α1-3*)^38^, *35S::SnRK1*α*1* overexpressor lines 1 and 2 (SnRK1α1 OE1 and SnRK1α1 OE2; Baena-Gonzalez *et al.*,^14^) and their corresponding Columbia (Col-0) and Landsberg erecta (Ler) wild type backgrounds, respectively, were used. Plants were grown in soil in a 12/12 light/dark cycle and irradiance was set to 100 μmol m^−2^s^−1^. Five week old mature leaf rosettes were exposed to extended night for 120 min and harvested under green light to prevent reversion of the extended night effect by activation of photosynthesis. For the second time course experiment, a *snrk1α1-3* expressing a β-estradiol-inducible amiRNA against *SnRK1α2 (snrk1α1*/*α2*) was used. Plants were grown in a 12/12 light/dark cycle with irradiance of 100 μmol m^−2^ s^−1^. After 4 weeks they were sprayed with 10 μM β-estradiol, 0.1% DMSO, 0.005% Silwet for 6 days. On day 7 plants were exposed to extended night treatment and harvested at 0, 20 40, 60, 80, 100, 120, 180, and 360 min after start of the treatment. Also samples in the middle of the day (6 h light) were collected. At this time, plant leaf rosettes were fully grown but not yet flowering.

### Western blotting

Protein for Western blot analysis was extracted with buffer containing 175 mM Tris-HCl (pH 8.8), 5% SDS, 15% glycerol and 300 mM DTT. After vortexing, samples were centrifuged with 21 000 g and the protein was precipitated from the supernatant with four volumes of ice-cold acetone. Protein precipitates were collected and resuspended in Laemmli buffer^80^. Protein was separated with SDS-PAGE and blotted on a PVDF membrane with semi-dry transfer. Primary antibodies used were anti-AKIN10 (AS10919 Agrisera) and anti-phospho-AMPK (Thr172) (#2531 Cell Signaling). ECL (enhanced chemiluminescence) detection was done as described in manufacturer’s protocol.

### Protein extraction and tandem MOAC

Leaves of *snrk1*α*1, 35S:SnRK1*α*1* overexpressor lines 1 and 2 and their corresponding Columbia (Col-0) and Landsberg erecta (Ler) wild type backgrounds as well as from *snrk1α1*/*α2* mutant were extracted with TCA precipitation and phenol extraction^81^. Subsequently, phosphoproteins and phosphopeptides were enriched by tandem or single MOAC approach as previously described^40^,^41^,^42^,^44^. To retain hydrophilic phosphopeptides after trypsin digestion, peptides were desalted with C18 and carbon graphite SPE strategy^45^.

### LC-MS/MS for phosphoproteomics

Phosphopeptide pellet was resolved in 8 μL of 5% (v/v) acetonitril (ACN), 0.5% (v/v) formic acid (FA) and 5 μL were loaded on C18 LC column. The samples of the first experiment were measured on monolithic Chromolith C18 CapRod column with a length of 15 cm and an inner diameter (ID) of 0.1 mm (Merck, Germany). Peptides were eluted using water/ACN gradient from 2% to 35% mobile phase B with 500 nL/min flow rate generated with nanoLC (Eksigent) within 150 min linear gradient. Mobile phase A: 0.1% (v/v) FA, mobile phase B: 0.1% (v/v) FA in 90% ACN. In the second experiment UltiMate 3000 RSLCnano system with EASY-Spray PepMap C18 column with a length of 50 cm and ID of 0.075 mm with 2 μm particles (Thermo Scientific) was used. Same mobile phases were used and the gradient was 150 min from 2% to 40% mobile phase B with 300 nL/min flow rate.

The samples were measured with an LTQ-Orbitrap mass spectrometer (Thermo Electron). Orbitrap mass analyser settings: ion transfer capillary temperature 180 °C, full scan range 350–1800 m/z, resolution 30 000. Each FTMS full scan was followed by up to five data dependent (DDA) CID tandem mass spectra (MS/MS spectra) in the linear triple quadrupole (LTQ) mass analyser. Dynamic exclusion was enabled with list size of 500 with exclusion width ±10 ppm for 60 s. Charge state screening was enabled and unassigned and +1 charged ions were excluded from MS/MS acquisition. For injection, control automatic gain control (AGC) for full scan acquisition in the Orbitrap was set to 5 × 10^5^, the maximum injection time (max IT) was set to 500 ms. Orbitrap online calibration using internal lock mass calibration on m/z 371.10123 from polydimethylcyclosiloxane was used. Multistage activation/pseudoMS3 was enabled with a neutral loss mass list of 24.49, 32.66, 48.999, 97.97, 195.94, and 293.91 Da for neutral loss of phosphoric acid [H3PO4] at charge states +1, +2, +3 and +4, respectively, 195.94, 97.97 and 48.999 Da for neutral loss of two phosphoric acids [2H3PO4] at charge states +1, +2 and +4, respectively, and 293.91 Da for neutral loss of three phosphoric acids [3H3PO4] at charge state +1. The AGC for MS/MS acquisition in the LTQ was set to 1 × 10^4^, the max IT was set to 100 ms.

ProteomeDiscoverer 1.3 (Thermo), SEQUEST search algorithm^82^ and PhosphoRS^83^ were used for peptide identification phosphorylation site mapping of the first experiment. Precursor mass tolerance was set to 5 ppm and fragment ion tolerance to 0.8 Da and maximum 2 missed cleavages were allowed. Phosphorylation of serine, threonine and tyrosine (+79.966 Da) and oxidation of methionine (+15.995 Da) were set as dynamic modifications. Search was done against Arabidopsis TAIR10 protein database where common contaminants were added. Search results were filtered and only high confidence peptides with XCorr 2.0 or more were used for further analysis. Relative quantification was done with ProtMAX 2012 tool version 2.14^84^ and DanteR software^85^. For that, the Thermo RAW files were converted to mzXML format with MassMatrix MM File Conversion Tool and quantification was done in ProtMAX by summing parent ion intensities. The quantified phosphopeptides were Log10 transformed, EigenMS normalized and missing values were imputed using k-nearest neighbour (KNN) method.

The data from experiment 2 were analysed with MaxQuant 1.4 and Andromeda search algorithm^86^,^87^. Mass tolerance for precursor was set to 5 ppm and for fragment masses to 0.8 Da. The maximum FDR was set to 1%. Three missed cleavages were allowed because the phosphorylation near to a tryptic site can hinder digestion. Dynamic modifications allowed were phosphorylation (STY), methionine oxidation and protein N-terminal acetylation. Further data analysis, normalization and transformation were done with Perseus 1.5 and Microsoft Excel. Phosphopeptides that are discussed in detail were quantified by using MS1 filtered extracted ion chromatograms in Skyline^88^.

### RAPTOR1B localization and SnRK1 and RAPTOR1B BiFC assay

RAPTOR1B (AT3G08850) cDNA was PCR amplified from *A. thaliana* reverse transcribed total cDNA (from rosette leaf) and cloned into a modified pBIN19 vector^89^ resulting in a RAPTOR1B-YFP fusion ORF under the control of a cauliflower mosaic virus (CaMV) 35S promoter (see attached vector map, Supplementary File S1). A SnRK1α1-mCherry plant expression plasmid was already available in the laboratory (see attached vector map, Supplementary File S2). Subsequently, 1 μL of plasmid DNA [1 μg/μL] was mixed with 50 μL of electro competent Agrobacteria (AGL1 [AGL0 (C58 pTiBo542) recA::bla, T-region deleted Mop(+) Cb(R)]) in a clean and sterile electroporation cuvette on ice. Electroporation was done with a BioRad Gene PulserTM under following conditions: 200 Ohm resistance, 1.4 kV voltages and 25 μF capacitance. Immediately, after the electric pulse cells were incubated in 1 mL LB and incubated at 30 °C and vigorous shaking for 1 h. Cells were then transferred onto appropriate selective LB agar plates and incubated at 30 °C. For tobacco leaf infiltration the plasmid containing agrobacteria were incubated over night at 25 °C constantly shaking. Next, the agrobacteria suspension was diluted to an OD600 of 0.3 and left shaking for 5 h at 25 °C followed by incubation with 150 μM acetosyringon at 25 °C for another 2 h. Then agrobacteria were resuspended in 5% sucrose solution and OD600 was adjusted to 2. For co-expression equal volumes of both agrobacteria suspensions (RAPTOR1B and SnRK1α1) were mixed and infiltrated into 5 weeks old *N. tabacum* (cv. petit Havana SR1) leaves. After 36 h infiltrated tobacco leaves were subjected to microscopy. Microscopy was performed on a Leica TCS SP5 confocal platform. General microscope settings: objective - HCX PL APO CS 63.0×1.30 GLYC 21 °C UV; emission light source - white light laser Ex1 515 nm (for RAPTOR1B-YFP) Ex2 687nm (for SnRK1α1-mCherry). Detection bandwidth: PMT1 520–560 nm (RAPTOR1B-YFP) PMT2 605–630 nm (SnRK1α1-mCherry) PMT3 687–752 nm (plastid autofluorescence). Images were analysed with the LAS AF Lite software from Leica (pictures and diameter blots) and Fiji^90^ (2D surface blots).

For BiFC experiments constructs were transformed into *A. tumefaciens* C58C1 and transiently expressed by *Agrobacterium*-infiltration in *N. benthamiana*. The BiFC-induced YFP fluorescence was detected by confocal laser scanning microscopy (LSM510; Zeiss) 48 h post infiltration. The specimens were examined using the LD LCI Plan-Apochromat 253/0.8 water-immersion objective for detailed images with excitation using the argon laser (458 or 488 nm line for BiFC and chlorophyll autofluorescence). The emitted light passed the primary beam-splitting mirrors at 458/514 nm and was separated by a secondary beam splitter at 515 nm. Fluorescence was detected with filter sets as follows: on channel 3, 530–560 band pass; and on channel 1, 678–743 for red autofluorescence of chlorophyll. All samples were investigated using the same microscope settings. Images were processed using the ZEN software package (Zeiss).

### Kinase assays

SnRK1α1 cloning/expression was done as described previously^38^. Briefly, SnRK1α1 (AT3G01090.1/3) was reverse transcribed from total cDNA (*A. thaliana* rosette leafs), cloned into pGEX-4T which contains an IPTG inducible promoter (GE Healthcare) and transformed into *Escherichia coli* (strain ER2566) for expression. *E. coli* cells carrying the expression plasmids were grown in LB at 37 °C under vigorous shaking until an OD600 of 0.4 was reached. At this point cultures were transferred to room temperature and SnRK1α1 expression was induced by addition of IPTG to a final concentration of 1 mM. After 4 h cells were harvested. The protein containing an N-terminal GST tag was purified using Glutathione Sepharose 4B (GE Healthcare) according to the manufacturer’s instructions. In brief, cells were homogenized in GST binding buffer (50 mM Tris pH 8, 20 mM MgSO_4_, 5 mM EDTA, 2 mM DTT, 1% Triton-X-100) by sonification, followed by centrifugation. Proteins were purified by incubating the supernatant with Glutathione Sepharose beads at 4 °C for 1 h, followed by washing with GST binding buffer and elution with GST elution buffer (50 mM Tris pH 8, 10 mM reduced glutathione). Proteins were stored at −80 °C in GST elution buffer containing 20% glycerol. RAPTOR1B (AT3G08850) cDNA was PCR amplified from total cDNA and cloned into a modified pQE80L-YFP vector resulting in a 6xHIS-YFP-RAPTOR1B fusion ORF under the control of an IPTG inducible promoter (see attached vector map, Supplementary File S3). Plasmid was transformed into the *E. coli* BL21 protein expression strain and expression of RAPTOR1B protein was done similarly to SnRK1α1 expression. RAPTOR1B was purified via HiTrap Chelating HP affinity chromatography (GE Healthcare) according to manufacturer’s instructions. Briefly, protein was homogenized and sonicated in homogenization buffer (20 mM NaPO_4_ pH 7.4, 300 mM NaCl, 5 mM imidazole) to obtain RAPTOR1B protein. HiTrap columns were charged with 100 mM NiSO_4_ and equilibrated with biding buffer (20 mM NaPO_4_ pH 7.4, 300 mM NaCl, 5 mM imidazole, 0.5% TritonX 100). Protein was loaded into the column and then washed with washing buffer (20 mM NaPO_4_ pH 7.4, 300 mM NaCl, 50 mM imidazole, 0.5% TritonX 100). Finally, protein was eluted with elution buffer (20 mM NaPO_4_ pH 7.4, 300 mM NaCl, 300 mM imidazole) and stored at −80 °C in elution buffer containing 10% of glycerol.

For *in vitro* kinase assays, recombinantly expressed GST-SnRK1α1 and YFP-Raptor1b were incubated together in kinase reaction buffer (10 mM Hepes pH 7.5, 20 mM MgCl_2_, 10 mM DTT, 50 μM ATP and 2 μCi gamma ^32^P labelled ATP) for 35 min at room temperature. The reaction was stopped by adding 4x Leammli buffer, samples were boiled at 94 °C for 4 min and separated by SDS-PAGE. Dried gels were exposed on a Storage Phosphor Screen (GE Healthcare).

### GC-MS analysis of the primary metabolome

Frozen leaf rosettes were homogenized with pestle and mortar. Primary metabolites were extracted and derivatized as described previously^91^. Gas chromatography coupled to mass spectrometry (GC-MS) analysis was performed on an Agilent 6890 gas chromatograph (Agilent Technologies^®^, Santa Clara, CA, USA) coupled to a LECO Pegasus^®^ 4D GCxGC-TOF mass spectrometer (LECO Corporation, St. Joseph, MI, USA). Compounds were separated on an Agilent HP5MS column (length: 30 m length, diameter: 0.25 mm, film: 0.25 μm). Deconvolution of the total ion chromatograms was performed using the LECO Chromatof^®^ software. For absolute quantification, calibration curves were recorded comprising 5 different substrate concentrations within the linear range of detection.

### Prediction of Jacobian matrices based on metabolite covariance information

The functional connection of GC-MS metabolomics data with causal biochemical network information was performed, as previously described^92^, by the approximation of the biochemical Jacobian matrix. This approximation directly connects the covariance matrix C, which was built from the experimental metabolomics data, with a metabolic network structure (Supplementary File S4). The metabolic network can be visualized and simulated with freely available SBML tools, e.g. CellDesigner™ (http://www.celldesigner.org/). Linkage of covariance data with the network structure follows equation 1^68^,^93^,^94^:


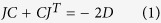


J represents the Jacobian matrix and D is a fluctuation matrix which integrates a Gaussian noise function simulating metabolic fluctuations. In context of a metabolic network, entries of the Jacobian matrix J represent the elasticity of reaction rates to any change of metabolite concentrations which are characterized by equation 2:


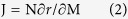


N is the stoichiometric matrix or a metabolic interaction matrix if reactions and reactants have been modified in the original network. r represents the rates for each reaction and M represents metabolite concentrations. As stated before, the Jacobian approximation comprises the stochastic term D. Therefore, we performed 10^4^ Jacobian approximations for each genotype and time point, and normalized the medians to the squared interquartile distances. All calculations of Jacobian matrices were performed based on a modified version of the toolbox COVAIN^94^ within the numerical software environment Matlab^®^ (V8.4.0 R2014b).

## Additional Information

**How to cite this article**: Nukarinen, E. *et al*. Quantitative phosphoproteomics reveals the role of the AMPK plant ortholog SnRK1 as a metabolic master regulator under energy deprivation. *Sci. Rep.* **6**, 31697; doi: 10.1038/srep31697 (2016).

## Supplementary Material

Supplementary Information
