# Supplementary material for: Quantitative phosphoproteomics reveals the role of the AMPK plant ortholog SnRK1 as a metabolic master regulator under energy deprivation
Source: Sci Rep. 2016 Aug 22;6:31697. doi: 10.1038/srep31697 (PMC4992866; doi:10.1038/srep31697)
Supplement: Supplementary Information [file srep31697-s1.pdf]

Quantitative phosphoproteomics reveals the role of SnRK1 as metabolic master regulator under energy deprivation

Ella Nukarinen[1,*], Thomas Nägele[1,2,*], Lorenzo Pedrotti[3,*], Bernhard Wurzinger[1], Andrea Mair[1], Ramona Landgraf[4], Frederik Börnke[4,5], Johannes Hanson[6], Markus Teige[1], Elena Baena-Gonzalez[7], Wolfgang Dröge-Laser[3], Wolfram Weckwerth[1,2]

[1] Department of Ecogenomics and Systems Biology, University of Vienna, Vienna, Austria

[2] Vienna Metabolomics Center (VIME), University of Vienna, Vienna, Austria

[3] Julius-von-Sachs-Institut, Julius-Maximilians-Universität Würzburg, Würzburg, Germany

[4] Plant Health, Plant Metabolism Group, Leibniz-Institute of Vegetable and Ornamental Crops, Großbeeren, Germany

[5] Institute of Biochemistry and Biology, University of Potsdam, Potsdam, Germany

[6] Department of Plant Physiology, Umeå University, Umeå, Sweden

[7] Instituto Gulbenkian de Ciência, Oeiras, Portugal

[*] These authors contributed equally to this work

Correspondence and requests for materials should be addressed to W.W. (email: wolfram.weckwerth@univie.ac.at)

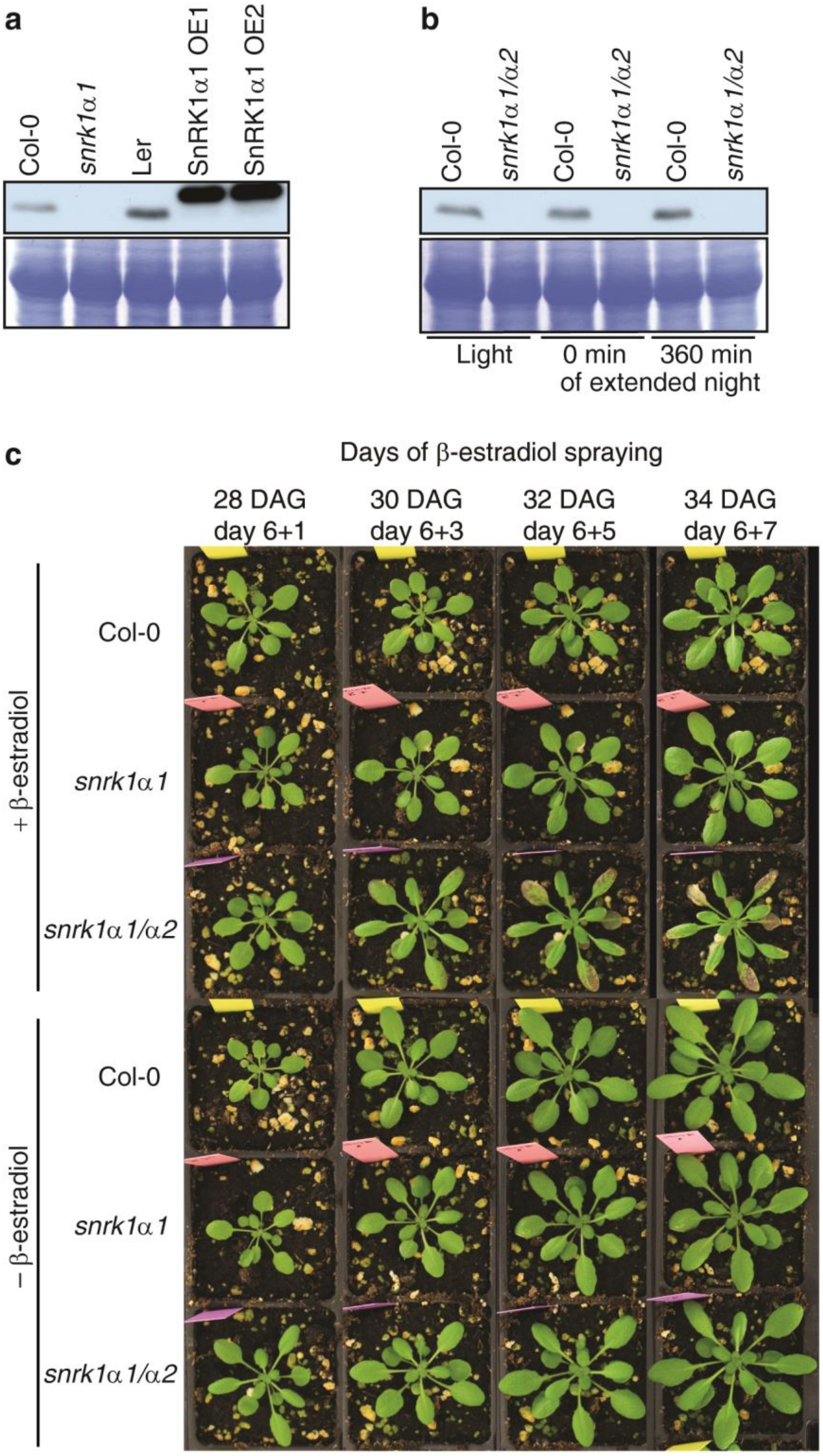

Supplementary Figure S1. (**a**) Abundance of SnRK1α1 in Col-0 wild type, *snrk1α1* ko, Ler wild type, SnRK1 OE1, and SnRK1 OE2. Immunodetection was done with anti-AKIN10 antibody. (**b**) The expression of SnRK1α1 in Col-0 and *snrk1α1/α2* in the time series experiment for 3 time points (Light, 0 min and 360 min of extended night) is shown with anti-AKIN10 antibody. (**c**) Phenotype of Col-0 wild type, *snrk1α1* and *snrk1α1/α2* mutants in 12 h/12 h day/night cycle. Knocking down of SnRK1α2 was started after 22 days after germination (DAG). Plants were either sprayed with 10 µM β-estradiol or with same solution without β-estradiol. SnRK1α2 protein is relatively stable and it decreases after 5-6 days after start of β-estradiol treatment (Pedrotti et al. in preparation). The knock down of SnRK1α2 is labelled as day 6 + number of days of knock down.

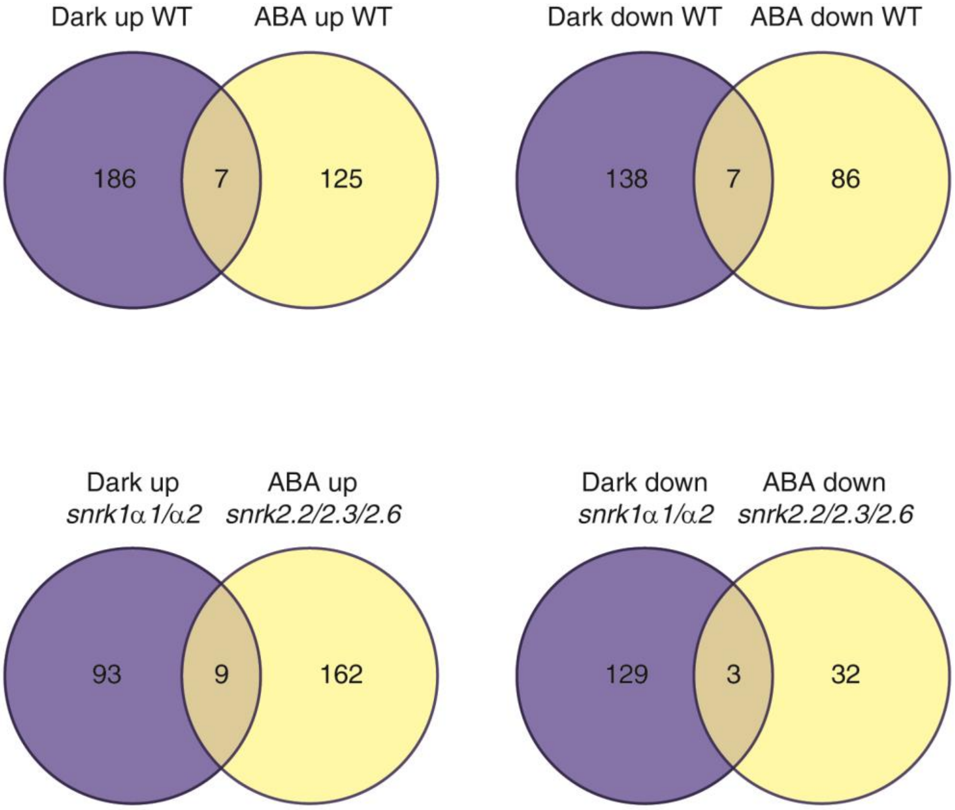

Supplementary Figure S2. Comparison of phosphorylated proteins in *snrk1α1/α2* mutant and *snrk2.2/2.3/2.3* triple mutant (Wang, P. et al. (2013) Quantitative phosphoproteomics identifies SnRK2 protein kinase substrates and reveals the effectors of abscisic acid action. Proc Natl Acad Sci USA 110, 11205-11210). All values of the dark time points were averaged and compared to the averaged light values (fold change >1.5 and <0.67).

a

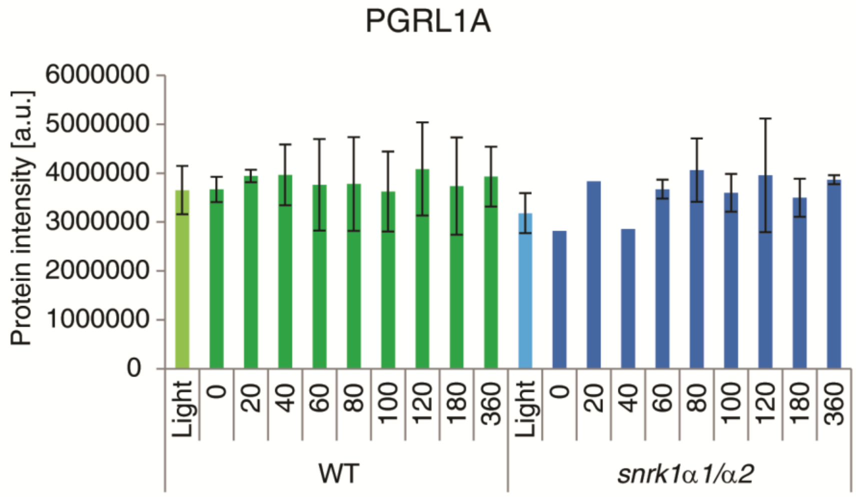
PGRL1A
Protein intensity [a.u.]
6000000
5000000
4000000
3000000
2000000
1000000
0
Light
0
20
40
60
80
100
120
180
360
WT
snrk1α1/α2

b

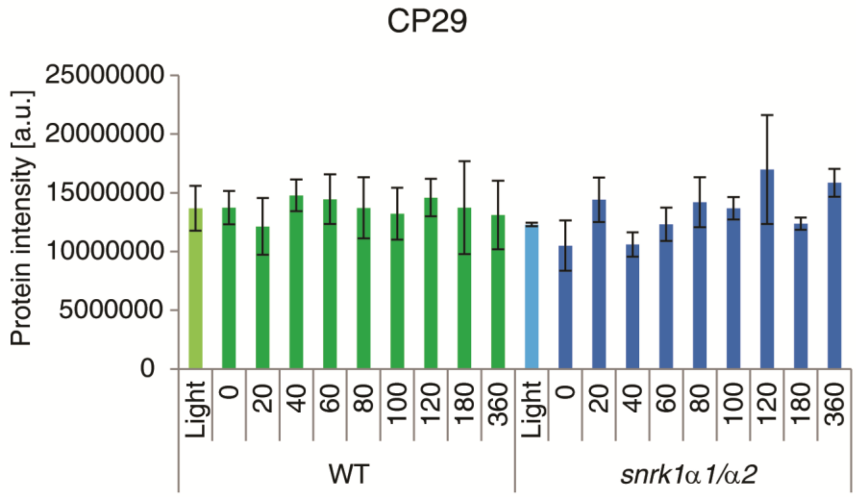
CP29
Protein intensity [a.u.]
25000000
20000000
15000000
10000000
5000000
0
Light
0
20
40
60
80
100
120
180
360
WT
snrk1α1/α2

c

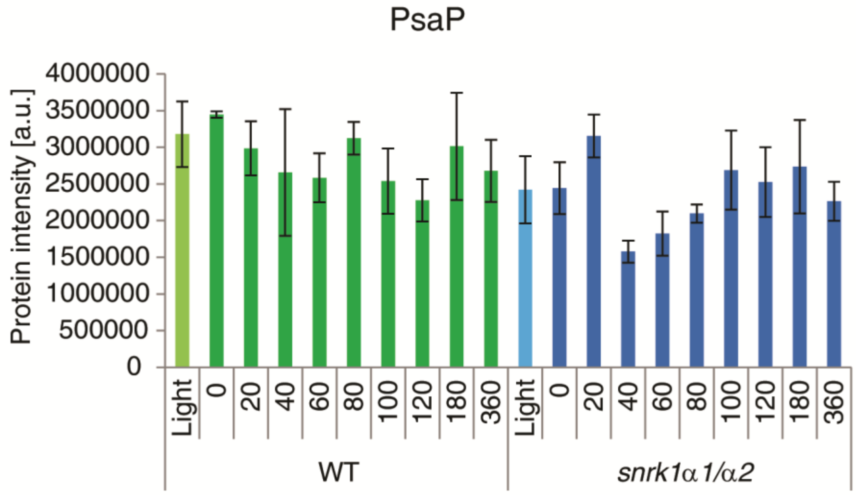
PsaP
Protein intensity [a.u.]
4000000
3500000
3000000
2500000
2000000
1500000
1000000
500000
0
Light
0
20
40
60
80
100
120
180
360
WT
snrk1α1/α2

d

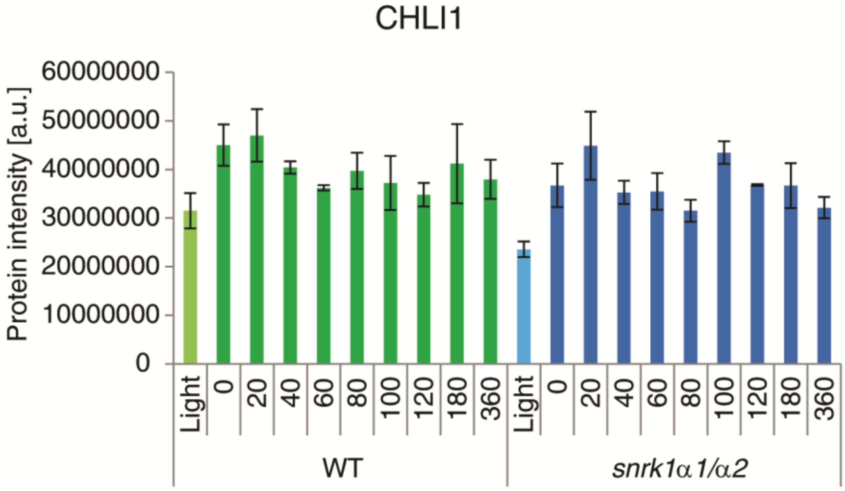

e

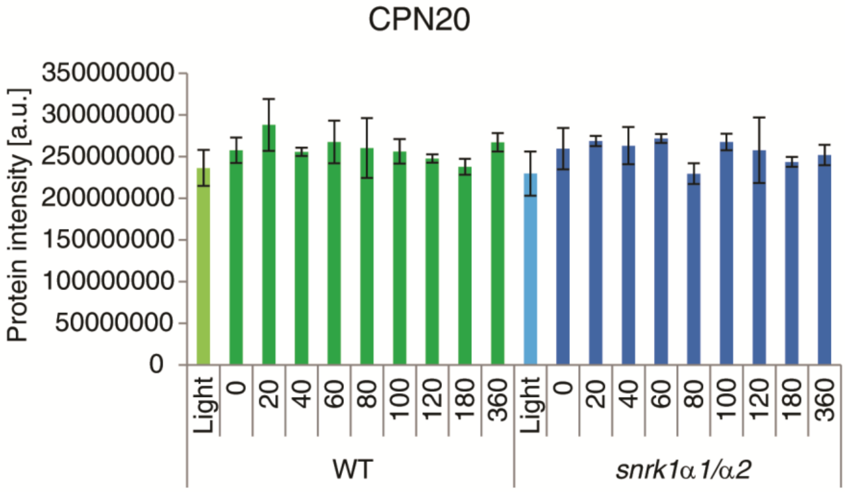

f

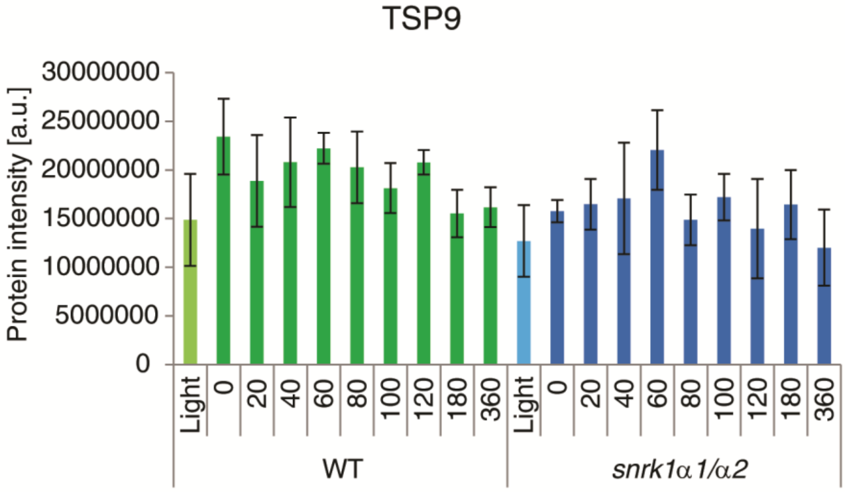

Supplementary Figure S3. Total protein abundance of chloroplastic proteins that were differentially phosphorylated in *snrk1α1/α2* mutant and in Col-0 wild type. x-axis represents timepoints (minutes) in extended night.

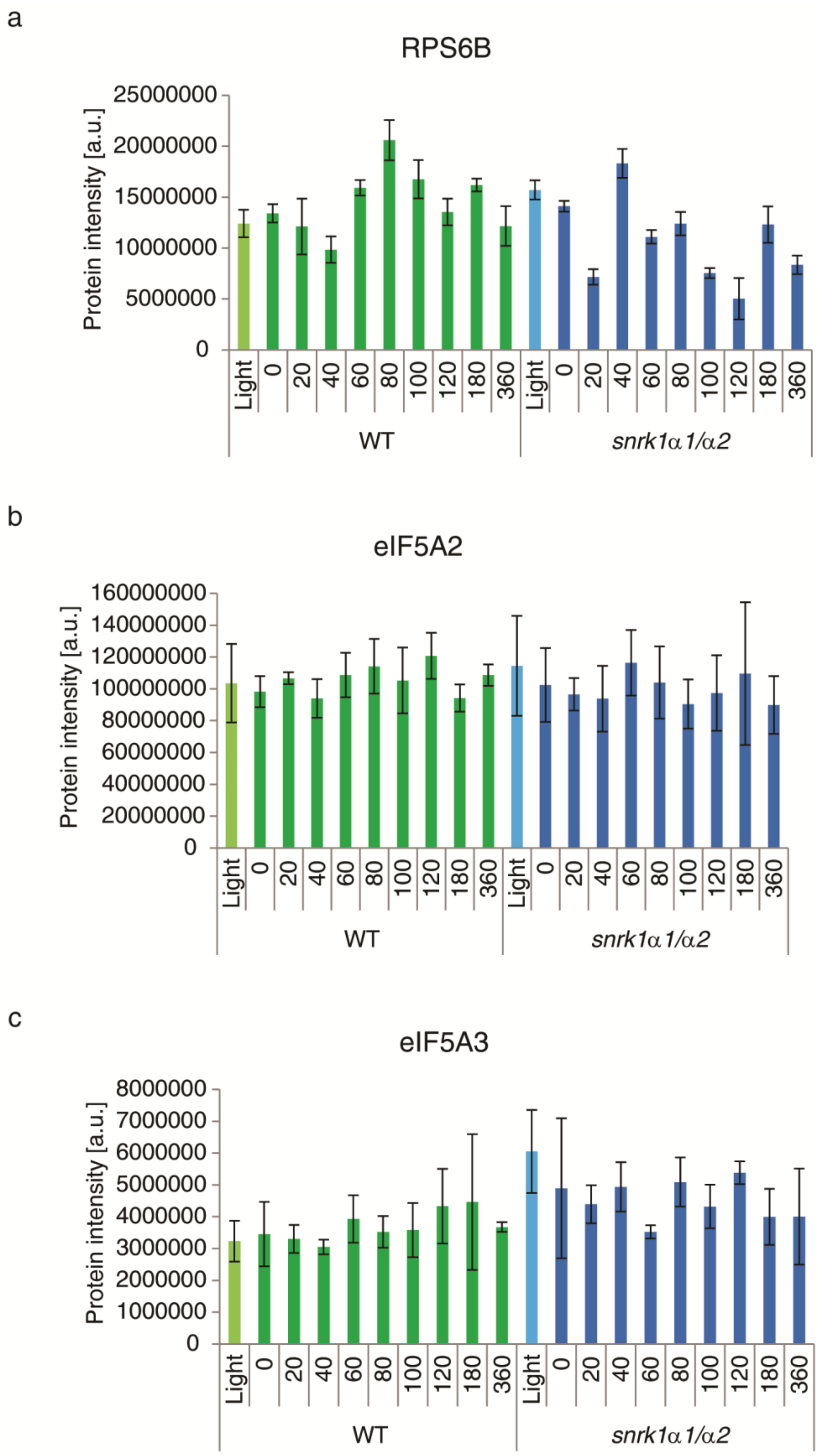

Supplementary Figure S4. Total protein abundance of proteins having function in proteins translation that were differentially phosphorylated in *snrk1α1/α2* mutant and in Col-0 wild type. x-axis represents timepoints (minutes) in extended night.

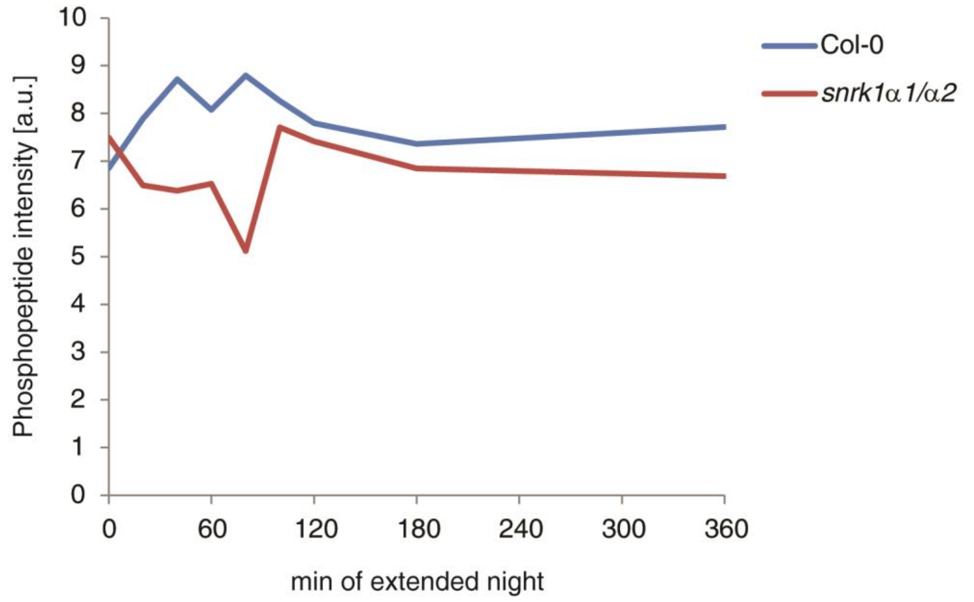

Supplementary Figure S5. Abundance of fructose-2,6-bisphosphatase peptide SLS^{303}ASSFLIDTK during extended night.

Supplementary File S1

```
LOCUS       Atraptor\1b\in\p       16714 bp    DNA     circular     13-
OCT-2015
SOURCE
  ORGANISM
COMMENT     This file is created by Vector NTI
            http://www.invitrogen.com/
COMMENT     VNTDATE|747660692|
COMMENT     VNTDBDATE|747661067|
COMMENT     LSOWNER|
COMMENT     VNTNAME|Atraptor 1b in pBIN19 YFP (ML747)|
COMMENT     VNTAUTHORNAME|Bernhard Wurzinger|
FEATURES             Location/Qualifiers
     promoter        12119..12535
                     /vntifkey="29"
                     /label=1x35\Promoter
     enhancer        12544..12674
                     /vntifkey="9"
                     /label=TL\(TEV\Leader)
     misc_feature    12525..12567
                     /vntifkey="21"
                     /label=möglicherweise\kein\XhoI
     terminator      747..950
                     /vntifkey="43"
                     /label=Terminator\(CaMV)
     polyA_signal    916..922
                     /vntifkey="25"
                     /label=Poly(A)\Signal
     misc_feature    7..735
                     /vntifkey="21"
                     /label=YFP
     misc_feature    730..765
                     /vntifkey="21"
                     /label=XbaI\nicht\da!!!
     primer_bind     complement(70..88)
                     /vntifkey="28"
                     /label=EGFP-N1-R
     rep_origin      complement(7127..7744)
                     /standard_name="oriV"
                     /vntifkey="33"
                     /label=oriV
                     /note="shows similarity to GenBank Accession Number
M20134"
     misc_feature    complement(6781..7052)
                     /vntifkey="21"
                     /label=kilA
                     /note="shows similarity to kilA: GenBank Accession
Number M62846"
     CDS             complement(6430..6780)
                     /vntifkey="4"
                     /label=NPTIII
                     /note="shows similarity to NPTIII gene: GenBank
Accession Number V01547"
     transposon      complement(5660..6429)
                     /vntifkey="44"
                     /label=IS1
                     /note="shows similarity to transposable element IS1:
GenBank Accession Number X58999"
     misc_feature    complement(3185..4666)
                     /vntifkey="21"
                     /label=trfA
```

```
                     /note="shows similarity to trfA: GenBank Accession
Number X00713"
     misc_feature    complement(2142..3184)
                     /vntifkey="21"
                     /label=tetA
                     /note="shows similarity to tetA: GenBank Accession
Number L13842"
     CDS             complement(1424..1554)
                     /vntifkey="4"
                     /label=lacI
                     /note="shows similarity to lacI: GenBank Accession
Number J01636"
     rep_origin      complement(1123..1423)
                     /standard_name="M13 ori"
                     /vntifkey="33"
                     /label=M13\ori
                     /note="shows similarity to GenBank Accession Number
X02513"
     CDS             complement(11837..11977)
                     /vntifkey="4"
                     /label=lacI
                     /note="shows similarity to lacI: GenBank Accession
Number J01636"
     misc_feature    complement(11395..11836)
                     /vntifkey="21"
                     /label=geneIII
                     /note="shows similarity to geneIII: GenBank
Accession Number V00604"
     repeat_region   complement(10101..10262)
                     /vntifkey="34"
                     /label=RB
                     /note="shows similarity to T-DNA right border:
GenBank Accession Number J01826"
     misc_feature    complement(8905..10088)
                     /vntifkey="21"
                     /label=tetA
                     /note="shows similarity to tetA: GenBank Accession
Number X75761"
     rep_origin      complement(8534..8912)
                     /standard_name="ColE1 ori"
                     /vntifkey="33"
                     /label=ColE1\ori
                     /note="shows similarity to GenBank Accession Number
V00268"
     misc_feature    complement(7757..8540)
                     /vntifkey="21"
                     /label=traF
                     /note="shows similarity to traF: GenBank Accession
Number X54459"
     repeat_unit     complement(1555..1702)
                     /vntifkey="35"
                     /label=LB
     CDS             complement(4667..5659)
                     /vntifkey="4"
                     /label=NPTIII
     primer_bind     complement(12038..12052)
                     /vntifkey="28"
                     /label=M13rev
     primer_bind     complement(974..990)
                     /vntifkey="28"
```

```
                     /label=M13fwd
     primer_bind     1266..1287
                     /vntifkey="28"
                     /label=LBa1\primer
     primer_bind     1465..1486
                     /vntifkey="28"
                     /label=LBb1\primer
     CDS             10568..11116
                     /vntifkey="4"
                     /label=Basta-R
     misc_feature    12680..16713
                     /vntifkey="21"
                     /label=Atraptor1b
BASE COUNT     3810 a      4583 c      4305 g      4016 t
ORIGIN
        1 ggccgcatgg tgagcaaggg cgaggagctg ttcaccgggg tggtgcccat
cctggtcgag
       61 ctggacggcg acgtaaacgg ccacaagttc agcgtgtccg gcgagggcga
gggcgatgcc
      121 acctacggca agctgaccct gaagttcatc tgcaccaccg gcaagctgcc
cgtgccctgg
      181 cccaccctcg tgaccacctt cggctacggc ctacagtgct tcgcccgcta
ccccgaccac
      241 atgaagcagc acgacttctt caagtccgcc atgcccgaag gctacgtcca
ggagcgcacc
      301 atcttcttca aggacgacgg caactacaag acccgcgccg aggtgaagtt
cgagggcgac
      361 accctggtga accgcatcga gctgaagggc atcgacttca aggaggacgg
caacatcctg
      421 gggcacaagc tggagtacaa ctacaacagc cacaacgtct atatcatggc
cgacaagcag
      481 aagaacggca tcaaggtgaa cttcaagatc cgccacaaca tcgaggacgg
cagcgtgcag
      541 ctcgccgacc actaccagca gaacaccccc atcggcgacg gccccgtgct
gctgcccgac
      601 aaccactacc tgagctacca gtccgccctg agcaaagacc ccaacgagaa
gcgcgatcac
      661 atggtcctgc tggagttcgt gaccgccgcc gggatcactc tcggcatgga
cgagctgtac
      721 aagagcgggc gctaggctct agagtccgca aaaatcacca gtctctctct
acaaatctat
      781 ctctctctat tttctccaga ataatgtgtg agtagttccc agataaggga
attagggttc
      841 ttatagggtt tcgctcatgt gttgagcata taagaaaccc ttagtatgta
tttgtatttg
      901 taaaatactt ctatcaataa aatttctaat tcctaaaacc aaaatccagt
gacctactag
      961 tgagctcgaa ttcactggcc gtcgttttac aacgtcgtga ctgggaaaac
cctggcgtta
     1021 cccaacttaa tcgccttgca gcacatcccc ctttcgccag ctggcgtaat
agcgaagagg
     1081 cccgcaccga tcgcccttcc caacagttgc gcagcctgaa tggcgcccgc
tcctttcgct
     1141 ttcttccctt cctttctcgc cacgttcgcc ggctttcccc gtcaagctct
aaatcggggg
     1201 ctccctttag ggttccgatt tagtgcttta cggcacctcg accccaaaaa
acttgatttg
     1261 ggtgatggtt cacgtagtgg gccatcgccc tgatagacgg tttttcgccc
tttgacgttg
```

1321 gagtccacgt tctttaatag tggactcttg ttccaaactg gaacaacact
caaccctatc
1381 tcgggctatt cttttgattt ataagggatt ttgccgattt cggaaccacc
atcaaacagg
1441 attttcgcct gctggggcaa accagcgtgg accgcttgct gcaactctct
cagggccagg
1501 cggtgaaggg caatcagctg ttgcccgtct cactggtgaa aagaaaaacc
accccagtac
1561 attaaaaacg tccgcaatgt gttattaagt tgtctaagcg tcaatttgtt
tacaccacaa
1621 tatatcctgc caccagccag ccaacagctc cccgaccggc agctcggcac
aaaatcacca
1681 ctcgatacag gcagcccatc agtccgggac ggcgtcagcg ggagagccgt
tgtaaggcgg
1741 cagactttgc tcatgttacc gatgctattc ggaagaacgg caactaagct
gccgggtttg
1801 aaacacggat gatctcgcgg agggtagcat gttgattgta acgatgacag
agcgttgctg
1861 cctgtgatca aatatcatct ccctcgcaga gatccgaatt atcagccttc
ttattcattt
1921 ctcgcttaac cgtgacaggc tgtcgatctt gagaactatg ccgacataat
aggaaatcgc
1981 tggataaagc cgctgaggaa gctgagtggc gctatttctt tagaagtgaa
cgttgacgat
2041 atcaactccc ctatccattg ctcaccgaat ggtacaggtc ggggacccga
agttccgact
2101 gtcggcctga tgcatccccg gctgatcgac cccagatctg gggctgagaa
agcccagtaa
2161 ggaaacaact gtaggttcga gtcgcgagat cccccggaac caaaggaagt
aggttaaacc
2221 cgctccgatc aggccgagcc acgccaggcc gagaacattg gttcctgtag
gcatcgggat
2281 tggcggatca aacactaaag ctactggaac gagcagaagt cctccggccg
ccagttgcca
2341 ggcggtaaag gtgagcagag gcacgggagg ttgccacttg cgggtcagca
cggttccgaa
2401 cgccatggaa accgcccccg ccaggcccgc tgcgacgccg acaggatcta
gcgctgcgtt
2461 tggtgtcaac accaacagcg ccacgcccgc agttccgcaa atagcccca
ggaccgccat
2521 caatcgtatc gggctaccta gcagagcggc agagatgaac acgaccatca
gcggctgcac
2581 agcgcctacc gtcgccgcga ccccgcccgg caggcggtag accgaaataa
acaacaagct
2641 ccagaatagc gaaatattaa gtgcgccgag gatgaagatg cgcatccacc
agattcccgt
2701 tggaatctgt cggacgatca tcacgagcaa taaacccgcc ggcaacgccc
gcagcagcat
2761 accggcgacc cctcggcctc gctgttcggg ctccacgaaa acgccggaca
gatgcgcctt
2821 gtgagcgtcc ttggggccgt cctcctgttt gaagaccgac agcccaatga
tctcgccgtc
2881 gatgtaggcg ccgaatgcca cggcatctcg caaccgttca gcgaacgcct
ccatgggctt
2941 tttctcctcg tgctcgtaaa cggacccgaa catctctgga gctttcttca
gggccgacaa
3001 tcggatctcg cggaaatcct gcacgtcggc cgctccaagc cgtcgaatct
gagccttaat
3061 cacaattgtc aattttaatc ctctgtttat cggcagttcg tagagcgcgc
cgtgcgtccc

3121 gagcgatact gagcgaagca agtgcgtcga gcagtgcccg cttgttcctg aaatgccagt
3181 aaagcgctgg ctgctgaacc cccagccgga actgacccca caaggcccta gcgtttgcaa
3241 tgcaccaggt catcattgac ccaggcgtgt tccaccaggc cgctgcctcg caactcttcg
3301 caggcttcgc cgacctgctc gcgccacttc ttcacgcggg tggaatccga tccgcacatg
3361 aggcggaagg tttccagctt gagcgggtac ggctcccggt gcgagctgaa atagtcgaac
3421 atccgtcggg ccgtcggcga cagcttgcgg tacttctccc atatgaattt cgtgtagtgg
3481 tcgccagcaa acagcacgac gatttcctcg tcgatcagga cctggcaacg ggacgttttc
3541 ttgccacggt ccaggacgcg gaagcggtgc agcagcgaca ccgattccag gtgcccaacg
3601 cggtcggacg tgaagcccat cgccgtcgcc tgtaggcgcg acaggcattc ctcggccttc
3661 gtgtaatacc ggccattgat cgaccagccc aggtcctggc aaagctcgta gaacgtgaag
3721 gtgatcggct cgccgatagg ggtgcgcttc gcgtactcca acacctgctg ccacaccagt
3781 tcgtcatcgt cggcccgcag ctcgacgccg gtgtaggtga tcttcacgtc cttgttgacg
3841 tggaaaatga ccttgttttg cagcgcctcg cgcgggattt tcttgttgcg cgtggtgaac
3901 agggcagagc gggccgtgtc gtttggcatc gctcgcatcg tgtccggcca cggcgcaata
3961 tcgaacaagg aaagctgcat ttccttgatc tgctgcttcg tgtgtttcag caacgcggcc
4021 tgcttggcct cgctgacctg ttttgccagg tcctcgccgg cggtttttcg cttcttggtc
4081 gtcatagttc ctcgcgtgtc gatggtcatc gacttcgcca aacctgccgc ctcctgttcg
4141 agacgacgcg aacgctccac ggcggccgat ggcgcgggca gggcaggggg agccagttgc
4201 acgctgtcgc gctcgatctt ggccgtagct tgctggacca tcgagccgac ggactggaag
4261 gtttcgcggg gcgcacgcat gacggtgcgg cttgcgatgg tttcggcatc ctcggcggaa
4321 aaccccgcgt cgatcagttc ttgcctgtat gccttccggt caaacgtccg attcattcac
4381 cctccttgcg ggattgcccc gactcacgcc ggggcaatgt gcccttattc ctgatttgac
4441 ccgcctggtg ccttggtgtc cagataatcc accttatcgg caatgaagtc ggtcccgtag
4501 accgtctggc cgtccttctc gtacttggta ttccgaatct tgccctgcac gaataccagc
4561 gaccccttgc ccaaatactt gccgtgggcc tcggcctgag agccaaaaca cttgatgcgg
4621 aagaagtcgg tgcgctcctg cttgtcgccg gcatcgttgc gccacatcta ggtactaaaa
4681 caattcatcc agtaaaatat aatattttat tttctcccaa tcaggcttga tccccagtaa
4741 gtcaaaaaat agctcgacat actgttcttc cccgatatcc tccctgatcg accggacgca
4801 gaaggcaatg tcataccact tgtccgccct gccgcttctc ccaagatcaa taaagccact
4861 tactttgcca tctttcacaa agatgttgct gtctcccagg tcgccgtggg aaaagacaag

4921 ttcctcttcg ggcttttccg tctttaaaaa atcatacagc tcgcgcggat
ctttaaatgg
4981 agtgtcttct tcccagtttt cgcaatccac atcggccaga tcgttattca
gtaagtaatc
5041 caattcggct aagcggctgt ctaagctatt cgtataggga caatccgata
tgtcgatgga
5101 gtgaaagagc ctgatgcact ccgcatacag ctcgataatc ttttcagggc
tttgttcatc
5161 ttcatactct tccgagcaaa ggacgccatc ggcctcactc atgagcagat
tgctccagcc
5221 atcatgccgt tcaaagtgca ggacctttgg aacaggcagc tttccttcca
gccatagcat
5281 catgtccttt tcccgttcca catcataggt ggtcccttta taccggctgt
ccgtcatttt
5341 taaatatagg ttttcatttt ctcccaccag cttatatacc ttagcaggag
acattccttc
5401 cgtatctttt acgcagcggt atttttcgat cagtttttc aattccggtg
atattctcat
5461 tttagccatt tattatttcc ttcctctttt ctacagtatt taaagatacc
ccaagaagct
5521 aattataaca agacgaactc caattcactg ttccttgcat tctaaaacct
taaataccag
5581 aaaacagctt tttcaaagtt gttttcaaag ttggcgtata acatagtatc
gacggagccg
5641 attttgaaac cacaattatg ggtgatgctg ccaacttact gatttagtgt
atgatggtgt
5701 ttttgaggtg ctccagtggc ttctgtgtct atcagctgtc cctcctgttc
agctactgac
5761 ggggtggtgc gtaacggcaa aagcaccgcc ggacatcagc gctatctctg
ctctcactgc
5821 cgtaaaacat ggcaactgca gttcacttac accgcttctc aacccggtac
gcaccagaaa
5881 atcattgata tggccatgaa tggcgttgga tgccgggcaa cagcccgcat
tatgggcgtt
5941 ggcctcaaca cgattttacg tcacttaaaa aactcaggcc gcagtcggta
acctcgcgca
6001 tacagccggg cagtgacgtc atcgtctgcg cggaaatgga cgaacagtgg
ggctatgtcg
6061 gggctaaatc gcgccagcgc tggctgtttt acgcgtatga cagtctccgg
aagacggttg
6121 ttgcgcacgt attcggtgaa cgcactatgg cgacgctggg gcgtcttatg
agcctgctgt
6181 caccctttga cgtggtgata tggatgacgg atggctggcc gctgtatgaa
tcccgcctga
6241 agggaaagct gcacgtaatc agcaagcgat atacgcagcg aattgagcgg
cataacctga
6301 atctgaggca gcacctggca cggctgggac ggaagtcgct gtcgttctca
aaatcggtgg
6361 agctgcatga caaagtcatc gggcattatc tgaacataaa acactatcaa
taagttggag
6421 tcattaccca attatgatag aatttacaag ctataaggtt attgtcctgg
gtttcaagca
6481 ttagtccatg caagttttta tgctttgccc attctataga tatattgata
agcgcgctgc
6541 ctatgccttg ccccctgaaa tccttacata cggcgatatc ttctatataa
aagatatatt
6601 atcttatcag tattgtcaat atattcaagg caatctgcct cctcatcctc
ttcatcctct
6661 tcgtcttggt agctttttaa atatggcgct tcatagagta attctgtaaa
ggtccaattc

6721 tcgttttcat acctcggtat aatcttacct atcacctcaa atggttcgct gggtttatcg
6781 cacccccgaa cacgagcacg gcacccgcga ccactatgcc aagaatgccc aaggtaaaaa
6841 ttgccggccc cgccatgaag tccgtgaatg ccccgacggc cgaagtgaag ggcaggccgc
6901 cacccaggcc gccgccctca ctgcccggca cctggtcgct gaatgtcgat gccagcacct
6961 gcggcacgtc aatgcttccg ggcgtcgcgc tcgggctgat cgcccatccc gttactgccc
7021 cgatcccggc aatggcaagg actgccagcg ctgccatttt tggggtgagg ccgttcgcgg
7081 ccgaggggcg cagcccctgg ggggatggga ggcccgcgtt agcgggccgg gagggttcga
7141 gaaggggggg caccccccctt cggcgtgcgc ggtcacgcgc acagggcgca gccctggtta
7201 aaaacaaggt ttataaatat tggtttaaaa gcaggttaaa agacaggtta gcggtggccg
7261 aaaaacgggc ggaaaccctt gcaaatgctg gattttctgc ctgtggacag cccctcaaat
7321 gtcaataggt gcgcccctca tctgtcagca ctctgcccct caagtgtcaa ggatcgcgcc
7381 cctcatctgt cagtagtcgc gcccctcaag tgtcaatacc gcagggcact tatccccagg
7441 cttgtccaca tcatctgtgg gaaactcgcg taaaatcagg cgttttcgcc gatttgcgag
7501 gctggccagc tccacgtcgc cggccgaaat cgagcctgcc cctcatctgt caacgccgcg
7561 ccgggtgagt cggcccctca agtgtcaacg tccgcccctc atctgtcagt gagggccaag
7621 ttttccgcga ggtatccaca acgccggcgg gcgcggtgtc tcgcacacgg cttcgacggc
7681 gtttctggcg cgtttgcagg gccatagacg gccgccagcc cagcggcgag ggcaaccagc
7741 ccggtgagcg tcgcaaaggc gctcggtctt gccttgctcg tcggtgatgt acttcaccag
7801 ctccgcgaag tcgctcttct tgatggagcg catggggacg tgcttggcaa tcacgcgcac
7861 cccccggccg ttttagcggc taaaaaagtc atggctctgc cctcgggcgg accacgccca
7921 tcatgacctt gccaagctcg tcctgcttct cttcgatctt cgccagcagg gcgaggatcg
7981 tggcatcacc gaaccgcgcc gtgcgcgggt cgtcggtgag ccagagtttc agcaggccgc
8041 ccaggcggcc caggtcgcca ttgatgcggg ccagctcgcg gacgtgctca tagtccacga
8101 cgcccgtgat tttgtagccc tggccgacgg ccagcaggta ggccgacagg ctcatgccgg
8161 ccgccgccgc cttttcctca atcgctcttc gttcgtctgg aaggcagtac accttgatag
8221 gtgggctgcc cttcctggtt ggcttggttt catcagccat ccgcttgccc tcatctgtta
8281 cgccggcggt agccggccag cctcgcagag caggattccc gttgagcacc gccaggtgcg
8341 aataagggac agtgaagaag gaacacccgc tcgcgggtgg gcctacttca cctatcctgc
8401 ccggctgacg ccgttggata caccaaggaa agtctacacg aaccctttgg caaaatcctg
8461 tatatcgtgc gaaaaaggat ggatataccg aaaaaatcgc tataatgacc ccgaagcagg

8521 gttatgcagc ggaaaagcgc cacgcttccc gaagggagaa aggcggacag
gtatccggta
8581 agcggcaggg tcggaacagg agagcgcacg agggagcttc caggggggaaa
cgcctggtat
8641 ctttatagtc ctgtcgggtt tcgccacctc tgacttgagc gtcgattttt
gtgatgctcg
8701 tcaggggggc ggagcctatg gaaaaacgcc agcaacgcgg cctttttacg
gttcctggcc
8761 ttttgctggc cttttgctca catgttcttt cctgcgttat cccctgattc
tgtggataac
8821 cgtattaccg cctttgagtg agctgatacc gctcgccgca gccgaacgac
cgagcgcagc
8881 gagtcagtga gcgaggaagc ggaagagcgc cagaaggccg ccagagaggc
cgagcgcggc
8941 cgtgaggctt ggacgctagg gcagggcatg aaaaagcccg tagcgggctg
ctacgggcgt
9001 ctgacgcggt ggaaaggggg aggggatgtt gtctacatgg ctctgctgta
gtgagtgggt
9061 tgcgctccgg cagcggtcct gatcaatcgt caccctttct cggtccttca
acgttcctga
9121 caacgagcct ccttttcgcc aatccatcga caatcaccgc gagtccctgc
tcgaacgctg
9181 cgtccggacc ggcttcgtcg aaggcgtcta tcgcggcccg caacagcggc
gagagcggag
9241 cctgttcaac ggtgccgccg cgctcgccgg catcgctgtc gccggcctgc
tcctcaagca
9301 cggccccaac agtgaagtag ctgattgtca tcagcgcatt gacggcgtcc
ccggccgaaa
9361 aacccgcctc gcagaggaag cgaagctgcg cgtcggccgt ttccatctgc
ggtgcgcccg
9421 gtcgcgtgcc ggcatggatg cgcgcgccat cgcggtaggc gagcagcgcc
tgcctgaagc
9481 tgcgggcatt cccgatcaga aatgagcgcc agtcgtcgtc ggctctcggc
accgaatgcg
9541 tatgattctc cgccagcatg gcttcggcca gtgcgtcgag cagcgcccgc
ttgttcctga
9601 agtgccagta aagcgccggc tgctgaaccc ccaaccgttc cgccagtttg
cgtgtcgtca
9661 gaccgtctac gccgacctcg ttcaacaggt ccagggcggc acggatcact
gtattcggct
9721 gcaactttgt catgcttgac actttatcac tgataaacat aatatgtcca
ccaacttatc
9781 agtgataaag aatccgcgcg ttcaatcgga ccagcggagg ctggtccgga
ggccagacgt
9841 gaaacccaac ataccccctga tcgtaattct gagcactgtc gcgctcgacg
ctgtcggcat
9901 cggcctgatt atgccggtgc tgccgggcct cctgcgcgat ctggttcact
cgaacgacgt
9961 caccgcccac tatggcattc tgctggcgct gtatgcgttg gtgcaatttg
cctgcgcacc
10021 tgtgctgggc gcgctgtcgg atcgtttcgg gcggcggcca atcttgctcg
tctcgctggc
10081 cggcgccaga tctggggaac cctgtggttg gcatgcacat acaaatggac
gaacggataa
10141 acctttcac gcccttttaa atatccgatt attctaataa acgctctttt
ctcttaggtt
10201 tacccgccaa tatatcctgt caaacactga tagtttaaac tgaaggcggg
aaacgacaat
10261 ctgatcatga gcggagaatt aagggagtca cgttatgacc cccgccgatg
acgcgggaca

10321 agccgtttta cgtttggaac tgacagaacc gcaacgttga aggagccact cagccgcggg
10381 tttctggagt ttaatgagct aagcacatac gtcagaaacc attattgcgc gttcaaaagt
10441 cgcctaaggt cactatcagc tagcaaatat ttcttgtcaa aaatgctcca ctgacgttcc
10501 ataaattccc ctcggtatcc aattagagtc tcatattcac tctcaactcg atcgaggggg
10561 atctaccatg agcccagaac gacgcccggc cgacatccgc cgtgccaccg aggcggacat
10621 gccggcggtc tgcaccatcg tcaaccacta catccagaca agcacggtca acttccgtac
10681 cgagccgcag gaaccgcagg agtggacgga cgacctcgtc cgtctgcggg agcgctatcc
10741 ctggctcgtc gccgaggtgg acggcgaggt cgccggcatc gcctacgcgg gtccctggaa
10801 ggcacgcaac gcctacgact ggacggccga gtcgaccgtg tacgtctccc cccgccacca
10861 gcggacggga ctgggctcca cgctctacac ccacctgctg aagtccctgg aggcacaggg
10921 cttcaagagc gtggtcgctg tcatcgggct gcccaacgac ccgagcgtgc gcatgcacga
10981 ggcgctcgga tatgcccccc gcggcatgct gcgggcggcc ggcttcaagc acgggaactg
11041 gcatgacgtg ggtttctggc agctggactt cagcctgccg gtgccgcccc gtccggtcct
11101 gcccgtcacc gaaatctgat gacccctaga gtcaagcaga tcgttcaaac atttggcaat
11161 aaagtttctt aagattgaat cctgttgccg gtcttgcgat gattatcata taatttctgt
11221 tgaattacgt taagcatgta ataattaaca tgtaatgcat gacgttattt atgagatggg
11281 tttttatgat tagagtcccg caattataca tttaatacgc gatagaaaac aaaatatagc
11341 gcgcaaacta ggataaatta tcgcgcgcgg tgtcatctat gttactagat cgggcctcct
11401 gtcaatgctg gcggcggctc tggtggtggt tctggtggcg gctctgaggg tggtggctct
11461 gagggtggcg gttctgaggg tggcggctct gagggaggcg gttccggtgg tggctctggt
11521 tccggtgatt ttgattatga aaagatggca aacgctaata agggggctat gaccgaaaat
11581 gccgatgaaa acgcgctaca gtctgacgct aaaggcaaac ttgattctgt cgctactgat
11641 tacggtgctg ctatcgatgg tttcattggt gacgtttccg gccttgctaa tggtaatggt
11701 gctactggtg attttgctgg ctctaattcc caaatggctc aagtcggtga cggtgataat
11761 tcacctttaa tgaataattt ccgtcaatat ttaccttccc tccctcaatc ggttgaatgt
11821 cgccctttttg tctttggccc aatacgcaaa ccgcctctcc ccgcgcgttg gccgattcat
11881 taatgcagct ggcacgacag gtttcccgac tggaaagcgg gcagtgagcg caacgcaatt
11941 aatgtgagtt agctcactca ttaggcaccc caggctttac actttatgct tccggctcgt
12001 atgttgtgtg gaattgtgag cggataacaa tttcacacag gaaacagcta tgaccatgat
12061 tacgccaagc ttgcatgcct gcaggtcgac tctagaggat ccccgggtac cgtccgtcaa

12121 catggtggag cacgacacac ttgtctactc caaaaatatc aaagatacag tctcagaaga
12181 ccaaagggca attgagactt ttcaacaaag ggtaatatcc ggaaacctcc tcggattcca
12241 ttgcccagct atctgtcact ttattgtgaa gatagtggaa aaggaaggtg gctcctacaa
12301 atgccatcat tgcgataaag gaaaggccat cgttgaagat gcctctgccg acagtggtcc
12361 caaagatgga cccccaccca cgaggagcat cgtggaaaaa gaagacgttc caaccacgtc
12421 ttcaaagcaa gtggattgat gtgatatctc cactgacgta agggatgacg cacaatccca
12481 ctatccttcg caagaccctt cctctatata aggaagttca tttcatttgg agaggacctc
12541 gacctcaaca caacatatac aaaacaaacg aatctcaagc aatcaagcat tctacttcta
12601 ttgcagcaat ttaaatcatt tcttttaaag caaaagcaat tttctgaaaa ttttcaccat
12661 ttacgaacga tagccgggga tggcattagg agacttaatg gtgtctcggt tctcgcaatc
12721 ttctgtttct ttggtctcca atcaccgtta cgacgaagac tgtgtctcta gtcacgatga
12781 cggtgattcc cggaggaagg attctgaggc taagagcagt agtagctacg ggaatggtac
12841 aacggaggga gccgccaccg ccaccagcat ggcttacttg cctcagacta ttgtgctctg
12901 cgagcttaga cacgacgcat ccgaggcttc tgctcccttg gggacttctg agatcgtatt
12961 ggtccccaaa tggcggctta aagaacgaat gaaaacagga tgtgtagctt tagttctgtg
13021 tttaaacatt actgttgatc cgccggatgt tataaagata tctccttgtg ctagaatcga
13081 ggcatggata gatccatttt ctatggcacc gcctaaagct ctcgagacaa ttggaaaaaa
13141 tttgagcact cagtatgaga gatggcaacc cagggcccgc tataaggttc agcttgatcc
13201 gacagtagat gaggtgagga agctgtgctt gacttgtcgg aaatatgcaa agaccgagag
13261 agttctattc cattataatg ggcatggtgt gccaaaacct acagctaatg gtgaaatttg
13321 ggtatttaac aagagttata cacagtacat tcccttgcca atcagtgaac ttgattcctg
13381 gttgaagaca ccctccatct atgttttcga ctgctctgct gctaggatga ttctgaatgc
13441 ctttgctgag cttcatgatt ggggttcttc tggttcctct ggatcctcaa gggactgcat
13501 tctacttgct gcttgtgatg tacatgagac acttcctcag agcgttgagt ttccagctga
13561 tgtttttacg tcttgcctga caacgcccat taaaatggcg ttgaaatggt tctgcaggcg
13621 ttcgcttctg aaagaaatta tcgatgaatc acttatcgac aggattccag gccggcaaaa
13681 cgatcgtaag acattgttag gggagttgaa ctggattttc acagcagtga cggatacaat
13741 tgcttggaat gtgcttcctc atgaactttt ccagagatta ttcagacagg acttgttggt
13801 cgctagcctt ttccggaatt tcttacttgc tgagagaata atgcggtccg caaactgtaa
13861 tccaatatct caccctatgc tgcctcctac gcatcaacat catatgtggg atgcatggga

13921 catggctgct gaaatctgtc tttctcagct tccccaactt gttctggacc caagcacaga
13981 gttccagcca agtccgtttt ttactgagca actgacagct tttgaggtgt ggcttgatca
14041 tggatctgag cataagaagc caccggagca gttacctatt gttcttcagg tgttacttag
14101 ccagtgccat cggtttcgtg ctcttgtact tcttggaaga tttcttgata tgggttcatg
14161 ggctgtggat ctggccttgt ctgttggaat attcccatat gtgctgaagc ttctgcaaac
14221 aacaacgaat gagctaagac agatcctggt tttcatatgg acaaaaattc ttgcacttga
14281 caagtcatgt caaattgatc ttgtgaagga tggggggcat acatacttca tacgatttct
14341 agatagctcg ggtgcatttc cagaacaacg agctatggct gcttttgttc tggctgtcat
14401 tgtggatgga catcgacgag gccaggaagc atgtcttgaa gctaatttaa ttggtgtttg
14461 tctcgggcac cttgaagcat ccagaccaag tgatccacaa ccagaaccat tgtttctaca
14521 atggctttgt ctttgtcttg gaaagttatg ggaagatttt atggaggccc aaataatggg
14581 cagggaggca aatgcttttg aaaagttggc acctctgctt tccgagcccc aacctgaggt
14641 aagggctgct gcagtttttg ccctgggtac cttgcttgat attgggtttg actctaataa
14701 aagtgtggtg gaggatgaat ttgatgatga tgaaaagatt agagccgaag acgctatcat
14761 taaaagtctc ttagatgtag tttcagatgg gagtccactt gtccgagcag aggttgccgt
14821 agctcttgca cgttttgcct ttggccacaa acagcacctt aagttagccg cagcttcata
14881 ttggaagcct cagtcaagtt ctttgcttac ttctctacct tcaatagcta aattccatga
14941 tcctgggagt gcaacaattg tttctttaca catgagtcct ctgactagag ctagcacgga
15001 tagccaacca gtggctcgtg agtctaggat ctcaagcagc cctcttggct cctctgggct
15061 gatgcaagga tctccattat ctgatgattc ttcgttacat tctgattctg gaatgatgca
15121 tgacagtgtc agcaatggag cggtccatca gccaagactg ttggataatg ctgtttattc
15181 gcagtgcgtc cgagctatgt ttgcattagc taaagatcca tctccacgta ttgcaagtct
15241 cggacggcgt gtgctttcta ttattggaat cgaacaggtt gttgcgaaac cctcgaagcc
15301 cactggccga ccaggggaag ctgcaacgac atctcacact ccacttgctg gcctagctcg
15361 ttcatcctca tggtttgata tgcatgcagg taatctgcct ttaagtttta ggactccccc
15421 ggtcagcccc cctagaacaa actacctgag tggattgagg agagtttgtt cattagagtt
15481 caggcctcat ctattgggtt cacccgactc aggattggct gatccgcttt taggcgccag
15541 tggatctgaa cggagtttgc ttccactatc aactatctac ggctggagtt gtgggcactt
15601 ttctaagcca cttcttggtg gtgcggatgc tagtcaagag attgcagcca aaagagaaga
15661 gaaagaaaaa ttcgcacttg agcatattgc aaaatgccag cactcttcta ttagcaagct

15721 caacaataat cctattgcca actgggatac aaggtttgaa acgggaacaa agacagccct
15781 ccttcaccca ttctctccta ttgtagttgc tgcagacgag aatgaacgga tcagagtgtg
15841 gaactatgag gaagcaactc ttctcaatgg ctttgacaat catgattttc ctgacaaagg
15901 aatttcaaag ctctgcctca tcaatgaact tgacgactct ctgctacttg ttgcatcatg
15961 cgatgggtcg gtccggatat ggaaaaacta tgcaactaag ggtaaacaaa agcttgttac
16021 tgggttttct tcaatccagg gtcacaagcc cggtgcccgt gacttgaacg ctgtcgtgga
16081 ctggcaacaa cagtccggtt acctgtatgc ttctggggag acgtcaacag tcacactttg
16141 ggacctggag aaagaacagc ttgtcagatc tgttccctct gaatcagaat gtggagttac
16201 agcactttct gcttctcaag tgcacggagg ccaactcgct gctggttttg ctgatggatc
16261 tttgagactc tatgatgttc ggtcacctga accgcttgtc tgcgcgactc ggcctcatca
16321 gaaagttgaa agggtggttg gcctcagttt tcaacctgga cttgaccccg caaaggtggt
16381 gagtgcatca caggcgggtg acatacagtt tcttgacctt agaacaacaa gggacacata
16441 cctgacgatt gatgcacaca ggggttcact cacggcctta gctgttcaca gacacgctcc
16501 aataatcgcg agtggatctg caaaacagct cattaaagta ttcagtcttc aaggggaaca
16561 actagggata atccgctact acccatcctt catggctcaa aagattggat cagtgagttg
16621 cctcacattt catccgtacc aggttctgct agcagctgga gctgctgact catttgtctc
16681 catatacacc cacgacaact cgcaagcaag atgc
//

Supplementary File S2

```
LOCUS       pBIN19\AKIN10\mC       14196 bp    DNA     circular     11-
FEB-2015
SOURCE
  ORGANISM
COMMENT     This file is created by Vector NTI
            http://www.invitrogen.com/
COMMENT     VNTDATE|725363803|
COMMENT     VNTDBDATE|725363803|
COMMENT     LSOWNER|
COMMENT     VNTNAME|pBIN19 AKIN10 mCherry (355)|
COMMENT     VNTAUTHORNAME|lala|
FEATURES             Location/Qualifiers
     rep_origin      complement(7107..7724)
                     /standard_name="oriV"
                     /vntifkey="33"
                     /label=oriV
                     /note="shows similarity to GenBank Accession Number
M20134"
     misc_feature    complement(6761..7032)
                     /vntifkey="21"
                     /label=kilA
                     /note="shows similarity to kilA: GenBank Accession
Number M62846"
     CDS             complement(6410..6760)
                     /vntifkey="4"
                     /label=NPTIII
                     /note="shows similarity to NPTIII gene: GenBank
Accession Number V01547"
     transposon      complement(5640..6409)
                     /vntifkey="44"
                     /label=IS1
                     /note="shows similarity to transposable element IS1:
GenBank Accession Number X58999"
     misc_feature    complement(3165..4646)
                     /vntifkey="21"
                     /label=trfA
                     /note="shows similarity to trfA: GenBank Accession
Number X00713"
     misc_feature    complement(2122..3164)
                     /vntifkey="21"
                     /label=tetA
                     /note="shows similarity to tetA: GenBank Accession
Number L13842"
     CDS             complement(1404..1534)
                     /vntifkey="4"
                     /label=lacI
                     /note="shows similarity to lacI: GenBank Accession
Number J01636"
     rep_origin      complement(1103..1403)
                     /standard_name="M13 ori"
                     /vntifkey="33"
                     /label=M13\ori
                     /note="shows similarity to GenBank Accession Number
X02513"
     CDS             complement(11817..11957)
                     /vntifkey="4"
                     /label=lacI
                     /note="shows similarity to lacI: GenBank Accession
Number J01636"
     misc_feature    complement(11375..11816)
```

```
                     /vntifkey="21"
                     /label=geneIII
                     /note="shows similarity to geneIII: GenBank
Accession Number V00604"
     repeat_region   complement(10081..10242)
                     /vntifkey="34"
                     /label=RB
                     /note="shows similarity to T-DNA right border:
GenBank Accession Number J01826"
     misc_feature    complement(8885..10068)
                     /vntifkey="21"
                     /label=tetA
                     /note="shows similarity to tetA: GenBank Accession
Number X75761"
     rep_origin      complement(8514..8892)
                     /standard_name="ColE1 ori"
                     /vntifkey="33"
                     /label=ColE1\ori
                     /note="shows similarity to GenBank Accession Number
V00268"
     misc_feature    complement(7737..8520)
                     /vntifkey="21"
                     /label=traF
                     /note="shows similarity to traF: GenBank Accession
Number X54459"
     repeat_unit     complement(1535..1682)
                     /vntifkey="35"
                     /label=LB
     CDS             complement(4647..5639)
                     /vntifkey="4"
                     /label=NPTIII
     primer_bind     complement(12018..12032)
                     /vntifkey="28"
                     /label=M13rev
     primer_bind     complement(954..970)
                     /vntifkey="28"
                     /label=M13fwd
     primer_bind     1246..1267
                     /vntifkey="28"
                     /label=LBa1\primer
     primer_bind     1445..1466
                     /vntifkey="28"
                     /label=LBb1\primer
     CDS             10548..11096
                     /vntifkey="4"
                     /label=Basta-R
     misc_feature    7..717
                     /vntifkey="21"
                     /label=mCherry
     misc_feature    12501..12543
                     /vntifkey="21"
                     /label=möglicherweise\kein\XhoI
     enhancer        12520..12650
                     /vntifkey="9"
                     /label=TL\(TEV\Leader)
     promoter        12095..12511
                     /vntifkey="29"
                     /label=1x35\Promoter
     misc_feature    12658..14193
                     /vntifkey="21"
```

/label=AKIN10\splicing\form\1\(und3)
BASE COUNT 3227 a 3961 c 3723 g 3285 t
ORIGIN
1 ggccgcatgg tgagcaaggg cgaggaggat aacatggcca tcatcaagga gttcatgcgc
61 ttcaaggtgc acatggaggg ctccgtgaac ggccacgagt tcgagatcga gggcgagggc
121 gagggccgcc cctacgaggg cacccagacc gccaagctga aggtgaccaa gggtggcccc
181 ctgcccttcg cctgggacat cctgtcccct cagttcatgt acggctccaa ggcctacgtg
241 aagcaccccg ccgacatccc cgactacttg aagctgtcct tccccgaggg cttcaagtgg
301 gagcgcgtga tgaacttcga ggacggcggc gtggtgaccg tgacccagga ctcctccctg
361 caggacggcg agttcatcta caaggtgaag ctgcgcggca ccaacttccc ctccgacggc
421 cccgtaatgc agaagaagac catgggctgg gaggcctcct ccgagcggat gtaccccgag
481 gacggcgccc tgaagggcga gatcaagcag aggctgaagc tgaaggacgg cggccactac
541 gacgctgagg tcaagaccac ctacaaggcc aagaagcccg tgcagctgcc cggcgcctac
601 aacgtcaaca tcaagttgga catcacctcc cacaacgagg actacaccat cgtggaacag
661 tacgaacgcg ccgagggccg ccactccacc ggcggcatgg acgagctgta caagtaatct
721 agagtccgca aaaatcacca gtctctctct acaaatctat ctctctctat tttctccaga
781 ataatgtgtg agtagttccc agataaggga attagggttc ttatagggtt tcgctcatgt
841 gttgagcata taagaaaccc ttagtatgta tttgtatttg taaaatactt ctatcaataa
901 aatttctaat tcctaaaacc aaaatccagt gacctactag tgagctcgaa ttcactggcc
961 gtcgttttac aacgtcgtga ctgggaaaac cctggcgtta cccaacttaa tcgccttgca
1021 gcacatcccc ctttcgccag ctggcgtaat agcgaagagg cccgcaccga tcgcccttcc
1081 caacagttgc gcagcctgaa tggcgcccgc tcctttcgct ttcttccctt cctttctcgc
1141 cacgttcgcc ggctttcccc gtcaagctct aaatcggggg ctccctttag ggttccgatt
1201 tagtgcttta cggcacctcg accccaaaaa acttgatttg ggtgatggtt cacgtagtgg
1261 gccatcgccc tgatagacgg tttttcgccc tttgacgttg gagtccacgt tctttaatag
1321 tggactcttg ttccaaactg gaacaacact caaccctatc tcgggctatt cttttgattt
1381 ataagggatt ttgccgattt cggaaccacc atcaaacagg attttcgcct gctggggcaa
1441 accagcgtgg accgcttgct gcaactctct cagggccagg cggtgaaggg caatcagctg
1501 ttgcccgtct cactggtgaa aagaaaaacc accccagtac attaaaaacg tccgcaatgt
1561 gttattaagt tgtctaagcg tcaatttgtt tacaccacaa tatatcctgc caccagccag
1621 ccaacagctc cccgaccggc agctcggcac aaaatcacca ctcgatacag gcagcccatc

1681 agtccgggac ggcgtcagcg ggagagccgt tgtaaggcgg cagactttgc tcatgttacc
1741 gatgctattc ggaagaacgg caactaagct gccgggtttg aaacacggat gatctcgcgg
1801 agggtagcat gttgattgta acgatgacag agcgttgctg cctgtgatca aatatcatct
1861 ccctcgcaga gatccgaatt atcagccttc ttattcattt ctcgcttaac cgtgacaggc
1921 tgtcgatctt gagaactatg ccgacataat aggaaatcgc tggataaagc cgctgaggaa
1981 gctgagtggc gctatttctt tagaagtgaa cgttgacgat atcaactccc ctatccattg
2041 ctcaccgaat ggtacaggtc ggggacccga agttccgact gtcggcctga tgcatccccg
2101 gctgatcgac cccagatctg gggctgagaa agcccagtaa ggaaacaact gtaggttcga
2161 gtcgcgagat cccccggaac caaaggaagt aggttaaacc cgctccgatc aggccgagcc
2221 acgccaggcc gagaacattg gttcctgtag gcatcgggat tggcggatca aacactaaag
2281 ctactggaac gagcagaagt cctccggccg ccagttgcca ggcggtaaag gtgagcagag
2341 gcacgggagg ttgccacttg cgggtcagca cggttccgaa cgccatggaa accgcccccg
2401 ccaggcccgc tgcgacgccg acaggatcta gcgctgcgtt tggtgtcaac accaacagcg
2461 ccacgcccgc agttccgcaa atagccccca ggaccgccat caatcgtatc gggctaccta
2521 gcagagcggc agagatgaac acgaccatca gcggctgcac agcgcctacc gtcgccgcga
2581 ccccgcccgg caggcggtag accgaaataa acaacaagct ccagaatagc gaaatattaa
2641 gtgcgccgag gatgaagatg cgcatccacc agattcccgt tggaatctgt cggacgatca
2701 tcacgagcaa taaacccgcc ggcaacgccc gcagcagcat accggcgacc cctcggcctc
2761 gctgttcggg ctccacgaaa acgccggaca gatgcgcctt gtgagcgtcc ttggggccgt
2821 cctcctgttt gaagaccgac agcccaatga tctcgccgtc gatgtaggcg ccgaatgcca
2881 cggcatctcg caaccgttca gcgaacgcct ccatgggctt tttctcctcg tgctcgtaaa
2941 cggacccgaa catctctgga gctttcttca gggccgacaa tcggatctcg cggaaatcct
3001 gcacgtcggc cgctccaagc cgtcgaatct gagccttaat cacaattgtc aattttaatc
3061 ctctgtttat cggcagttcg tagagcgcgc cgtgcgtccc gagcgatact gagcgaagca
3121 agtgcgtcga gcagtgcccg cttgttcctg aaatgccagt aaagcgctgg ctgctgaacc
3181 cccagccgga actgacccca caaggcccta gcgtttgcaa tgcaccaggt catcattgac
3241 ccaggcgtgt tccaccaggc cgctgcctcg caactcttcg caggcttcgc cgacctgctc
3301 gcgccacttc ttcacgcggg tggaatccga tccgcacatg aggcggaagg tttccagctt
3361 gagcgggtac ggctcccggt gcgagctgaa atagtcgaac atccgtcggg ccgtcggcga
3421 cagcttgcgg tacttctccc atatgaattt cgtgtagtgg tcgccagcaa acagcacgac

3481 gatttcctcg tcgatcagga cctggcaacg ggacgttttc ttgccacggt ccaggacgcg
3541 gaagcggtgc agcagcgaca ccgattccag gtgcccaacg cggtcggacg tgaagcccat
3601 cgccgtcgcc tgtaggcgcg acaggcattc ctcggccttc gtgtaatacc ggccattgat
3661 cgaccagccc aggtcctggc aaagctcgta gaacgtgaag gtgatcggct cgccgatagg
3721 ggtgcgcttc gcgtactcca acacctgctg ccacaccagt tcgtcatcgt cggcccgcag
3781 ctcgacgccg gtgtaggtga tcttcacgtc cttgttgacg tggaaaatga ccttgttttg
3841 cagcgcctcg cgcgggattt tcttgttgcg cgtggtgaac agggcagagc gggccgtgtc
3901 gtttggcatc gctcgcatcg tgtccggcca cggcgcaata tcgaacaagg aaagctgcat
3961 ttccttgatc tgctgcttcg tgtgtttcag caacgcggcc tgcttggcct cgctgacctg
4021 ttttgccagg tcctcgccgg cggtttttcg cttcttggtc gtcatagttc ctcgcgtgtc
4081 gatggtcatc gacttcgcca aacctgccgc ctcctgttcg agacgacgcg aacgctccac
4141 ggcggccgat ggcgcgggca gggcaggggg agccagttgc acgctgtcgc gctcgatctt
4201 ggccgtagct tgctggacca tcgagccgac ggactggaag gtttcgcggg gcgcacgcat
4261 gacggtgcgg cttgcgatgg tttcggcatc ctcggcggaa aaccccgcgt cgatcagttc
4321 ttgcctgtat gccttccggt caaacgtccg attcattcac cctccttgcg ggattgcccc
4381 gactcacgcc ggggcaatgt gcccttattc ctgatttgac ccgcctggtg ccttggtgtc
4441 cagataatcc accttatcgg caatgaagtc ggtcccgtag accgtctggc cgtccttctc
4501 gtacttggta ttccgaatct tgccctgcac gaataccagc gaccccttgc ccaaatactt
4561 gccgtgggcc tcggcctgag agccaaaaca cttgatgcgg aagaagtcgg tgcgctcctg
4621 cttgtcgccg gcatcgttgc gccacatcta ggtactaaaa caattcatcc agtaaaatat
4681 aatattttat tttctcccaa tcaggcttga tccccagtaa gtcaaaaaat agctcgacat
4741 actgttcttc cccgatatcc tccctgatcg accggacgca gaaggcaatg tcataccact
4801 tgtccgccct gccgcttctc ccaagatcaa taaagccact tactttgcca tctttcacaa
4861 agatgttgct gtctcccagg tcgccgtggg aaaagacaag ttcctcttcg ggcttttccg
4921 tctttaaaaa atcatacagc tcgcgcggat ctttaaatgg agtgtcttct tcccagtttt
4981 cgcaatccac atcggccaga tcgttattca gtaagtaatc caattcggct aagcggctgt
5041 ctaagctatt cgtataggga caatccgata tgtcgatgga gtgaaagagc ctgatgcact
5101 ccgcatacag ctcgataatc ttttcagggc tttgttcatc ttcatactct tccgagcaaa
5161 ggacgccatc ggcctcactc atgagcagat tgctccagcc atcatgccgt tcaaagtgca
5221 ggacctttgg aacaggcagc tttccttcca gccatagcat catgtccttt tcccgttcca

5281 catcataggt ggtcccttta taccggctgt ccgtcatttt taaatatagg
ttttcatttt
5341 ctcccaccag cttatatacc ttagcaggag acattccttc cgtatctttt
acgcagcggt
5401 atttttcgat cagtttttc aattccggtg atattctcat tttagccatt
tattatttcc
5461 ttcctctttt ctacagtatt taaagatacc ccaagaagct aattataaca
agacgaactc
5521 caattcactg ttccttgcat tctaaaacct taaataccag aaaacagctt
tttcaaagtt
5581 gttttcaaag ttggcgtata acatagtatc gacggagccg attttgaaac
cacaattatg
5641 ggtgatgctg ccaacttact gatttagtgt atgatggtgt ttttgaggtg
ctccagtggc
5701 ttctgtgtct atcagctgtc cctcctgttc agctactgac ggggtggtgc
gtaacggcaa
5761 aagcaccgcc ggacatcagc gctatctctg ctctcactgc cgtaaaacat
ggcaactgca
5821 gttcacttac accgcttctc aacccggtac gcaccagaaa atcattgata
tggccatgaa
5881 tggcgttgga tgccgggcaa cagcccgcat tatgggcgtt ggcctcaaca
cgattttacg
5941 tcacttaaaa aactcaggcc gcagtcggta acctcgcgca tacagccggg
cagtgacgtc
6001 atcgtctgcg cggaaatgga cgaacagtgg ggctatgtcg gggctaaatc
gcgccagcgc
6061 tggctgtttt acgcgtatga cagtctccgg aagacggttg ttgcgcacgt
attcggtgaa
6121 cgcactatgg cgacgctggg gcgtcttatg agcctgctgt caccctttga
cgtggtgata
6181 tggatgacgg atggctggcc gctgtatgaa tcccgcctga agggaaagct
gcacgtaatc
6241 agcaagcgat atacgcagcg aattgagcgg cataacctga atctgaggca
gcacctggca
6301 cggctgggac ggaagtcgct gtcgttctca aaatcggtgg agctgcatga
caaagtcatc
6361 gggcattatc tgaacataaa acactatcaa taagttggag tcattaccca
attatgatag
6421 aatttacaag ctataaggtt attgtcctgg gtttcaagca ttagtccatg
caagtttta
6481 tgctttgccc attctataga tatattgata agcgcgctgc ctatgccttg
ccccctgaaa
6541 tccttacata cggcgatatc ttctatataa aagatatatt atcttatcag
tattgtcaat
6601 atattcaagg caatctgcct cctcatcctc ttcatcctct tcgtcttggt
agcttttaa
6661 atatggcgct tcatagagta attctgtaaa ggtccaattc tcgttttcat
acctcggtat
6721 aatcttacct atcacctcaa atggttcgct gggtttatcg cacccccgaa
cacgagcacg
6781 gcacccgcga ccactatgcc aagaatgccc aaggtaaaaa ttgccggccc
cgccatgaag
6841 tccgtgaatg ccccgacggc cgaagtgaag ggcaggccgc cacccaggcc
gccgccctca
6901 ctgcccggca cctggtcgct gaatgtcgat gccagcacct gcggcacgtc
aatgcttccg
6961 ggcgtcgcgc tcgggctgat cgcccatccc gttactgccc cgatcccggc
aatggcaagg
7021 actgccagcg ctgccatttt tggggtgagg ccgttcgcgg ccgaggggcg
cagcccctgg

7081 ggggatggga ggcccgcgtt agcgggccgg gagggttcga gaaggggggg cacccccctt
7141 cggcgtgcgc ggtcacgcgc acagggcgca gccctggtta aaaacaaggt ttataaatat
7201 tggtttaaaa gcaggttaaa agacaggtta gcggtggccg aaaaacgggc ggaaaccctt
7261 gcaaatgctg gattttctgc ctgtggacag cccctcaaat gtcaataggt gcgcccctca
7321 tctgtcagca ctctgcccct caagtgtcaa ggatcgcgcc cctcatctgt cagtagtcgc
7381 gcccctcaag tgtcaatacc gcagggcact tatccccagg cttgtccaca tcatctgtgg
7441 gaaactcgcg taaaatcagg cgttttcgcc gatttgcgag gctggccagc tccacgtcgc
7501 cggccgaaat cgagcctgcc cctcatctgt caacgccgcg ccgggtgagt cggcccctca
7561 agtgtcaacg tccgcccctc atctgtcagt gagggccaag ttttccgcga ggtatccaca
7621 acgccggcgg gcgcggtgtc tcgcacacgg cttcgacggc gtttctggcg cgtttgcagg
7681 gccatagacg gccgccagcc cagcggcgag ggcaaccagc ccggtgagcg tcgcaaaggc
7741 gctcggtctt gccttgctcg tcggtgatgt acttcaccag ctccgcgaag tcgctcttct
7801 tgatggagcg catggggacg tgcttggcaa tcacgcgcac cccccggccg ttttagcggc
7861 taaaaaagtc atggctctgc cctcgggcgg accacgccca tcatgacctt gccaagctcg
7921 tcctgcttct cttcgatctt cgccagcagg gcgaggatcg tggcatcacc gaaccgcgcc
7981 gtgcgcgggt cgtcggtgag ccagagtttc agcaggccgc ccaggcggcc caggtcgcca
8041 ttgatgcggg ccagctcgcg gacgtgctca tagtccacga cgcccgtgat tttgtagccc
8101 tggccgacgg ccagcaggta ggccgacagg ctcatgccgg ccgccgccgc cttttcctca
8161 atcgctcttc gttcgtctgg aaggcagtac accttgatag gtgggctgcc cttcctggtt
8221 ggcttggttt catcagccat ccgcttgccc tcatctgtta cgccggcggt agccggccag
8281 cctcgcagag caggattccc gttgagcacc gccaggtgcg aataagggac agtgaagaag
8341 gaacacccgc tcgcgggtgg gcctacttca cctatcctgc ccggctgacg ccgttggata
8401 caccaaggaa agtctacacg aacccttttgg caaaatcctg tatatcgtgc gaaaaaggat
8461 ggataccgg aaaaaatcgc tataatgacc ccgaagcagg gttatgcagc ggaaaagcgc
8521 cacgcttccc gaagggagaa aggcggacag gtatccggta agcggcaggg tcggaacagg
8581 agagcgcacg agggagcttc caggggggaaa cgcctggtat ctttatagtc ctgtcgggtt
8641 tcgccacctc tgacttgagc gtcgattttt gtgatgctcg tcaggggggc ggagcctatg
8701 gaaaaacgcc agcaacgcgg cctttttacg gttcctggcc ttttgctggc cttttgctca
8761 catgttcttt cctgcgttat cccctgattc tgtggataac cgtattaccg cctttgagtg
8821 agctgatacc gctcgccgca gccgaacgac cgagcgcagc gagtcagtga gcgaggaagc

8881 ggaagagcgc cagaaggccg ccagagaggc cgagcgcggc cgtgaggctt ggacgctagg
9941 gcagggcatg aaaaagcccg tagcgggctg ctacgggcgt ctgacgcggt ggaaaggggg
9001 aggggatgtt gtctacatgg ctctgctgta gtgagtgggt tgcgctccgg cagcggtcct
9061 gatcaatcgt caccctttct cggtccttca acgttcctga caacgagcct ccttttcgcc
9121 aatccatcga caatcaccgc gagtccctgc tcgaacgctg cgtccggacc ggcttcgtcg
9181 aaggcgtcta tcgcggcccg caacagcggc gagagcggag cctgttcaac ggtgccgccg
9241 cgctcgccgg catcgctgtc gccggcctgc tcctcaagca cggccccaac agtgaagtag
9301 ctgattgtca tcagcgcatt gacggcgtcc ccggccgaaa aacccgcctc gcagaggaag
9361 cgaagctgcg cgtcggccgt ttccatctgc ggtgcgcccg gtcgcgtgcc ggcatggatg
9421 cgcgcgccat cgcggtaggc gagcagcgcc tgcctgaagc tgcgggcatt cccgatcaga
9481 aatgagcgcc agtcgtcgtc ggctctcggc accgaatgcg tatgattctc cgccagcatg
9541 gcttcggcca gtgcgtcgag cagcgcccgc ttgttcctga agtgccagta aagcgccggc
9601 tgctgaaccc ccaaccgttc cgccagtttg cgtgtcgtca gaccgtctac gccgacctcg
9661 ttcaacaggt ccagggcggc acggatcact gtattcggct gcaactttgt catgcttgac
9721 actttatcac tgataaacat aatatgtcca ccaacttatc agtgataaag aatccgcgcg
9781 ttcaatcgga ccagcggagg ctggtccgga ggccagacgt gaaacccaac ataccccctga
9841 tcgtaattct gagcactgtc gcgctcgacg ctgtcggcat cggcctgatt atgccggtgc
9901 tgccgggcct cctgcgcgat ctggttcact cgaacgacgt caccgcccac tatggcattc
9961 tgctggcgct gtatgcgttg gtgcaatttg cctgcgcacc tgtgctgggc gcgctgtcgg
10021 atcgtttcgg gcggcggcca atcttgctcg tctcgctggc cggcgccaga tctggggaac
10081 cctgtggttg gcatgcacat acaaatggac gaacggataa accttttcac gcccttttaa
10141 atatccgatt attctaataa acgctctttt ctcttaggtt tacccgccaa tatatcctgt
10201 caaacactga tagtttaaac tgaaggcggg aaacgacaat ctgatcatga gcggagaatt
10261 aagggagtca cgttatgacc cccgccgatg acgcgggaca agccgtttta cgtttggaac
10321 tgacagaacc gcaacgttga aggagccact cagccgcggg tttctggagt ttaatgagct
10381 aagcacatac gtcagaaacc attattgcgc gttcaaaagt cgcctaaggt cactatcagc
10441 tagcaaatat ttcttgtcaa aaatgctcca ctgacgttcc ataaattccc ctcggtatcc
10501 aattagagtc tcatattcac tctcaactcg atcgaggggg atctaccatg agcccagaac
10561 gacgcccggc cgacatccgc cgtgccaccg aggcggacat gccggcggtc tgcaccatcg
10621 tcaaccacta catccagaca agcacggtca acttccgtac cgagccgcag gaaccgcagg

10681 agtggacgga cgacctcgtc cgtctgcggg agcgctatcc ctggctcgtc gccgaggtgg
10741 acggcgaggt cgccggcatc gcctacgcgg gtccctggaa ggcacgcaac gcctacgact
10801 ggacggccga gtcgaccgtg tacgtctccc cccgccacca gcggacggga ctgggctcca
10861 cgctctacac ccacctgctg aagtccctgg aggcacaggg cttcaagagc gtggtcgctg
10921 tcatcgggct gcccaacgac ccgagcgtgc gcatgcacga ggcgctcgga tatgcccccc
10981 gcggcatgct gcgggcggcc ggcttcaagc acgggaactg gcatgacgtg ggtttctggc
11041 agctggactt cagcctgccg gtgccgcccc gtccggtcct gcccgtcacc gaaatctgat
11101 gacccctaga gtcaagcaga tcgttcaaac atttggcaat aaagtttctt aagattgaat
11161 cctgttgccg gtcttgcgat gattatcata taatttctgt tgaattacgt taagcatgta
11221 ataattaaca tgtaatgcat gacgttattt atgagatggg tttttatgat tagagtcccg
11281 caattataca tttaatacgc gatagaaaac aaaatatagc gcgcaaacta ggataaatta
11341 tcgcgcgcgg tgtcatctat gttactagat cgggcctcct gtcaatgctg gcggcggctc
11401 tggtggtggt tctggtggcg gctctgaggg tggtggctct gagggtggcg gttctgaggg
11461 tggcggctct gagggaggcg gttccggtgg tggctctggt tccggtgatt ttgattatga
11521 aaagatggca aacgctaata agggggctat gaccgaaaat gccgatgaaa acgcgctaca
11581 gtctgacgct aaaggcaaac ttgattctgt cgctactgat tacggtgctg ctatcgatgg
11641 tttcattggt gacgtttccg gccttgctaa tggtaatggt gctactggtg attttgctgg
11701 ctctaattcc caaatggctc aagtcggtga cggtgataat tcacctttaa tgaataattt
11761 ccgtcaatat ttaccttccc tccctcaatc ggttgaatgt cgcccttttg tctttggccc
11821 aatacgcaaa ccgcctctcc ccgcgcgttg gccgattcat taatgcagct ggcacgacag
11881 gtttcccgac tggaaagcgg gcagtgagcg caacgcaatt aatgtgagtt agctcactca
11941 ttaggcaccc caggctttac actttatgct tccggctcgt atgttgtgtg gaattgtgag
12001 cggataacaa tttcacacag gaaacagcta tgaccatgat tacgccaagc ttgcatgcct
12061 gcaggtcgac tctagaggat ccccgggtac cgtcaacatg gtggagcacg acacacttgt
12121 ctactccaaa aatatcaaag atacagtctc agaagaccaa agggcaattg agacttttca
12181 acaaagggta atatccggaa acctcctcgg attccattgc ccagctatct gtcactttat
12241 tgtgaagata gtggaaaagg aaggtggctc ctacaaatgc catcattgcg ataaaggaaa
12301 ggccatcgtt gaagatgcct ctgccgacag tggtcccaaa gatggacccc cacccacgag
12361 gagcatcgtg gaaaaagaag acgttccaac cacgtcttca aagcaagtgg attgatgtga
12421 tatctccact gacgtaaggg atgacgcaca atcccactat ccttcgcaag acccttcctc

12481 tatataagga agttcatttc atttggagag gacctcgacc tcaacacaac
atatacaaaa
12541 caaacgaatc tcaagcaatc aagcattcta cttctattgc agcaatttaa
atcatttctt
12601 ttaaagcaaa agcaattttc tgaaaatttt caccatttac gaacgatagc
cgggcccatg
12661 gatggatcag gcacaggcag tagaagtggg gtagaatcga ttctaccaaa
ttacaagctt
12721 gggagaactc ttggtattgg ttcctttggt agggtgaaga tagctgagca
tgcattgaca
12781 ggacataagg ttgctatcaa gatcctcaat cgtcgcaaaa tcaagaacat
ggagatggag
12841 gagaaagtga ggagagagat caaaatcttg agactattta tgcatcctca
catcatccgt
12901 ctctatgagg ttatagagac tcccacagat atttatcttg tcatggagta
tgtgaactct
12961 ggtgagctat ttgactatat tgttgagaag ggtagattgc aggaggatga
ggcgaggaac
13021 ttttttcagc agataatatc aggagtggaa tactgccatc gaaacatggt
ggttcacaga
13081 gacctcaagc ctgaaaactt gcttttggac tctaaatgca atgtaaagat
tgctgatttt
13141 ggcctgagca acataatgcg agatggtcat tttttgaaga caagttgtgg
aagtccaaat
13201 tatgccgctc cagaggtaat ttcgggcaag ttatatgctg gccctgaagt
agatgtctgg
13261 agctgtggtg tgatactcta cgctcttctc tgtgggacgc ttccatttga
tgatgaaaac
13321 attcccaacc tttttaagaa gataaaggga gggatataca cattacctag
ccatttatct
13381 cctggtgcta gagatttgat cccccggatg cttgtagttg accccatgaa
acgagtaacc
13441 atccctgaga tccggcaaca cccttggttc caagctcatc ttccgaggta
tttagctgtt
13501 cctcctccag atactgtgca acaggcaaaa aagattgacg aggagattct
ccaagaagtt
13561 atcaatatgg gatttgacag aaaccacctc atcgaatcgc tccgcaaccg
aacccagaat
13621 gatggcactg tgacgtacta tctgatactg gacaatcgtt tccgtgcctc
tagtggttat
13681 ctcggggctg agtttcaaga gaccatggaa ggtactcccc gtatgcatcc
agcagaaagc
13741 gttgcttcac ctgttagcca tcggcttcca ggactgatgg aatatcaagg
agttggcttg
13801 agatctcaat accctgttga gagaaaatgg gctcttggac ttcagtctcg
ggctcatccc
13861 cgtgaaataa tgacggaagt cctgaaagcc ctgcaagatt tgaatgtatg
ttggaagaag
13921 atagggcact acaacatgaa gtgcagatgg gttcctaaca gcagcgcaga
tggtatgctc
13981 agtaactcga tgcacgataa caactacttt ggagacgagt ccagcataat
agagaacgaa
14041 gcagctgtta agtcgcccaa tgttgtcaag tttgaaattc agttgtataa
aactcgggac
14101 gacaagtatc tactggattt gcagagagta caaggtcctc agttcttgtt
cttggatctg
14161 tgtgctgctt ttcttgctca gctccgagtc ctctgc
//

Supplementary File S3

```
LOCUS       Atraptor1b\YFP\i        9501 bp    DNA     circular      1-
JUL-2015
SOURCE
  ORGANISM
COMMENT     This file is created by Vector NTI
            http://www.invitrogen.com/
COMMENT     VNTDATE|738342882|
COMMENT     VNTDBDATE|738342882|
COMMENT     LSOWNER|
COMMENT     VNTNAME|Atraptor1b YFP in pQE80L fw (ML742)|
COMMENT     VNTAUTHORNAME|Bernhard Wurzinger|
FEATURES             Location/Qualifiers
     misc_feature    6..4040
                     /vntifkey="21"
                     /label=Atraptor1b
     misc_feature    8775..9500
                     /vntifkey="21"
                     /label=YFP
     promoter        8631..8711
                     /vntifkey="30"
                     /label=T5\promoter/lac\operator\element
     misc_feature    8685..8685
                     /vntifkey="21"
                     /label=T5\transcription\start
     misc_feature    8751..8768
                     /vntifkey="21"
                     /label=6x\His\tag\coding\sequence
     terminator      4081..4175
                     /vntifkey="43"
                     /label=lambda\t0\transcriptional\termination\region
     terminator      4937..5034
                     /vntifkey="43"
                     /label=rrnB\T1\transcriptional\termination\region
     misc_feature    complement(5125..6206)
                     /vntifkey="21"
                     /label=lacI
     rep_origin      6801..6801
                     /vntifkey="33"
                     /label=ColE1\origin\of\replication
     misc_feature    complement(7559..8419)
                     /vntifkey="21"
                     /label=Beta-lactamase\CDS\(AmpR)
     misc_feature    complement(5122..6081)
                     /vntifkey="21"
                     /label=lac\repressor\true\CDS
BASE COUNT     2353 a      2347 c      2367 g      2434 t
ORIGIN
        1 ccgggatggc attaggagac ttaatggtgt ctcggttctc gcaatcttct
gtttctttgg
       61 tctccaatca ccgttacgac gaagactgtg tctctagtca cgatgacggt
gattcccgga
      121 ggaaggattc tgaggctaag agcagtagta gctacgggaa tggtacaacg
gagggagccg
      181 ccaccgccac cagcatggct tacttgcctc agactattgt gctctgcgag
cttagacacg
      241 acgcatccga ggcttctgct cccttgggga cttctgagat cgtattggtc
cccaaatggc
      301 ggcttaaaga acgaatgaaa acaggatgtg tagctttagt tctgtgttta
aacattactg
```

361 ttgatccgcc ggatgttata aagatatctc cttgtgctag aatcgaggca tggatagatc
421 cattttctat ggcaccgcct aaagctctcg agacaattgg aaaaaatttg agcactcagt
481 atgagagatg gcaacccagg gcccgctata aggttcagct tgatccgaca gtagatgagg
541 tgaggaagct gtgcttgact tgtcggaaat atgcaaagac cgagagagtt ctattccatt
601 ataatgggca tggtgtgcca aaacctacag ctaatggtga aatttgggta tttaacaaga
661 gttatacaca gtacattccc ttgccaatca gtgaacttga ttcctggttg aagacaccct
721 ccatctatgt tttcgactgc tctgctgcta ggatgattct gaatgccttt gctgagcttc
781 atgattgggg ttcttctggt tcctctggat cctcaaggga ctgcattcta cttgctgctt
841 gtgatgtaca tgagacactt cctcagagcg ttgagtttcc agctgatgtt tttacgtctt
901 gcctgacaac gcccattaaa atggcgttga aatggttctg caggcgttcg cttctgaaag
961 aaattatcga tgaatcactt atcgacagga ttccaggccg gcaaaacgat cgtaagacat
1021 tgttagggga gttgaactgg attttcacag cagtgacgga tacaattgct tggaatgtgc
1081 ttcctcatga acttttccag agattattca gacaggactt gttggtcgct agccttttcc
1141 ggaatttctt acttgctgag agaataatgc ggtccgcaaa ctgtaatcca atatctcacc
1201 ctatgctgcc tcctacgcat caacatcata tgtgggatgc atgggacatg gctgctgaaa
1261 tctgtctttc tcagcttccc caacttgttc tggacccaag cacagagttc cagccaagtc
1321 cgttttttac tgagcaactg acagcttttg aggtgtggct tgatcatgga tctgagcata
1381 agaagccacc ggagcagtta cctattgttc ttcaggtgtt acttagccag tgccatcggt
1441 ttcgtgctct tgtacttctt ggaagatttc ttgatatggg ttcatgggct gtggatctgg
1501 ccttgtctgt tggaatattc ccatatgtgc tgaagcttct gcaaacaaca acgaatgagc
1561 taagacagat cctggttttc atatggacaa aaattcttgc acttgacaag tcatgtcaaa
1621 ttgatcttgt gaaggatggg gggcatacat acttcatacg atttctagat agctcgggtg
1681 catttccaga acaacgagct atggctgctt ttgttctggc tgtcattgtg gatggacatc
1741 gacgaggcca ggaagcatgt cttgaagcta atttaattgg tgtttgtctc gggcaccttg
1801 aagcatccag accaagtgat ccacaaccag aaccattgtt tctacaatgg ctttgtcttt
1861 gtcttggaaa gttatgggaa gattttatgg aggcccaaat aatgggcagg gaggcaaatg
1921 cttttgaaaa gttggcacct ctgctttccg agccccaacc tgaggtaagg gctgctgcag
1981 tttttgccct gggtaccttg cttgatattg ggtttgactc taataaaagt gtggtggagg
2041 atgaatttga tgatgatgaa aagattagag ccgaagacgc tatcattaaa agtctcttag
2101 atgtagtttc agatgggagt ccacttgtcc gagcagaggt tgccgtagct cttgcacgtt

2161 ttgcctttgg ccacaaacag caccttaagt tagccgcagc ttcatattgg aagcctcagt
2221 caagttcttt gcttacttct ctaccttcaa tagctaaatt ccatgatcct gggagtgcaa
2281 caattgtttc tttacacatg agtcctctga ctagagctag cacggatagc caaccagtgg
2341 ctcgtgagtc taggatctca agcagccctc ttggctcctc tgggctgatg caaggatctc
2401 cattatctga tgattcttcg ttacattctg attctggaat gatgcatgac agtgtcagca
2461 atggagcggt ccatcagcca agactgttgg ataatgctgt ttattcgcag tgcgtccgag
2521 ctatgtttgc attagctaaa gatccatctc cacgtattgc aagtctcgga cggcgtgtgc
2581 tttctattat tggaatcgaa caggttgttg cgaaaccctc gaagcccact ggccgaccag
2641 gggaagctgc aacgacatct cacactccac ttgctggcct agctcgttca tcctcatggt
2701 ttgatatgca tgcaggtaat ctgcctttaa gttttaggac tcccccggtc agcccccccta
2761 gaacaaacta cctgagtgga ttgaggagag tttgttcatt agagttcagg cctcatctat
2821 tgggttcacc cgactcagga ttggctgatc cgcttttagg cgccagtgga tctgaacgga
2881 gtttgcttcc actatcaact atctacggct ggagttgtgg gcacttttct aagccacttc
2941 ttggtggtgc ggatgctagt caagagattg cagccaaaag agaagagaaa gaaaaattcg
3001 cacttgagca tattgcaaaa tgccagcact cttctattag caagctcaac aataatccta
3061 ttgccaactg ggatacaagg tttgaaacgg gaacaaagac agccctcctt cacccattct
3121 ctcctattgt agttgctgca gacgagaatg aacggatcag agtgtggaac tatgaggaag
3181 caactcttct caatggcttt gacaatcatg attttcctga caaaggaatt tcaaagctct
3241 gcctcatcaa tgaacttgac gactctctgc tacttgttgc atcatgcgat gggtcggtcc
3301 ggatatggaa aaactatgca actaagggta aacaaaagct tgttactggg ttttcttcaa
3361 tccagggtca caagcccggt gcccgtgact tgaacgctgt cgtggactgg caacaacagt
3421 ccggttacct gtatgcttct ggggagacgt caacagtcac actttgggac ctggagaaag
3481 aacagcttgt cagatctgtt ccctctgaat cagaatgtgg agttacagca ctttctgctt
3541 ctcaagtgca cggaggccaa ctcgctgctg gttttgctga tggatctttg agactctatg
3601 atgttcggtc acctgaaccg cttgtctgcg cgactcggcc tcatcagaaa gttgaaaggg
3661 tggttggcct cagttttcaa cctggacttg accccgcaaa ggtggtgagt gcatcacagg
3721 cgggtgacat acagtttctt gaccttagaa caacaaggga cacatacctg acgattgatg
3781 cacacagggg ttcactcacg gccttagctg ttcacagaca cgctccaata atcgcgagtg
3841 gatctgcaaa acagctcatt aaagtattca gtcttcaagg ggaacaacta gggataatcc
3901 gctactaccc atccttcatg gctcaaaaga ttggatcagt gagttgcctc acatttcatc

3961 cgtaccaggt tctgctagca gctggagctg ctgactcatt tgtctccata tacacccacg
4021 acaactcgca agcaagatga cccgggtcga cctgcagcca agcttaatta gctgagcttg
4081 gactcctgtt gatagatcca gtaatgacct cagaactcca tctggatttg ttcagaacgc
4141 tcggttgccg ccgggcgttt tttattggtg agaatccaag ctagcttggc gagattttca
4201 ggagctaagg aagctaaaat ggagaaaaaa atcactggat ataccaccgt tgatatatcc
4261 caatggcatc gtaaagaaca ttttgaggca tttcagtcag ttgctcaatg tacctataac
4321 cagaccgttc agctggatat tacggccttt ttaaagaccg taaagaaaaa taagcacaag
4381 ttttatccgg cctttattca cattcttgcc cgcctgatga atgctcatcc ggaatttcgt
4441 atggcaatga aagacggtga gctggtgata tgggatagtg ttcacccttg ttacaccgtt
4501 ttccatgagc aaactgaaac gttttcatcg ctctggagtg aataccacga cgatttccgg
4561 cagtttctac acatatattc gcaagatgtg gcgtgttacg gtgaaaacct ggcctatttc
4621 cctaaagggt ttattgagaa tatgtttttc gtctcagcca atccctgggt gagtttcacc
4681 agttttgatt taaacgtggc caatatggac aacttcttcg cccccgtttt caccatgggc
4741 aaatattata cgcaaggcga caaggtgctg atgccgctgg cgattcaggt tcatcatgcc
4801 gtttgtgatg gcttccatgt cggcagaatg cttaatgaat tacaacagta ctgcgatgag
4861 tggcagggcg gggcgtaatt tttttaaggc agttattggt gcccttaaac gcctggggta
4921 atgactctct agcttgaggc atcaaataaa acgaaaggct cagtcgaaag actgggcctt
4981 tcgttttatc tgttgtttgt cggtgaacgc tctcctgagt aggacaaatc cgccctctag
5041 attacgtgca gtcgatgata agctgtcaaa catgagaatt gtgcctaatg agtgagctaa
5101 cttacattaa ttgcgttgcg ctcactgccc gctttccagt cgggaaacct gtcgtgccag
5161 ctgcattaat gaatcggcca acgcgcgggg agaggcggtt tgcgtattgg gcgccagggt
5221 ggtttttctt ttcaccagtg agacgggcaa cagctgattg cccttcaccg cctggccctg
5281 agagagttgc agcaagcggt ccacgctggt ttgccccagc aggcgaaaat cctgtttgat
5341 ggtggttaac ggcgggatat aacatgagct gtcttcggta tcgtcgtatc ccactaccga
5401 gatatccgca ccaacgcgca gcccggactc ggtaatggcg cgcattgcgc ccagcgccat
5461 ctgatcgttg gcaaccagca tcgcagtggg aacgatgccc tcattcagca tttgcatggt
5521 ttgttgaaaa ccggacatgg cactccagtc gccttcccgt tccgctatcg gctgaatttg
5581 attgcgagtg agatatttat gccagccagc cagacgcaga cgcgccgaga cagaacttaa
5641 tgggcccgct aacagcgcga tttgctggtg acccaatgcg accagatgct ccacgcccag
5701 tcgcgtaccg tcttcatggg agaaaataat actgttgatg ggtgtctggt cagagacatc

5761 aagaaataac gccggaacat tagtgcaggc agcttccaca gcaatggcat
cctggtcatc
5821 cagcggatag ttaatgatca gcccactgac gcgttgcgcg agaagattgt
gcaccgccgc
5881 tttacaggct tcgacgccgc ttcgttctac catcgacacc accacgctgg
cacccagttg
5941 atcggcgcga gatttaatcg ccgcgacaat ttgcgacggc gcgtgcaggg
ccagactgga
6001 ggtggcaacg ccaatcagca acgactgttt gcccgccagt tgttgtgcca
cgcggttggg
6061 aatgtaattc agctccgcca tcgccgcttc cactttttcc cgcgttttcg
cagaaacgtg
6121 gctggcctgg ttcaccacgc gggaaacggt ctgataagag acaccggcat
actctgcgac
6181 atcgtataac gttactggtt tcacattcac caccctgaat tgactctctt
ccgggcgcta
6241 tcatgccata ccgcgaaagg ttttgcacca ttcgatggtg tcggaatttc
gggcagcgtt
6301 gggtcctggc cacgggtgcg catgatctag agctgcctcg cgcgtttcgg
tgatgacggt
6361 gaaaacctct gacacatgca gctcccggag acggtcacag cttgtctgta
agcggatgcc
6421 gggagcagac aagcccgtca gggcgcgtca gcgggtgttg gcgggtgtcg
gggcgcagcc
6481 atgacccagt cacgtagcga tagcggagtg tatactggct taactatgcg
gcatcagagc
6541 agattgtact gagagtgcac catatgcggt gtgaaatacc gcacagatgc
gtaaggagaa
6601 aataccgcat caggcgctct tccgcttcct cgctcactga ctcgctgcgc
tcggtcgttc
6661 ggctgcggcg agcggtatca gctcactcaa aggcggtaat acggttatcc
acagaatcag
6721 gggataacgc aggaaagaac atgtgagcaa aaggccagca aaaggccagg
aaccgtaaaa
6781 aggccgcgtt gctggcgttt ttccataggc tccgcccccc tgacgagcat
cacaaaaatc
6841 gacgctcaag tcagaggtgg cgaaacccga caggactata aagataccag
gcgtttcccc
6901 ctggaagctc cctcgtgcgc tctcctgttc cgaccctgcc gcttaccgga
tacctgtccg
6961 cctttctccc ttcgggaagc gtggcgcttt ctcatagctc acgctgtagg
tatctcagtt
7021 cggtgtaggt cgttcgctcc aagctgggct gtgtgcacga accccccgtt
cagcccgacc
7081 gctgcgcctt atccggtaac tatcgtcttg agtccaaccc ggtaagacac
gacttatcgc
7141 cactggcagc agccactggt aacaggatta gcagagcgag gtatgtaggc
ggtgctacag
7201 agttcttgaa gtggtggcct aactacggct acactagaag gacagtattt
ggtatctgcg
7261 ctctgctgaa gccagttacc ttcggaaaaa gagttggtag ctcttgatcc
ggcaaacaaa
7321 ccaccgctgg tagcggtggt ttttttgttt gcaagcagca gattacgcgc
agaaaaaaag
7381 gatctcaaga agatcctttg atcttttcta cggggtctga cgctcagtgg
aacgaaaact
7441 cacgttaagg gattttggtc atgagattat caaaaaggat cttcacctag
atccttttaa
7501 attaaaaatg aagttttaaa tcaatctaaa gtatatatga gtaaacttgg
tctgacagtt

7561 accaatgctt aatcagtgag gcacctatct cagcgatctg tctatttcgt tcatccatag
7621 ttgcctgact ccccgtcgtg tagataacta cgatacggga gggcttacca tctggcccca
7681 gtgctgcaat gataccgcga gacccacgct caccggctcc agatttatca gcaataaacc
7741 agccagccgg aagggccgag cgcagaagtg gtcctgcaac tttatccgcc tccatccagt
7801 ctattaattg ttgccgggaa gctagagtaa gtagttcgcc agttaatagt ttgcgcaacg
7861 ttgttgccat tgctacaggc atcgtggtgt cacgctcgtc gtttggtatg gcttcattca
7921 gctccggttc ccaacgatca aggcgagtta catgatcccc catgttgtgc aaaaaagcgg
7981 ttagctcctt cggtcctccg atcgttgtca gaagtaagtt ggccgcagtg ttatcactca
8041 tggttatggc agcactgcat aattctctta ctgtcatgcc atccgtaaga tgcttttctg
8101 tgactggtga gtactcaacc aagtcattct gagaatagtg tatgcggcga ccgagttgct
8161 cttgcccggc gtcaatacgg gataataccg cgccacatag cagaacttta aaagtgctca
8221 tcattggaaa acgttcttcg gggcgaaaac tctcaaggat cttaccgctg ttgagatcca
8281 gttcgatgta acccactcgt gcacccaact gatcttcagc atcttttact ttcaccagcg
8341 tttctgggtg agcaaaaaca ggaaggcaaa atgccgcaaa aaagggaata agggcgacac
8401 ggaaatgttg aatactcata ctcttccttt ttcaatatta ttgaagcatt tatcagggtt
8461 attgtctcat gagcggatac atatttgaat gtatttagaa aaataaacaa ataggggttc
8521 cgcgcacatt tccccgaaaa gtgccacctg acgtctaaga aaccattatt atcatgacat
8581 taacctataa aaataggcgt atcacgaggc cctttcgtct tcacctcgag aaatcataaa
8641 aaatttattt gctttgtgag cggataacaa ttataataga ttcaattgtg agcggataac
8701 aatttcacac agaattcatt aaagaggaga aattaactat gagaggatcg catcaccatc
8761 accatcacgg atccatggtg agcaagggcg aggagctgtt caccggggtg gtgcccatcc
8821 tggtcgagct ggacggcgac gtaaacggcc acaagttcag cgtgtccggc gagggcgagg
8881 gcgatgccac ctacggcaag ctgaccctga agttcatctg caccaccggc aagctgcccg
8941 tgccctggcc caccctcgtg accaccttcg gctacggcgt gcagtgcttc gcccgctacc
9001 ccgaccacat gaagcagcac gacttcttca agtccgccat gcccgaaggc tacgtccagg
9061 agcgcaccat cttcttcaag gacgacggca actacaagac ccgcgccgag gtgaagttcg
9121 agggcgacac cctggtgaac cgcatcgagc tgaagggcat cgacttcaag gaggacggca
9181 acatcctggg gcacaagctg gagtacaact acaacagcca caacgtctat atcatggccg
9241 acaagcagaa gaacggcatc aaggtgaact tcaagatccg ccacaacatc gaggacggca
9301 gcgtgcagct cgccgaccac taccagcaga acacccccat cggcgacggc cccgtgctgc

9361 tgcccgacaa ccactacctg agctaccagt ccgccctgag caaagacccc
aacgagaagc
9421 gcgatcacat ggtcctgctg gagttcgtga ccgccgccgg gatcactctc
ggcatggacg
9481 agctgtacaa gagcgggcgc c
//

Supplementary File S4


```
<?xml version="1.0" encoding="UTF-8"?>
<sbml version="1" level="2" xmlns="http://www.sbml.org/sbml/level2">
 - <model name="Arabidopsis thaliana Model V1 bZip and AKin Analysis">
   - <listOfCompartments>
       <compartment name="rootCompartment" constant="true" size="1"
         spatialDimensions="3" id="rootCompartment"/>
     </listOfCompartments>
   - <listOfSpecies>
       <species name="AC" constant="false" id="AC" boundaryCondition="false"
         hasOnlySubstanceUnits="true" initialAmount="1"
         compartment="rootCompartment"/>
       <species name="Gly" constant="false" id="Gly" boundaryCondition="false"
         hasOnlySubstanceUnits="true" initialAmount="1"
         compartment="rootCompartment"/>
       <species name="Ser" constant="false" id="Ser" boundaryCondition="false"
         hasOnlySubstanceUnits="true" initialAmount="1"
         compartment="rootCompartment"/>
       <species name="Suc" constant="false" id="Suc" boundaryCondition="false"
         hasOnlySubstanceUnits="true" initialAmount="1"
         compartment="rootCompartment"/>
       <species name="Fru" constant="false" id="Fru" boundaryCondition="false"
         hasOnlySubstanceUnits="true" initialAmount="1"
         compartment="rootCompartment"/>
       <species name="Glc" constant="false" id="Glc" boundaryCondition="false"
         hasOnlySubstanceUnits="true" initialAmount="1"
         compartment="rootCompartment"/>
       <species name="Gal" constant="false" id="Gal" boundaryCondition="false"
         hasOnlySubstanceUnits="true" initialAmount="1"
         compartment="rootCompartment"/>
       <species name="Raf" constant="false" id="Raf" boundaryCondition="false"
         hasOnlySubstanceUnits="true" initialAmount="1"
         compartment="rootCompartment"/>
       <species name="Mel" constant="false" id="Mel" boundaryCondition="false"
         hasOnlySubstanceUnits="true" initialAmount="1"
         compartment="rootCompartment"/>
       <species name="Gol" constant="false" id="Gol" boundaryCondition="false"
         hasOnlySubstanceUnits="true" initialAmount="1"
         compartment="rootCompartment"/>
       <species name="myoIn" constant="false" id="myoIn" boundaryCondition="false"
         hasOnlySubstanceUnits="true" initialAmount="1"
         compartment="rootCompartment"/>
       <species name="Asc" constant="false" id="Asc" boundaryCondition="false"
         hasOnlySubstanceUnits="true" initialAmount="1"
         compartment="rootCompartment"/>
       <species name="Shikimate" constant="false" id="Shikimate"
         boundaryCondition="false" hasOnlySubstanceUnits="true" initialAmount="1"
         compartment="rootCompartment"/>
       <species name="Phe" constant="false" id="Phe" boundaryCondition="false"
         hasOnlySubstanceUnits="true" initialAmount="1"
         compartment="rootCompartment"/>
       <species name="Tyr" constant="false" id="Tyr" boundaryCondition="false"
         hasOnlySubstanceUnits="true" initialAmount="1"
         compartment="rootCompartment"/>
```

```xml
<species name="Trp" constant="false" id="Trp" boundaryCondition="false"
    hasOnlySubstanceUnits="true" initialAmount="1"
    compartment="rootCompartment"/>
<species name="Pyr" constant="false" id="Pyr" boundaryCondition="false"
    hasOnlySubstanceUnits="true" initialAmount="1"
    compartment="rootCompartment"/>
<species name="Val" constant="false" id="Val" boundaryCondition="false"
    hasOnlySubstanceUnits="true" initialAmount="1"
    compartment="rootCompartment"/>
<species name="Leu" constant="false" id="Leu" boundaryCondition="false"
    hasOnlySubstanceUnits="true" initialAmount="1"
    compartment="rootCompartment"/>
<species name="Ala" constant="false" id="Ala" boundaryCondition="false"
    hasOnlySubstanceUnits="true" initialAmount="1"
    compartment="rootCompartment"/>
<species name="Cit" constant="false" id="Cit" boundaryCondition="false"
    hasOnlySubstanceUnits="true" initialAmount="1"
    compartment="rootCompartment"/>
<species name="Asp" constant="false" id="Asp" boundaryCondition="false"
    hasOnlySubstanceUnits="true" initialAmount="1"
    compartment="rootCompartment"/>
<species name="Met" constant="false" id="Met" boundaryCondition="false"
    hasOnlySubstanceUnits="true" initialAmount="1"
    compartment="rootCompartment"/>
<species name="Lys" constant="false" id="Lys" boundaryCondition="false"
    hasOnlySubstanceUnits="true" initialAmount="1"
    compartment="rootCompartment"/>
<species name="Asn" constant="false" id="Asn" boundaryCondition="false"
    hasOnlySubstanceUnits="true" initialAmount="1"
    compartment="rootCompartment"/>
<species name="Thr" constant="false" id="Thr" boundaryCondition="false"
    hasOnlySubstanceUnits="true" initialAmount="1"
    compartment="rootCompartment"/>
<species name="Ile" constant="false" id="Ile" boundaryCondition="false"
    hasOnlySubstanceUnits="true" initialAmount="1"
    compartment="rootCompartment"/>
<species name="KGA" constant="false" id="KGA" boundaryCondition="false"
    hasOnlySubstanceUnits="true" initialAmount="1"
    compartment="rootCompartment"/>
<species name="Succ" constant="false" id="Succ" boundaryCondition="false"
    hasOnlySubstanceUnits="true" initialAmount="1"
    compartment="rootCompartment"/>
<species name="Fum" constant="false" id="Fum" boundaryCondition="false"
    hasOnlySubstanceUnits="true" initialAmount="1"
    compartment="rootCompartment"/>
<species name="Mal" constant="false" id="Mal" boundaryCondition="false"
    hasOnlySubstanceUnits="true" initialAmount="1"
    compartment="rootCompartment"/>
<species name="Oxac" constant="false" id="Oxac" boundaryCondition="false"
    hasOnlySubstanceUnits="true" initialAmount="1"
    compartment="rootCompartment"/>
```

```
        <species name="Pro" constant="false" id="Pro" boundaryCondition="false"
            hasOnlySubstanceUnits="true" initialAmount="1"
            compartment="rootCompartment"/>
        <species name="Orn" constant="false" id="Orn" boundaryCondition="false"
            hasOnlySubstanceUnits="true" initialAmount="1"
            compartment="rootCompartment"/>
        <species name="Arg" constant="false" id="Arg" boundaryCondition="false"
            hasOnlySubstanceUnits="true" initialAmount="1"
            compartment="rootCompartment"/>
        <species name="Put" constant="false" id="Put" boundaryCondition="false"
            hasOnlySubstanceUnits="true" initialAmount="1"
            compartment="rootCompartment"/>
        <species name="Spdine" constant="false" id="Spdine" boundaryCondition="false"
            hasOnlySubstanceUnits="true" initialAmount="1"
            compartment="rootCompartment"/>
        <species name="Glu" constant="false" id="Glu" boundaryCondition="false"
            hasOnlySubstanceUnits="true" initialAmount="1"
            compartment="rootCompartment"/>
        <species name="Gln" constant="false" id="Gln" boundaryCondition="false"
            hasOnlySubstanceUnits="true" initialAmount="1"
            compartment="rootCompartment"/>
    </listOfSpecies>
  - <listOfParameters>
        <parameter name="k1" constant="true" id="k1" value="1"/>
        <parameter name="k2" constant="true" id="k2" value="1"/>
        <parameter name="k3" constant="true" id="k3" value="1"/>
        <parameter name="k4" constant="true" id="k4" value="1"/>
        <parameter name="k5" constant="true" id="k5" value="1"/>
        <parameter name="k6" constant="true" id="k6" value="1"/>
        <parameter name="k7" constant="true" id="k7" value="1"/>
        <parameter name="k8" constant="true" id="k8" value="1"/>
        <parameter name="k9" constant="true" id="k9" value="1"/>
        <parameter name="k10" constant="true" id="k10" value="1"/>
        <parameter name="k11" constant="true" id="k11" value="1"/>
        <parameter name="k12" constant="true" id="k12" value="1"/>
        <parameter name="k13" constant="true" id="k13" value="1"/>
        <parameter name="k14" constant="true" id="k14" value="1"/>
        <parameter name="k15" constant="true" id="k15" value="1"/>
        <parameter name="k16" constant="true" id="k16" value="1"/>
        <parameter name="k17" constant="true" id="k17" value="1"/>
        <parameter name="k18" constant="true" id="k18" value="1"/>
        <parameter name="k19" constant="true" id="k19" value="1"/>
        <parameter name="k20" constant="true" id="k20" value="1"/>
        <parameter name="k21" constant="true" id="k21" value="1"/>
        <parameter name="k22" constant="true" id="k22" value="1"/>
        <parameter name="k23" constant="true" id="k23" value="1"/>
        <parameter name="k24" constant="true" id="k24" value="1"/>
        <parameter name="k25" constant="true" id="k25" value="1"/>
        <parameter name="k26" constant="true" id="k26" value="1"/>
        <parameter name="k27" constant="true" id="k27" value="1"/>
        <parameter name="k28" constant="true" id="k28" value="1"/>
        <parameter name="k29" constant="true" id="k29" value="1"/>
        <parameter name="k30" constant="true" id="k30" value="1"/>
```

```
      <parameter name="k31" constant="true" id="k31" value="1"/>
      <parameter name="k32" constant="true" id="k32" value="1"/>
      <parameter name="k33" constant="true" id="k33" value="1"/>
      <parameter name="k34" constant="true" id="k34" value="1"/>
      <parameter name="k35" constant="true" id="k35" value="1"/>
      <parameter name="k36" constant="true" id="k36" value="1"/>
      <parameter name="k37" constant="true" id="k37" value="1"/>
      <parameter name="k38" constant="true" id="k38" value="1"/>
      <parameter name="k39" constant="true" id="k39" value="1"/>
      <parameter name="k39a" constant="true" id="k39a" value="1"/>
      <parameter name="k39b" constant="true" id="k39b" value="1"/>
      <parameter name="k39c" constant="true" id="k39c" value="1"/>
      <parameter name="k39d" constant="true" id="k39d" value="1"/>
      <parameter name="k40" constant="true" id="k40" value="1"/>
      <parameter name="k40a" constant="true" id="k40a" value="1"/>
      <parameter name="k41" constant="true" id="k41" value="1"/>
      <parameter name="k42" constant="true" id="k42" value="1"/>
      <parameter name="k43" constant="true" id="k43" value="1"/>
      <parameter name="k44" constant="true" id="k44" value="1"/>
      <parameter name="k45" constant="true" id="k45" value="1"/>
      <parameter name="k46" constant="true" id="k46" value="1"/>
      <parameter name="k46a" constant="true" id="k46a" value="1"/>
      <parameter name="k47" constant="true" id="k47" value="1"/>
      <parameter name="k48" constant="true" id="k48" value="1"/>
      <parameter name="k49" constant="true" id="k49" value="1"/>
      <parameter name="k50" constant="true" id="k50" value="1"/>
      <parameter name="k51" constant="true" id="k51" value="1"/>
      <parameter name="k52" constant="true" id="k52" value="1"/>
      <parameter name="k53" constant="true" id="k53" value="1"/>
    </listOfParameters>
  - <listOfReactions>
    - <reaction name="v1" id="v1" reversible="false">
      - <listOfProducts>
          <speciesReference stoichiometry="1" species="AC"/>
        </listOfProducts>
      - <kineticLaw>
        - <math xmlns="http://www.w3.org/1998/Math/MathML">
            <ci> k1 </ci>
          </math>
        </kineticLaw>
      </reaction>
    - <reaction name="v2" id="v2" reversible="false">
      - <listOfReactants>
          <speciesReference stoichiometry="1" species="AC"/>
          <speciesReference stoichiometry="1" species="Glu"/>
        </listOfReactants>
      - <listOfProducts>
          <speciesReference stoichiometry="1" species="Gly"/>
          <speciesReference stoichiometry="1" species="KGA"/>
        </listOfProducts>
      - <kineticLaw>
        - <math xmlns="http://www.w3.org/1998/Math/MathML">
          - <apply>
```

```
                <times/>
                <ci> k2 </ci>
                <ci> AC </ci>
                <ci> Glu </ci>
            </apply>
        </math>
    </kineticLaw>
</reaction>
- <reaction name="v3" id="v3" reversible="false">
  - <listOfReactants>
        <speciesReference stoichiometry="1" species="Gly"/>
    </listOfReactants>
  - <listOfProducts>
        <speciesReference stoichiometry="1" species="Ser"/>
    </listOfProducts>
  - <kineticLaw>
      - <math xmlns="http://www.w3.org/1998/Math/MathML">
          - <apply>
                <times/>
                <ci> k3 </ci>
                <ci> Gly </ci>
            </apply>
        </math>
    </kineticLaw>
</reaction>
- <reaction name="v4" id="v4" reversible="false">
  - <listOfReactants>
        <speciesReference stoichiometry="1" species="Ser"/>
    </listOfReactants>
  - <kineticLaw>
      - <math xmlns="http://www.w3.org/1998/Math/MathML">
          - <apply>
                <times/>
                <ci> k4 </ci>
                <ci> Ser </ci>
            </apply>
        </math>
    </kineticLaw>
</reaction>
- <reaction name="v5" id="v5" reversible="false">
  - <listOfReactants>
        <speciesReference stoichiometry="1" species="AC"/>
    </listOfReactants>
  - <listOfProducts>
        <speciesReference stoichiometry="1" species="Shikimate"/>
    </listOfProducts>
  - <kineticLaw>
      - <math xmlns="http://www.w3.org/1998/Math/MathML">
          - <apply>
                <times/>
                <ci> k5 </ci>
                <ci> AC </ci>
            </apply>
```

```
        </math>
      </kineticLaw>
    </reaction>
  - <reaction name="v6" id="v6" reversible="false">
    - <listOfReactants>
        <speciesReference stoichiometry="1" species="Shikimate"/>
        <speciesReference stoichiometry="1" species="Glu"/>
      </listOfReactants>
    - <listOfProducts>
        <speciesReference stoichiometry="1" species="Phe"/>
        <speciesReference stoichiometry="1" species="KGA"/>
      </listOfProducts>
    - <kineticLaw>
      - <math xmlns="http://www.w3.org/1998/Math/MathML">
        - <apply>
            <times/>
            <ci> k6 </ci>
            <ci> Shikimate </ci>
            <ci> Glu </ci>
          </apply>
        </math>
      </kineticLaw>
    </reaction>
  - <reaction name="v7" id="v7" reversible="false">
    - <listOfReactants>
        <speciesReference stoichiometry="1" species="Phe"/>
      </listOfReactants>
    - <kineticLaw>
      - <math xmlns="http://www.w3.org/1998/Math/MathML">
        - <apply>
            <times/>
            <ci> k7 </ci>
            <ci> Phe </ci>
          </apply>
        </math>
      </kineticLaw>
    </reaction>
  - <reaction name="v8" id="v8" reversible="false">
    - <listOfReactants>
        <speciesReference stoichiometry="1" species="Shikimate"/>
        <speciesReference stoichiometry="1" species="Glu"/>
      </listOfReactants>
    - <listOfProducts>
        <speciesReference stoichiometry="1" species="Tyr"/>
        <speciesReference stoichiometry="1" species="KGA"/>
      </listOfProducts>
    - <kineticLaw>
      - <math xmlns="http://www.w3.org/1998/Math/MathML">
        - <apply>
            <times/>
            <ci> k8 </ci>
            <ci> Shikimate </ci>
            <ci> Glu </ci>
```

```
            </apply>
          </math>
        </kineticLaw>
      </reaction>
    - <reaction name="v9" id="v9" reversible="false">
      - <listOfReactants>
          <speciesReference stoichiometry="1" species="Tyr"/>
        </listOfReactants>
      - <kineticLaw>
        - <math xmlns="http://www.w3.org/1998/Math/MathML">
          - <apply>
              <times/>
              <ci> k9 </ci>
              <ci> Tyr </ci>
            </apply>
          </math>
        </kineticLaw>
      </reaction>
    - <reaction name="v10" id="v10" reversible="false">
      - <listOfReactants>
          <speciesReference stoichiometry="1" species="Ser"/>
          <speciesReference stoichiometry="1" species="Shikimate"/>
          <speciesReference stoichiometry="1" species="Gln"/>
        </listOfReactants>
      - <listOfProducts>
          <speciesReference stoichiometry="1" species="AC"/>
          <speciesReference stoichiometry="1" species="Trp"/>
          <speciesReference stoichiometry="1" species="Pyr"/>
          <speciesReference stoichiometry="1" species="Glu"/>
        </listOfProducts>
      - <kineticLaw>
        - <math xmlns="http://www.w3.org/1998/Math/MathML">
          - <apply>
              <times/>
              <ci> k10 </ci>
              <ci> Shikimate </ci>
              <ci> Gln </ci>
              <ci> Ser </ci>
            </apply>
          </math>
        </kineticLaw>
      </reaction>
    - <reaction name="v11" id="v11" reversible="false">
      - <listOfReactants>
          <speciesReference stoichiometry="1" species="Trp"/>
        </listOfReactants>
      - <kineticLaw>
        - <math xmlns="http://www.w3.org/1998/Math/MathML">
          - <apply>
              <times/>
              <ci> k11 </ci>
              <ci> Trp </ci>
            </apply>
```

```
            </math>
        </kineticLaw>
    </reaction>
-   <reaction name="v12" id="v12" reversible="false">
    -   <listOfReactants>
            <speciesReference stoichiometry="1" species="AC"/>
        </listOfReactants>
    -   <listOfProducts>
            <speciesReference stoichiometry="1" species="Suc"/>
        </listOfProducts>
    -   <kineticLaw>
        -   <math xmlns="http://www.w3.org/1998/Math/MathML">
            -   <apply>
                    <times/>
                    <ci> k12 </ci>
                    <ci> AC </ci>
                </apply>
            </math>
        </kineticLaw>
    </reaction>
-   <reaction name="v13" id="v13" reversible="false">
    -   <listOfReactants>
            <speciesReference stoichiometry="1" species="Suc"/>
            <speciesReference stoichiometry="1" species="Gol"/>
        </listOfReactants>
    -   <listOfProducts>
            <speciesReference stoichiometry="1" species="Raf"/>
        </listOfProducts>
    -   <kineticLaw>
        -   <math xmlns="http://www.w3.org/1998/Math/MathML">
            -   <apply>
                    <times/>
                    <ci> k13 </ci>
                    <ci> Suc </ci>
                    <ci> Gol </ci>
                </apply>
            </math>
        </kineticLaw>
    </reaction>
-   <reaction name="v14" id="v14" reversible="false">
    -   <listOfReactants>
            <speciesReference stoichiometry="1" species="Raf"/>
        </listOfReactants>
    -   <listOfProducts>
            <speciesReference stoichiometry="1" species="Fru"/>
            <speciesReference stoichiometry="1" species="Mel"/>
        </listOfProducts>
    -   <kineticLaw>
        -   <math xmlns="http://www.w3.org/1998/Math/MathML">
            -   <apply>
                    <times/>
                    <ci> k14 </ci>
                    <ci> Raf </ci>
```

```
          </apply>
        </math>
      </kineticLaw>
    </reaction>
  - <reaction name="v15" id="v15" reversible="false">
    - <listOfReactants>
        <speciesReference stoichiometry="1" species="Mel"/>
      </listOfReactants>
    - <listOfProducts>
        <speciesReference stoichiometry="1" species="Glc"/>
        <speciesReference stoichiometry="1" species="Gal"/>
      </listOfProducts>
    - <kineticLaw>
      - <math xmlns="http://www.w3.org/1998/Math/MathML">
        - <apply>
            <times/>
            <ci> k15 </ci>
            <ci> Mel </ci>
          </apply>
        </math>
      </kineticLaw>
    </reaction>
  - <reaction name="v16" id="v16" reversible="false">
    - <listOfReactants>
        <speciesReference stoichiometry="1" species="Gal"/>
      </listOfReactants>
    - <listOfProducts>
        <speciesReference stoichiometry="1" species="AC"/>
      </listOfProducts>
    - <kineticLaw>
      - <math xmlns="http://www.w3.org/1998/Math/MathML">
        - <apply>
            <times/>
            <ci> k16 </ci>
            <ci> Gal </ci>
          </apply>
        </math>
      </kineticLaw>
    </reaction>
  - <reaction name="v17" id="v17" reversible="false">
    - <listOfReactants>
        <speciesReference stoichiometry="1" species="Glc"/>
      </listOfReactants>
    - <listOfProducts>
        <speciesReference stoichiometry="1" species="AC"/>
      </listOfProducts>
    - <kineticLaw>
      - <math xmlns="http://www.w3.org/1998/Math/MathML">
        - <apply>
            <times/>
            <ci> k17 </ci>
            <ci> Glc </ci>
          </apply>
```

```
        </math>
      </kineticLaw>
    </reaction>
  - <reaction name="v18" id="v18" reversible="false">
    - <listOfReactants>
        <speciesReference stoichiometry="1" species="Suc"/>
      </listOfReactants>
    - <listOfProducts>
        <speciesReference stoichiometry="1" species="Fru"/>
        <speciesReference stoichiometry="1" species="Glc"/>
      </listOfProducts>
    - <kineticLaw>
      - <math xmlns="http://www.w3.org/1998/Math/MathML">
        - <apply>
            <times/>
            <ci> k18 </ci>
            <ci> Suc </ci>
          </apply>
        </math>
      </kineticLaw>
    </reaction>
  - <reaction name="v19" id="v19" reversible="false">
    - <listOfReactants>
        <speciesReference stoichiometry="1" species="Fru"/>
      </listOfReactants>
    - <listOfProducts>
        <speciesReference stoichiometry="1" species="AC"/>
      </listOfProducts>
    - <kineticLaw>
      - <math xmlns="http://www.w3.org/1998/Math/MathML">
        - <apply>
            <times/>
            <ci> k19 </ci>
            <ci> Fru </ci>
          </apply>
        </math>
      </kineticLaw>
    </reaction>
  - <reaction name="v20" id="v20" reversible="false">
    - <listOfReactants>
        <speciesReference stoichiometry="1" species="AC"/>
        <speciesReference stoichiometry="1" species="myoIn"/>
      </listOfReactants>
    - <listOfProducts>
        <speciesReference stoichiometry="1" species="Gol"/>
      </listOfProducts>
    - <kineticLaw>
      - <math xmlns="http://www.w3.org/1998/Math/MathML">
        - <apply>
            <times/>
            <ci> k20 </ci>
            <ci> myoIn </ci>
            <ci> AC </ci>
```

```
          </apply>
        </math>
      </kineticLaw>
    </reaction>
  - <reaction name="v21" id="v21" reversible="false">
    - <listOfReactants>
        <speciesReference stoichiometry="1" species="AC"/>
        <speciesReference stoichiometry="1" species="myoIn"/>
      </listOfReactants>
    - <listOfProducts>
        <speciesReference stoichiometry="1" species="Asc"/>
      </listOfProducts>
    - <kineticLaw>
      - <math xmlns="http://www.w3.org/1998/Math/MathML">
        - <apply>
            <times/>
            <ci> k21 </ci>
            <ci> myoIn </ci>
            <ci> AC </ci>
          </apply>
        </math>
      </kineticLaw>
    </reaction>
  - <reaction name="v22" id="v22" reversible="false">
    - <listOfReactants>
        <speciesReference stoichiometry="1" species="Asc"/>
      </listOfReactants>
    - <kineticLaw>
      - <math xmlns="http://www.w3.org/1998/Math/MathML">
        - <apply>
            <times/>
            <ci> k22 </ci>
            <ci> Asc </ci>
          </apply>
        </math>
      </kineticLaw>
    </reaction>
  - <reaction name="v23" id="v23" reversible="false">
    - <listOfReactants>
        <speciesReference stoichiometry="1" species="AC"/>
      </listOfReactants>
    - <listOfProducts>
        <speciesReference stoichiometry="1" species="Pyr"/>
      </listOfProducts>
    - <kineticLaw>
      - <math xmlns="http://www.w3.org/1998/Math/MathML">
        - <apply>
            <times/>
            <ci> k23 </ci>
            <ci> AC </ci>
          </apply>
        </math>
      </kineticLaw>
```

```
  </reaction>
- <reaction name="v24" id="v24" reversible="false">
  - <listOfReactants>
      <speciesReference stoichiometry="1" species="Pyr"/>
      <speciesReference stoichiometry="1" species="Glu"/>
    </listOfReactants>
  - <listOfProducts>
      <speciesReference stoichiometry="1" species="Val"/>
      <speciesReference stoichiometry="1" species="KGA"/>
    </listOfProducts>
  - <kineticLaw>
    - <math xmlns="http://www.w3.org/1998/Math/MathML">
      - <apply>
          <times/>
          <ci> k24 </ci>
          <ci> Pyr </ci>
          <ci> Glu </ci>
        </apply>
      </math>
    </kineticLaw>
  </reaction>
- <reaction name="v25" id="v25" reversible="false">
  - <listOfReactants>
      <speciesReference stoichiometry="1" species="Pyr"/>
      <speciesReference stoichiometry="1" species="Val"/>
    </listOfReactants>
  - <listOfProducts>
      <speciesReference stoichiometry="1" species="Ala"/>
    </listOfProducts>
  - <kineticLaw>
    - <math xmlns="http://www.w3.org/1998/Math/MathML">
      - <apply>
          <times/>
          <ci> k25 </ci>
          <ci> Pyr </ci>
        </apply>
      </math>
    </kineticLaw>
  </reaction>
- <reaction name="v26" id="v26" reversible="false">
  - <listOfReactants>
      <speciesReference stoichiometry="1" species="Val"/>
    </listOfReactants>
  - <kineticLaw>
    - <math xmlns="http://www.w3.org/1998/Math/MathML">
      - <apply>
          <times/>
          <ci> k26 </ci>
          <ci> Val </ci>
        </apply>
      </math>
    </kineticLaw>
  </reaction>
```

```
- <reaction name="v27" id="v27" reversible="false">
  - <listOfReactants>
      <speciesReference stoichiometry="1" species="Pyr"/>
      <speciesReference stoichiometry="1" species="Glu"/>
    </listOfReactants>
  - <listOfProducts>
      <speciesReference stoichiometry="1" species="Leu"/>
      <speciesReference stoichiometry="1" species="KGA"/>
    </listOfProducts>
  - <kineticLaw>
    - <math xmlns="http://www.w3.org/1998/Math/MathML">
      - <apply>
          <times/>
          <ci> k27 </ci>
          <ci> Pyr </ci>
          <ci> Glu </ci>
        </apply>
      </math>
    </kineticLaw>
  </reaction>
- <reaction name="v28" id="v28" reversible="false">
  - <listOfReactants>
      <speciesReference stoichiometry="1" species="Leu"/>
    </listOfReactants>
  - <kineticLaw>
    - <math xmlns="http://www.w3.org/1998/Math/MathML">
      - <apply>
          <times/>
          <ci> k28 </ci>
          <ci> Leu </ci>
        </apply>
      </math>
    </kineticLaw>
  </reaction>
- <reaction name="v29" id="v29" reversible="false">
  - <listOfReactants>
      <speciesReference stoichiometry="1" species="Ala"/>
    </listOfReactants>
  - <kineticLaw>
    - <math xmlns="http://www.w3.org/1998/Math/MathML">
      - <apply>
          <times/>
          <ci> k29 </ci>
          <ci> Ala </ci>
        </apply>
      </math>
    </kineticLaw>
  </reaction>
- <reaction name="v30" id="v30" reversible="false">
  - <listOfReactants>
      <speciesReference stoichiometry="1" species="Pyr"/>
      <speciesReference stoichiometry="1" species="Oxac"/>
    </listOfReactants>
```

```
    - <listOfProducts>
          <speciesReference stoichiometry="1" species="Cit"/>
      </listOfProducts>
    - <kineticLaw>
        - <math xmlns="http://www.w3.org/1998/Math/MathML">
            - <apply>
                  <times/>
                  <ci> k30 </ci>
                  <ci> Pyr </ci>
                  <ci> Oxac </ci>
              </apply>
          </math>
      </kineticLaw>
  </reaction>
- <reaction name="v31" id="v31" reversible="false">
    - <listOfReactants>
          <speciesReference stoichiometry="1" species="Cit"/>
      </listOfReactants>
    - <listOfProducts>
          <speciesReference stoichiometry="1" species="KGA"/>
      </listOfProducts>
    - <kineticLaw>
        - <math xmlns="http://www.w3.org/1998/Math/MathML">
            - <apply>
                  <times/>
                  <ci> k31 </ci>
                  <ci> Cit </ci>
              </apply>
          </math>
      </kineticLaw>
  </reaction>
- <reaction name="v32" id="v32" reversible="false">
    - <listOfReactants>
          <speciesReference stoichiometry="1" species="KGA"/>
      </listOfReactants>
    - <listOfProducts>
          <speciesReference stoichiometry="1" species="Succ"/>
      </listOfProducts>
    - <kineticLaw>
        - <math xmlns="http://www.w3.org/1998/Math/MathML">
            - <apply>
                  <times/>
                  <ci> k32 </ci>
                  <ci> KGA </ci>
              </apply>
          </math>
      </kineticLaw>
  </reaction>
- <reaction name="v33" id="v33" reversible="false">
    - <listOfReactants>
          <speciesReference stoichiometry="1" species="Succ"/>
      </listOfReactants>
    - <listOfProducts>
```

<speciesReference stoichiometry="**1**" species="**Fum**"/>
</listOfProducts>
- <kineticLaw>
- <math xmlns="**http://www.w3.org/1998/Math/MathML**">
- <apply>
<times/>
<ci> k33 </ci>
<ci> Succ </ci>
</apply>
</math>
</kineticLaw>
</reaction>
- <reaction name="**v34**" id="**v34**" reversible="**false**">
- <listOfReactants>
<speciesReference stoichiometry="**1**" species="**Fum**"/>
</listOfReactants>
- <listOfProducts>
<speciesReference stoichiometry="**1**" species="**Mal**"/>
</listOfProducts>
- <kineticLaw>
- <math xmlns="**http://www.w3.org/1998/Math/MathML**">
- <apply>
<times/>
<ci> k34 </ci>
<ci> Fum </ci>
</apply>
</math>
</kineticLaw>
</reaction>
- <reaction name="**v35**" id="**v35**" reversible="**false**">
- <listOfReactants>
<speciesReference stoichiometry="**1**" species="**Mal**"/>
</listOfReactants>
- <listOfProducts>
<speciesReference stoichiometry="**1**" species="**Oxac**"/>
</listOfProducts>
- <kineticLaw>
- <math xmlns="**http://www.w3.org/1998/Math/MathML**">
- <apply>
<times/>
<ci> k35 </ci>
<ci> Mal </ci>
</apply>
</math>
</kineticLaw>
</reaction>
- <reaction name="**v36**" id="**v36**" reversible="**false**">
- <listOfReactants>
<speciesReference stoichiometry="**1**" species="**Oxac**"/>
<speciesReference stoichiometry="**1**" species="**Glu**"/>
</listOfReactants>
- <listOfProducts>
<speciesReference stoichiometry="**1**" species="**Asp**"/>

<speciesReference stoichiometry="**1**" species="**KGA**"/>
</listOfProducts>
- <kineticLaw>
- <math xmlns="**http://www.w3.org/1998/Math/MathML**">
- <apply>
<times/>
<ci> k36 </ci>
<ci> Oxac </ci>
<ci> Glu </ci>
</apply>
</math>
</kineticLaw>
</reaction>
- <reaction name="**v37**" id="**v37**" reversible="**false**">
- <listOfReactants>
<speciesReference stoichiometry="**1**" species="**Asp**"/>
<speciesReference stoichiometry="**1**" species="**Gln**"/>
</listOfReactants>
- <listOfProducts>
<speciesReference stoichiometry="**1**" species="**Asn**"/>
<speciesReference stoichiometry="**1**" species="**Glu**"/>
</listOfProducts>
- <kineticLaw>
- <math xmlns="**http://www.w3.org/1998/Math/MathML**">
- <apply>
<times/>
<ci> k37 </ci>
<ci> Asp </ci>
<ci> Gln </ci>
</apply>
</math>
</kineticLaw>
</reaction>
- <reaction name="**v38**" id="**v38**" reversible="**false**">
- <listOfReactants>
<speciesReference stoichiometry="**1**" species="**Asn**"/>
</listOfReactants>
- <kineticLaw>
- <math xmlns="**http://www.w3.org/1998/Math/MathML**">
- <apply>
<times/>
<ci> k38 </ci>
<ci> Asn </ci>
</apply>
</math>
</kineticLaw>
</reaction>
- <reaction name="**v39**" id="**v39**" reversible="**false**">
- <listOfReactants>
<speciesReference stoichiometry="**1**" species="**Asp**"/>
</listOfReactants>
- <listOfProducts>
<speciesReference stoichiometry="**1**" species="**Thr**"/>

```
      </listOfProducts>
    - <kineticLaw>
      - <math xmlns="http://www.w3.org/1998/Math/MathML">
        - <apply>
            <times/>
            <ci> k39 </ci>
            <ci> Asp </ci>
          </apply>
        </math>
      </kineticLaw>
    </reaction>
  - <reaction name="v39a" id="v39a" reversible="false">
    - <listOfReactants>
        <speciesReference stoichiometry="1" species="Asp"/>
      </listOfReactants>
    - <listOfProducts>
        <speciesReference stoichiometry="1" species="Lys"/>
      </listOfProducts>
    - <kineticLaw>
      - <math xmlns="http://www.w3.org/1998/Math/MathML">
        - <apply>
            <times/>
            <ci> k39a </ci>
            <ci> Asp </ci>
          </apply>
        </math>
      </kineticLaw>
    </reaction>
  - <reaction name="v39b" id="v39b" reversible="false">
    - <listOfReactants>
        <speciesReference stoichiometry="1" species="Lys"/>
      </listOfReactants>
    - <kineticLaw>
      - <math xmlns="http://www.w3.org/1998/Math/MathML">
        - <apply>
            <times/>
            <ci> k39b </ci>
            <ci> Lys </ci>
          </apply>
        </math>
      </kineticLaw>
    </reaction>
  - <reaction name="v39c" id="v39c" reversible="false">
    - <listOfReactants>
        <speciesReference stoichiometry="1" species="Asp"/>
      </listOfReactants>
    - <listOfProducts>
        <speciesReference stoichiometry="1" species="Met"/>
      </listOfProducts>
    - <kineticLaw>
      - <math xmlns="http://www.w3.org/1998/Math/MathML">
        - <apply>
            <times/>
```

```
            <ci> k39c </ci>
            <ci> Asp </ci>
          </apply>
        </math>
      </kineticLaw>
    </reaction>
  - <reaction name="v39d" id="v39d" reversible="false">
    - <listOfReactants>
        <speciesReference stoichiometry="1" species="Met"/>
      </listOfReactants>
    - <kineticLaw>
      - <math xmlns="http://www.w3.org/1998/Math/MathML">
        - <apply>
            <times/>
            <ci> k39d </ci>
            <ci> Met </ci>
          </apply>
        </math>
      </kineticLaw>
    </reaction>
  - <reaction name="v40" id="v40" reversible="false">
    - <listOfReactants>
        <speciesReference stoichiometry="1" species="Thr"/>
        <speciesReference stoichiometry="1" species="Glu"/>
      </listOfReactants>
    - <listOfProducts>
        <speciesReference stoichiometry="1" species="Ile"/>
        <speciesReference stoichiometry="1" species="KGA"/>
      </listOfProducts>
    - <kineticLaw>
      - <math xmlns="http://www.w3.org/1998/Math/MathML">
        - <apply>
            <times/>
            <ci> k40 </ci>
            <ci> Thr </ci>
            <ci> Glu </ci>
          </apply>
        </math>
      </kineticLaw>
    </reaction>
  - <reaction name="v40a" id="v40a" reversible="false">
    - <listOfReactants>
        <speciesReference stoichiometry="1" species="Ile"/>
        <speciesReference stoichiometry="1" species="KGA"/>
      </listOfReactants>
    - <listOfProducts>
        <speciesReference stoichiometry="1" species="Glu"/>
      </listOfProducts>
    - <kineticLaw>
      - <math xmlns="http://www.w3.org/1998/Math/MathML">
        - <apply>
            <times/>
            <ci> k40a </ci>
```

```
            <ci> Ile </ci>
            <ci> KGA </ci>
          </apply>
        </math>
      </kineticLaw>
    </reaction>
  - <reaction name="v41" id="v41" reversible="false">
    - <listOfReactants>
        <speciesReference stoichiometry="1" species="KGA"/>
      </listOfReactants>
    - <listOfProducts>
        <speciesReference stoichiometry="1" species="Glu"/>
      </listOfProducts>
    - <kineticLaw>
      - <math xmlns="http://www.w3.org/1998/Math/MathML">
        - <apply>
            <times/>
            <ci> k41 </ci>
            <ci> KGA </ci>
          </apply>
        </math>
      </kineticLaw>
    </reaction>
  - <reaction name="v42" id="v42" reversible="false">
    - <listOfReactants>
        <speciesReference stoichiometry="1" species="Glu"/>
      </listOfReactants>
    - <listOfProducts>
        <speciesReference stoichiometry="1" species="Gln"/>
      </listOfProducts>
    - <kineticLaw>
      - <math xmlns="http://www.w3.org/1998/Math/MathML">
        - <apply>
            <times/>
            <ci> k42 </ci>
            <ci> Glu </ci>
          </apply>
        </math>
      </kineticLaw>
    </reaction>
  - <reaction name="v43" id="v43" reversible="false">
    - <listOfReactants>
        <speciesReference stoichiometry="1" species="Gln"/>
      </listOfReactants>
    - <kineticLaw>
      - <math xmlns="http://www.w3.org/1998/Math/MathML">
        - <apply>
            <times/>
            <ci> k43 </ci>
            <ci> Gln </ci>
          </apply>
        </math>
      </kineticLaw>
```

```
    </reaction>
  - <reaction name="v44" id="v44" reversible="false">
    - <listOfReactants>
        <speciesReference stoichiometry="1" species="Glu"/>
      </listOfReactants>
    - <listOfProducts>
        <speciesReference stoichiometry="1" species="Pro"/>
      </listOfProducts>
    - <kineticLaw>
      - <math xmlns="http://www.w3.org/1998/Math/MathML">
        - <apply>
            <times/>
            <ci> k44 </ci>
            <ci> Glu </ci>
          </apply>
        </math>
      </kineticLaw>
    </reaction>
  - <reaction name="v45" id="v45" reversible="false">
    - <listOfReactants>
        <speciesReference stoichiometry="1" species="Pro"/>
      </listOfReactants>
    - <kineticLaw>
      - <math xmlns="http://www.w3.org/1998/Math/MathML">
        - <apply>
            <times/>
            <ci> k45 </ci>
            <ci> Pro </ci>
          </apply>
        </math>
      </kineticLaw>
    </reaction>
  - <reaction name="v46" id="v46" reversible="false">
    - <listOfReactants>
        <speciesReference stoichiometry="2" species="Glu"/>
      </listOfReactants>
    - <listOfProducts>
        <speciesReference stoichiometry="1" species="KGA"/>
        <speciesReference stoichiometry="1" species="Orn"/>
      </listOfProducts>
    - <kineticLaw>
      - <math xmlns="http://www.w3.org/1998/Math/MathML">
        - <apply>
            <times/>
            <ci> k46 </ci>
            <ci> Glu </ci>
          </apply>
        </math>
      </kineticLaw>
    </reaction>
  - <reaction name="v46a" id="v46a" reversible="false">
    - <listOfReactants>
        <speciesReference stoichiometry="1" species="Asp"/>
```

```
        <speciesReference stoichiometry="1" species="Orn"/>
      </listOfReactants>
    - <listOfProducts>
        <speciesReference stoichiometry="1" species="Fum"/>
        <speciesReference stoichiometry="1" species="Arg"/>
      </listOfProducts>
    - <kineticLaw>
      - <math xmlns="http://www.w3.org/1998/Math/MathML">
        - <apply>
            <times/>
            <ci> k46a </ci>
            <ci> Orn </ci>
            <ci> Asp </ci>
          </apply>
        </math>
      </kineticLaw>
    </reaction>
  - <reaction name="v47" id="v47" reversible="false">
    - <listOfReactants>
        <speciesReference stoichiometry="1" species="Arg"/>
      </listOfReactants>
    - <listOfProducts>
        <speciesReference stoichiometry="1" species="Put"/>
      </listOfProducts>
    - <kineticLaw>
      - <math xmlns="http://www.w3.org/1998/Math/MathML">
        - <apply>
            <times/>
            <ci> k47 </ci>
            <ci> Arg </ci>
          </apply>
        </math>
      </kineticLaw>
    </reaction>
  - <reaction name="v48" id="v48" reversible="false">
    - <listOfReactants>
        <speciesReference stoichiometry="1" species="Put"/>
      </listOfReactants>
    - <listOfProducts>
        <speciesReference stoichiometry="1" species="Spdine"/>
      </listOfProducts>
    - <kineticLaw>
      - <math xmlns="http://www.w3.org/1998/Math/MathML">
        - <apply>
            <times/>
            <ci> k48 </ci>
            <ci> Put </ci>
          </apply>
        </math>
      </kineticLaw>
    </reaction>
  - <reaction name="v49" id="v49" reversible="false">
    - <listOfReactants>
```

```
        <speciesReference stoichiometry="1" species="Put"/>
      </listOfReactants>
    - <listOfProducts>
        <speciesReference stoichiometry="1" species="Succ"/>
      </listOfProducts>
    - <kineticLaw>
      - <math xmlns="http://www.w3.org/1998/Math/MathML">
        - <apply>
            <times/>
            <ci> k49 </ci>
            <ci> Put </ci>
          </apply>
        </math>
      </kineticLaw>
    </reaction>
  - <reaction name="v50" id="v50" reversible="false">
    - <listOfReactants>
        <speciesReference stoichiometry="1" species="Spdine"/>
      </listOfReactants>
    - <listOfProducts>
        <speciesReference stoichiometry="1" species="Put"/>
      </listOfProducts>
    - <kineticLaw>
      - <math xmlns="http://www.w3.org/1998/Math/MathML">
        - <apply>
            <times/>
            <ci> k50 </ci>
            <ci> Spdine </ci>
          </apply>
        </math>
      </kineticLaw>
    </reaction>
  - <reaction name="v51" id="v51" reversible="false">
    - <listOfReactants>
        <speciesReference stoichiometry="1" species="Spdine"/>
      </listOfReactants>
    - <kineticLaw>
      - <math xmlns="http://www.w3.org/1998/Math/MathML">
        - <apply>
            <times/>
            <ci> k51 </ci>
            <ci> Spdine </ci>
          </apply>
        </math>
      </kineticLaw>
    </reaction>
  - <reaction name="v52" id="v52" reversible="false">
    - <listOfReactants>
        <speciesReference stoichiometry="1" species="Suc"/>
      </listOfReactants>
    - <kineticLaw>
      - <math xmlns="http://www.w3.org/1998/Math/MathML">
        - <apply>
```

<times/>
<ci> k52 </ci>
<ci> Suc </ci>
</apply>
</math>
</kineticLaw>
</reaction>
- <reaction name="**v53**" id="**v53**" reversible="**false**">
- <listOfProducts>
<speciesReference stoichiometry="**1**" species="**myoIn**"/>
</listOfProducts>
- <listOfModifiers>
<modifierSpeciesReference species="**AC**"/>
</listOfModifiers>
- <kineticLaw>
- <math xmlns="**http://www.w3.org/1998/Math/MathML**">
- <apply>
<times/>
<ci> k53 </ci>
<ci> AC </ci>
</apply>
</math>
</kineticLaw>
</reaction>
</listOfReactions>
</model>
</sbml>
